# Correction: Insect Biometrics: Optoacoustic Signal Processing and Its Applications to Remote Monitoring of McPhail Type Traps

**DOI:** 10.1371/journal.pone.0145024

**Published:** 2015-12-09

**Authors:** The PLOS ONE Staff

## Notice of Republication

This article was republished on November 23, 2015, to correct the Supporting Information files, which were labeled incorrectly. In addition, Fig 4 was replaced with a corrected version. The publisher apologizes for the errors. Please download this article and its Supporting Information files again to view the correct versions.

## Supporting Information

S1 File Originally published, uncorrected article. (PDF)

S2 File Republished, corrected article. (PDF)

## References

[1] Potamitis I, Rigakis I, Fysarakis K (2015) Insect Biometrics: Optoacoustic Signal Processing and Its Applications to Remote Monitoring of McPhail Type Traps. PLoS ONE 10(11): e0140474 doi: 10.1371/journal.pone.0140474 26544845 10.1371/journal.pone.0140474 PMC4636391

