# Supplementary figures and images for: Insect Biometrics: Optoacoustic Signal Processing and Its Applications to Remote Monitoring of McPhail Type Traps
Source: PLoS One. 2015 Nov 6;10(11):e0140474. doi: 10.1371/journal.pone.0140474 (PMC4636391; doi:10.1371/journal.pone.0140474)

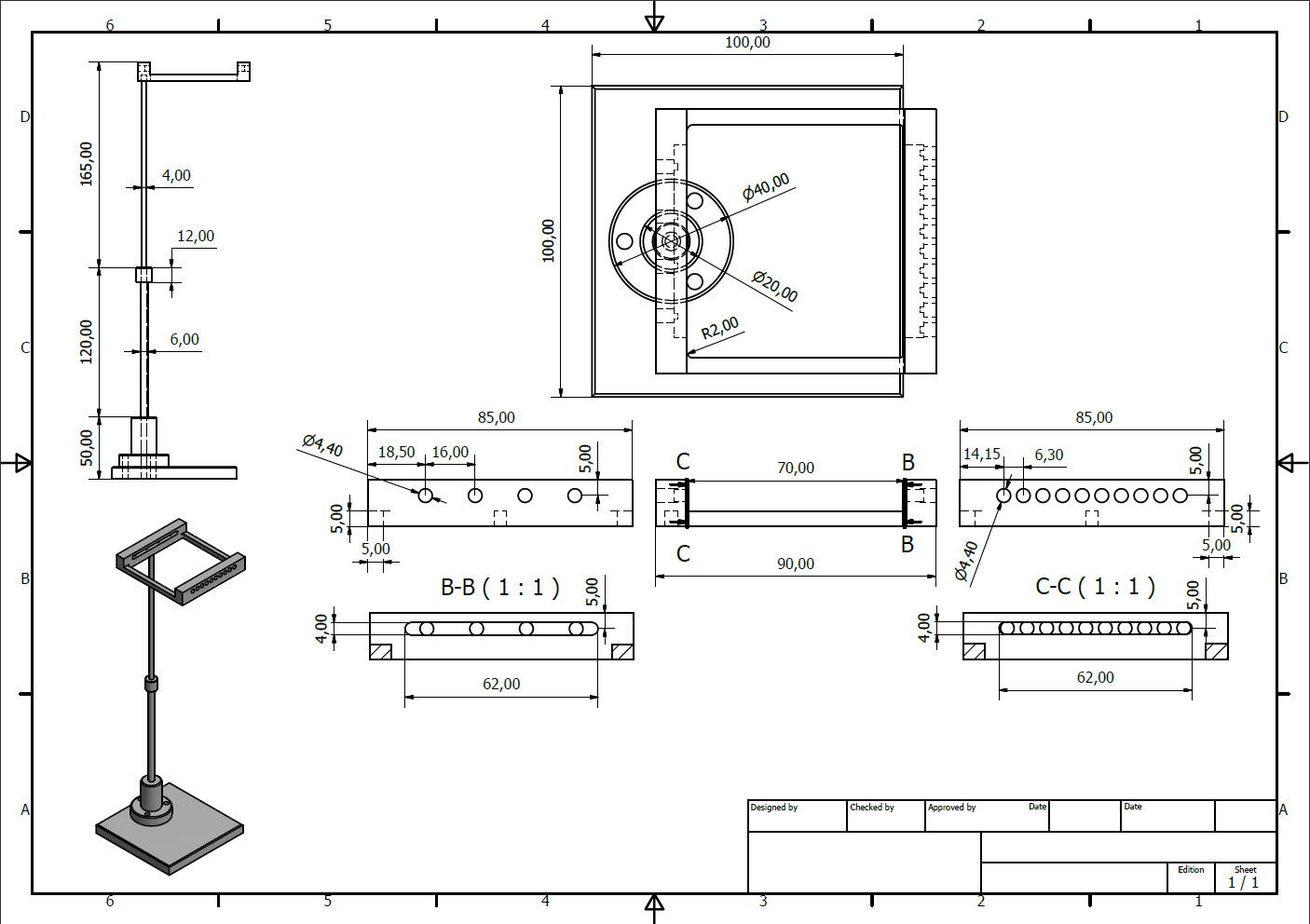

Supplement: S1 Fig — (TIF) [file pone.0140474.s001.tif]

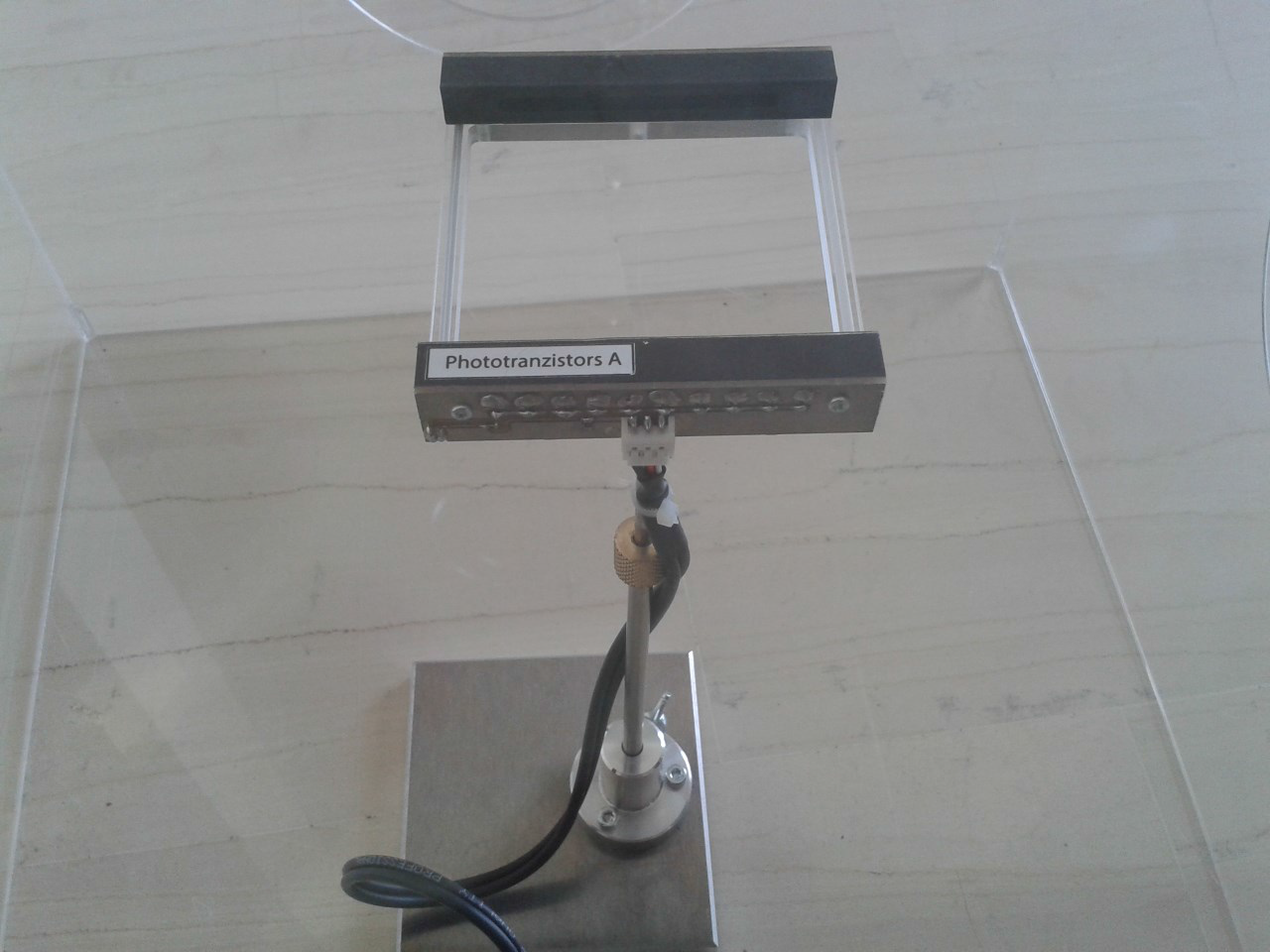

Supplement: S2 Fig — (TIF) [file pone.0140474.s002.tif]

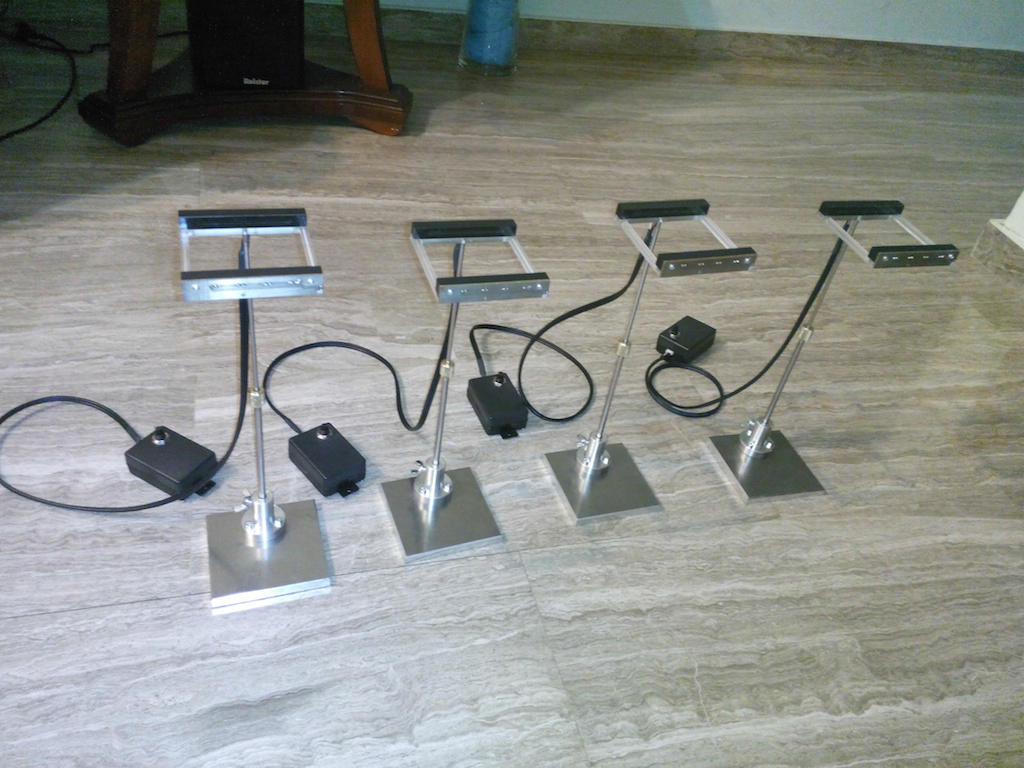

Supplement: S3 Fig — (TIF) [file pone.0140474.s003.tif]

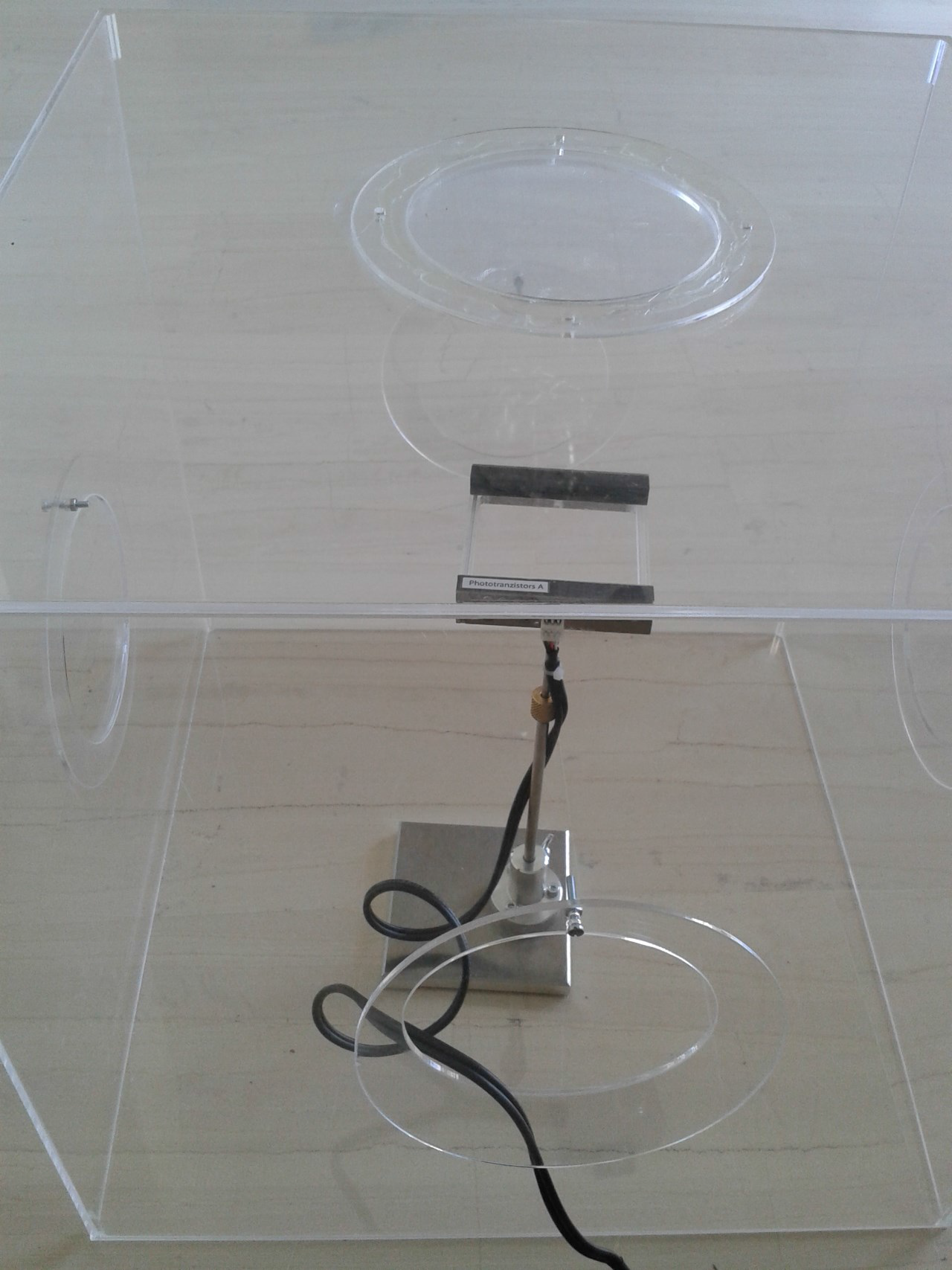

Supplement: S4 Fig — (TIF) [file pone.0140474.s004.tif]

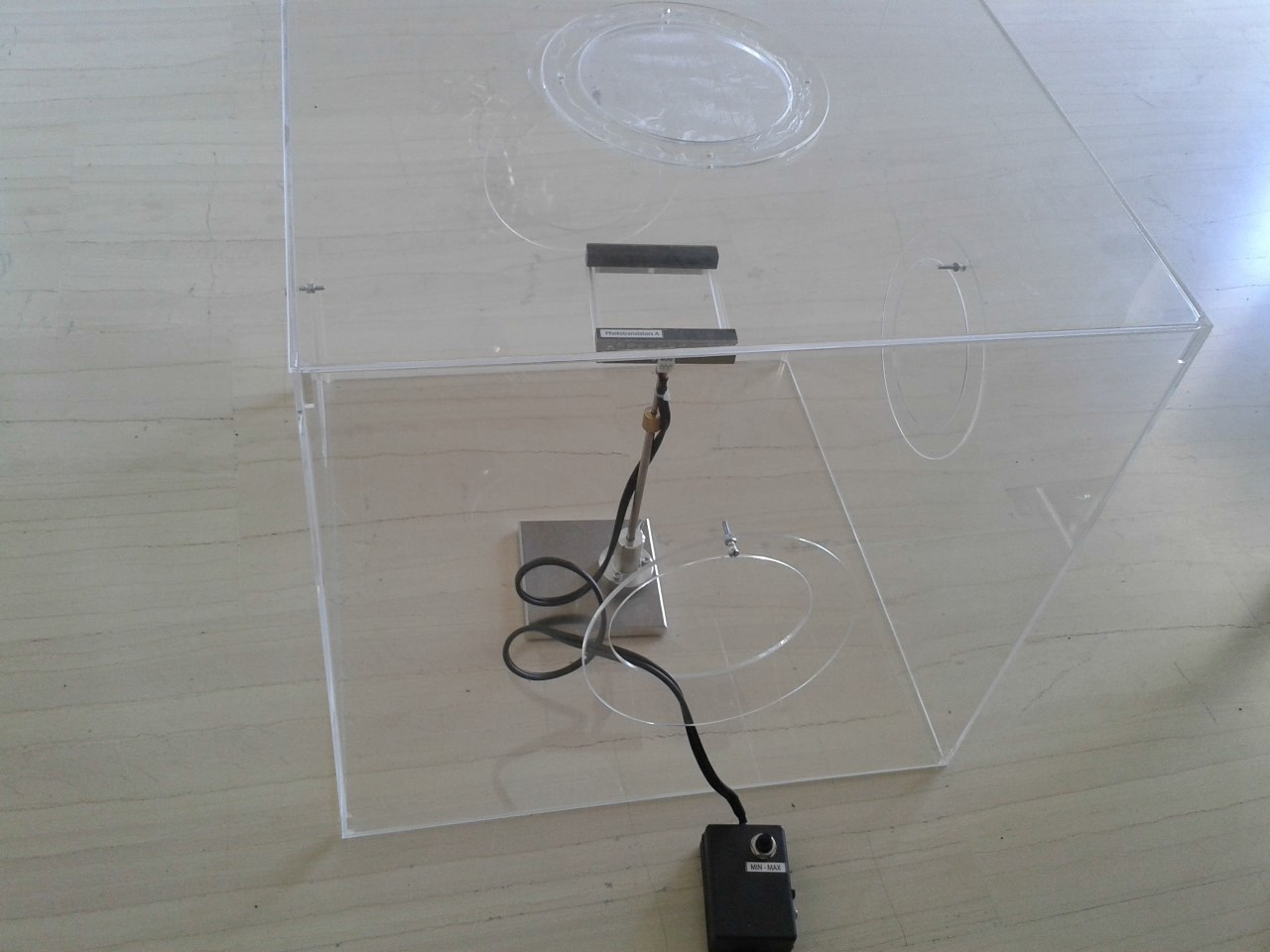

Supplement: S5 Fig — (TIF) [file pone.0140474.s005.tif]

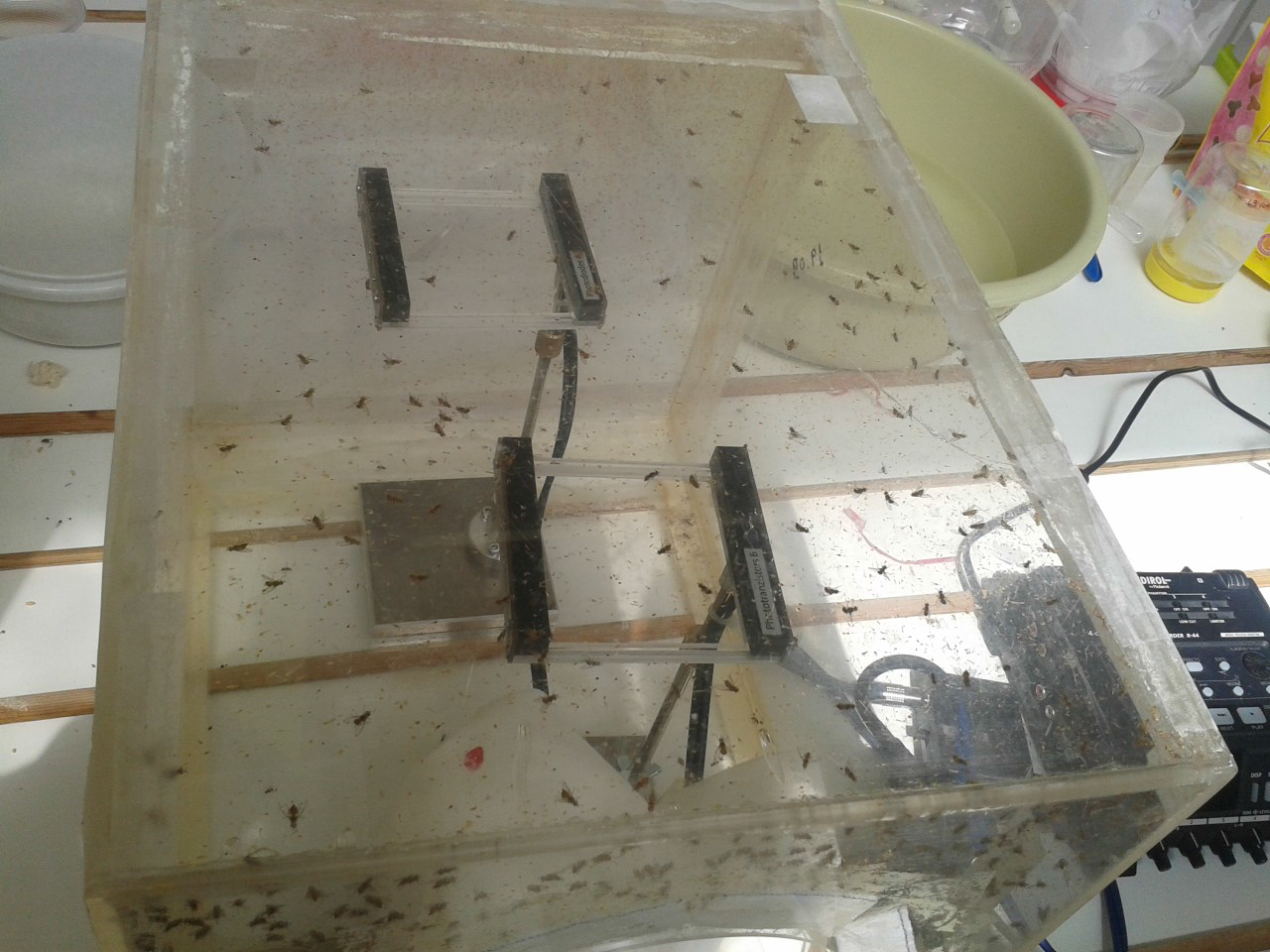

Supplement: S6 Fig — (TIF) [file pone.0140474.s006.tif]

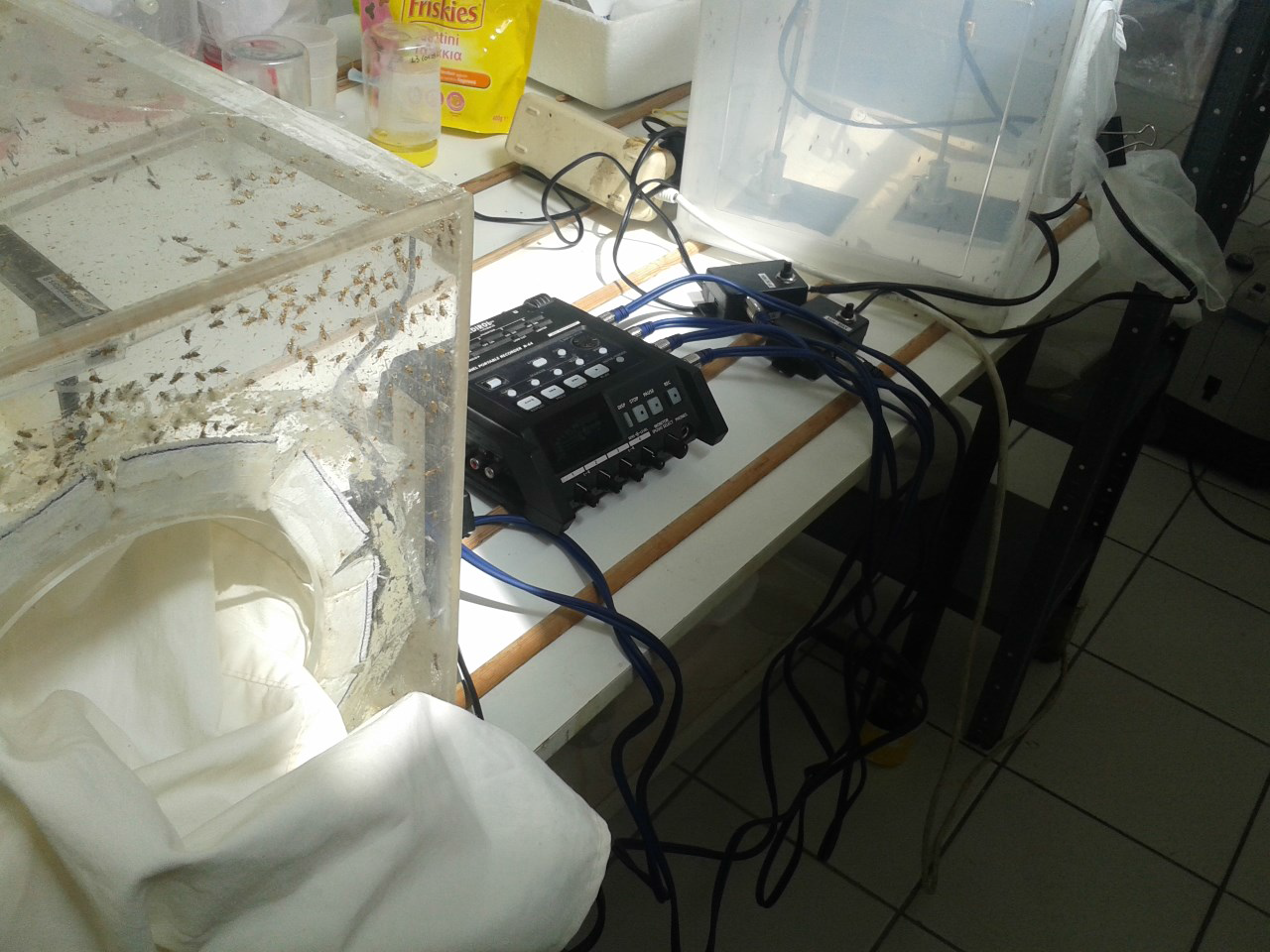

Supplement: S7 Fig — (TIF) [file pone.0140474.s007.tif]

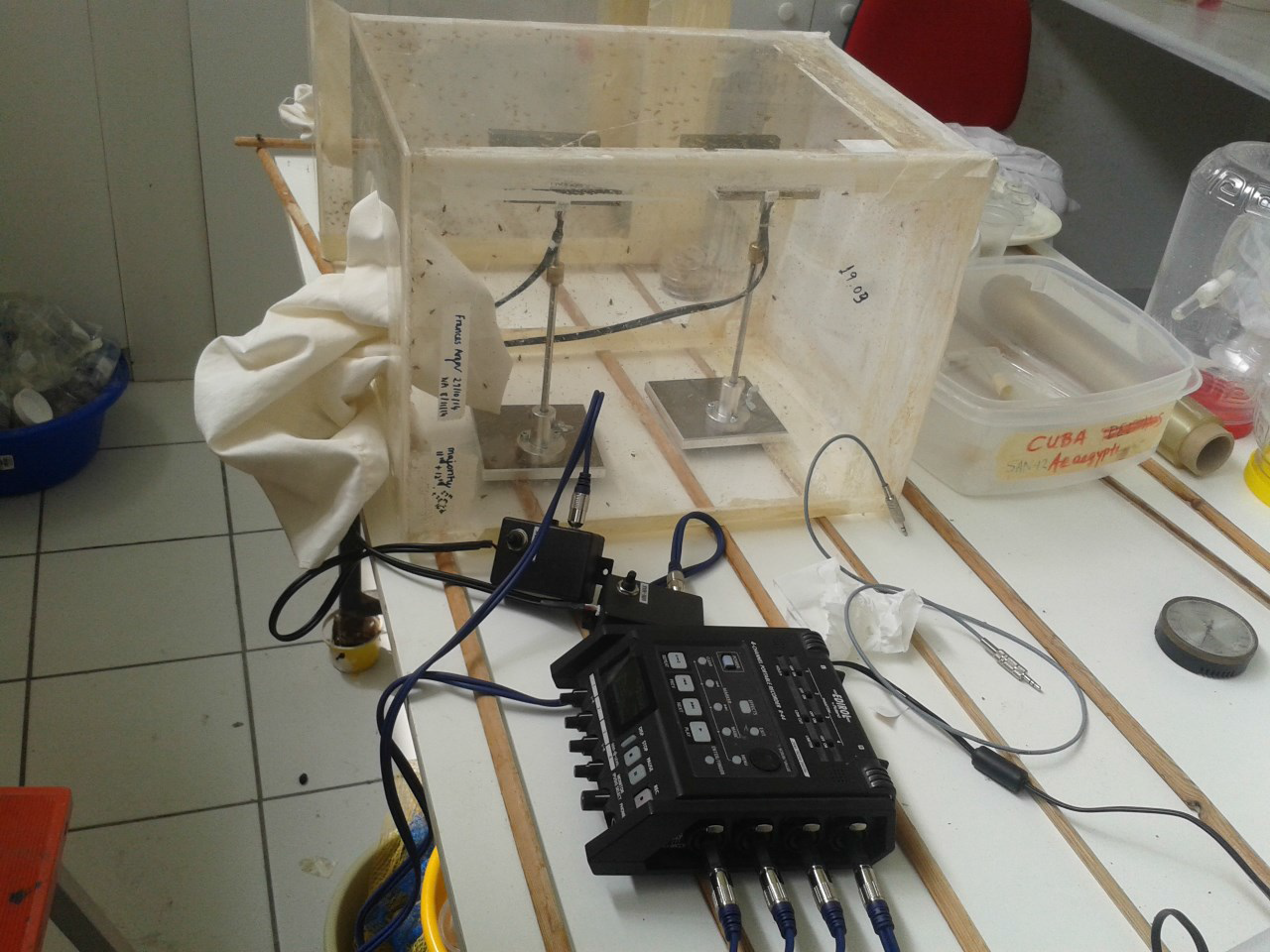

Supplement: S8 Fig — (TIF) [file pone.0140474.s008.tif]

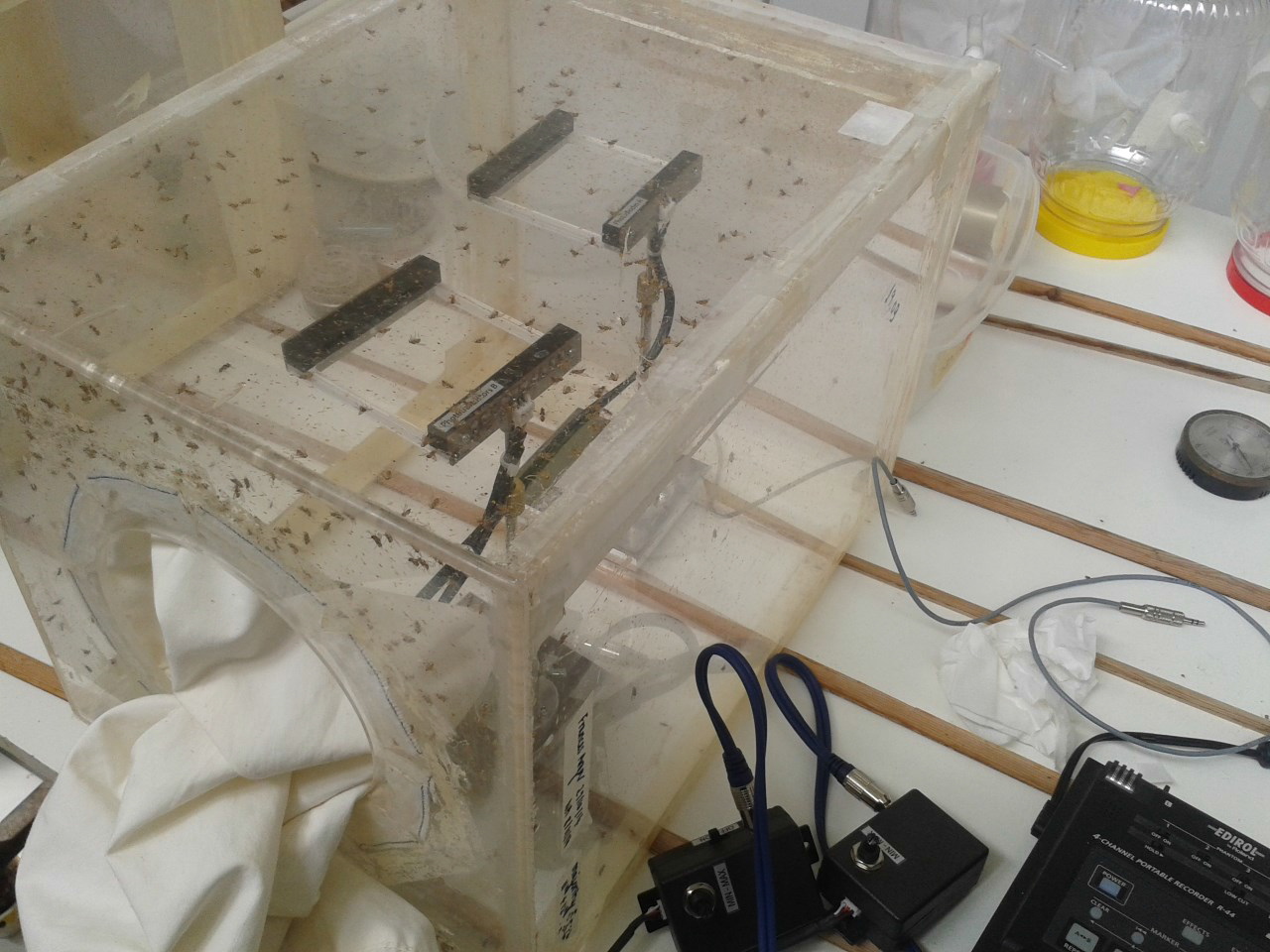

Supplement: S9 Fig — (TIF) [file pone.0140474.s009.tif]

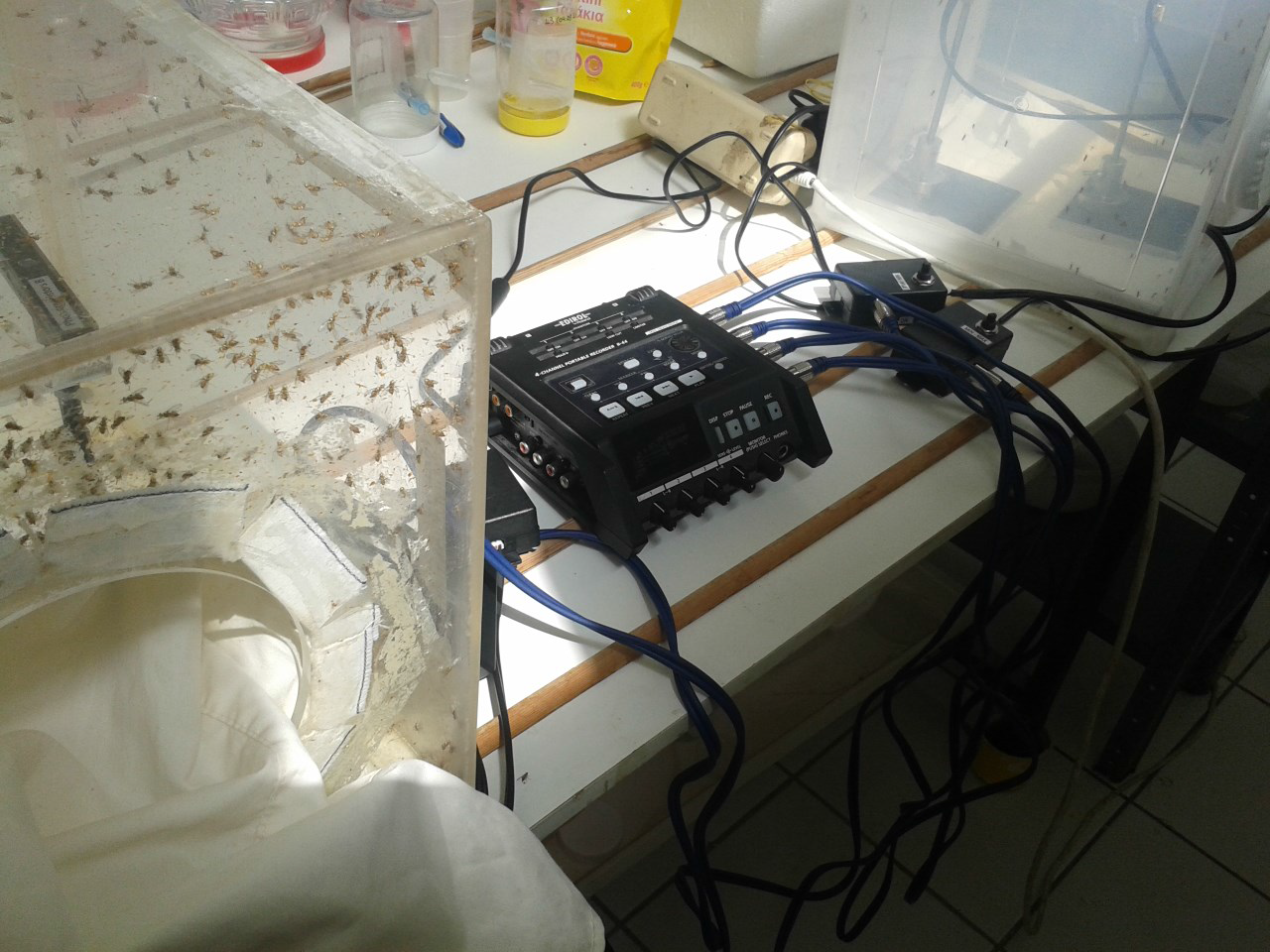

Supplement: S10 Fig — (TIF) [file pone.0140474.s010.tif]

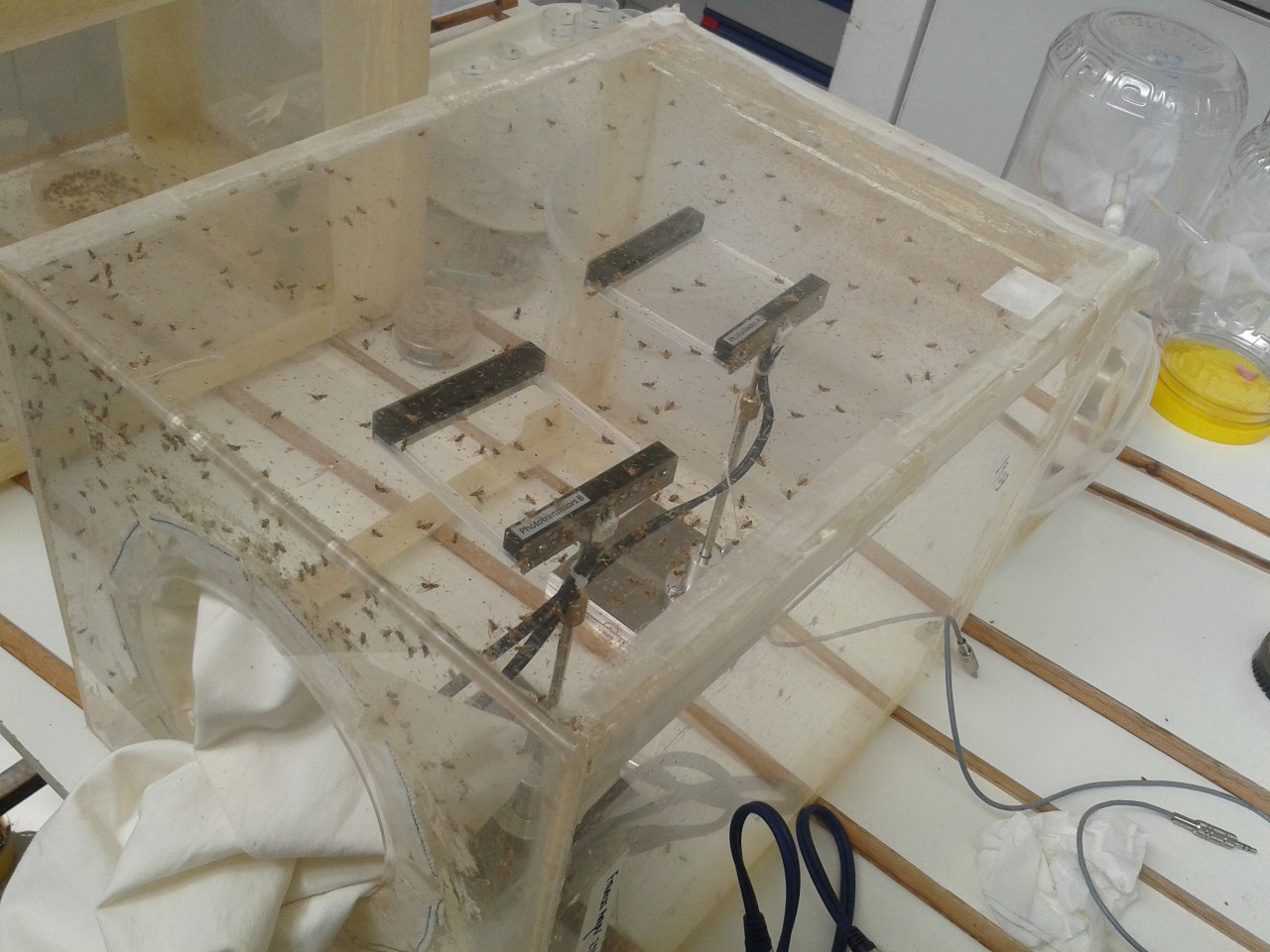

Supplement: S11 Fig — (TIF) [file pone.0140474.s011.tif]

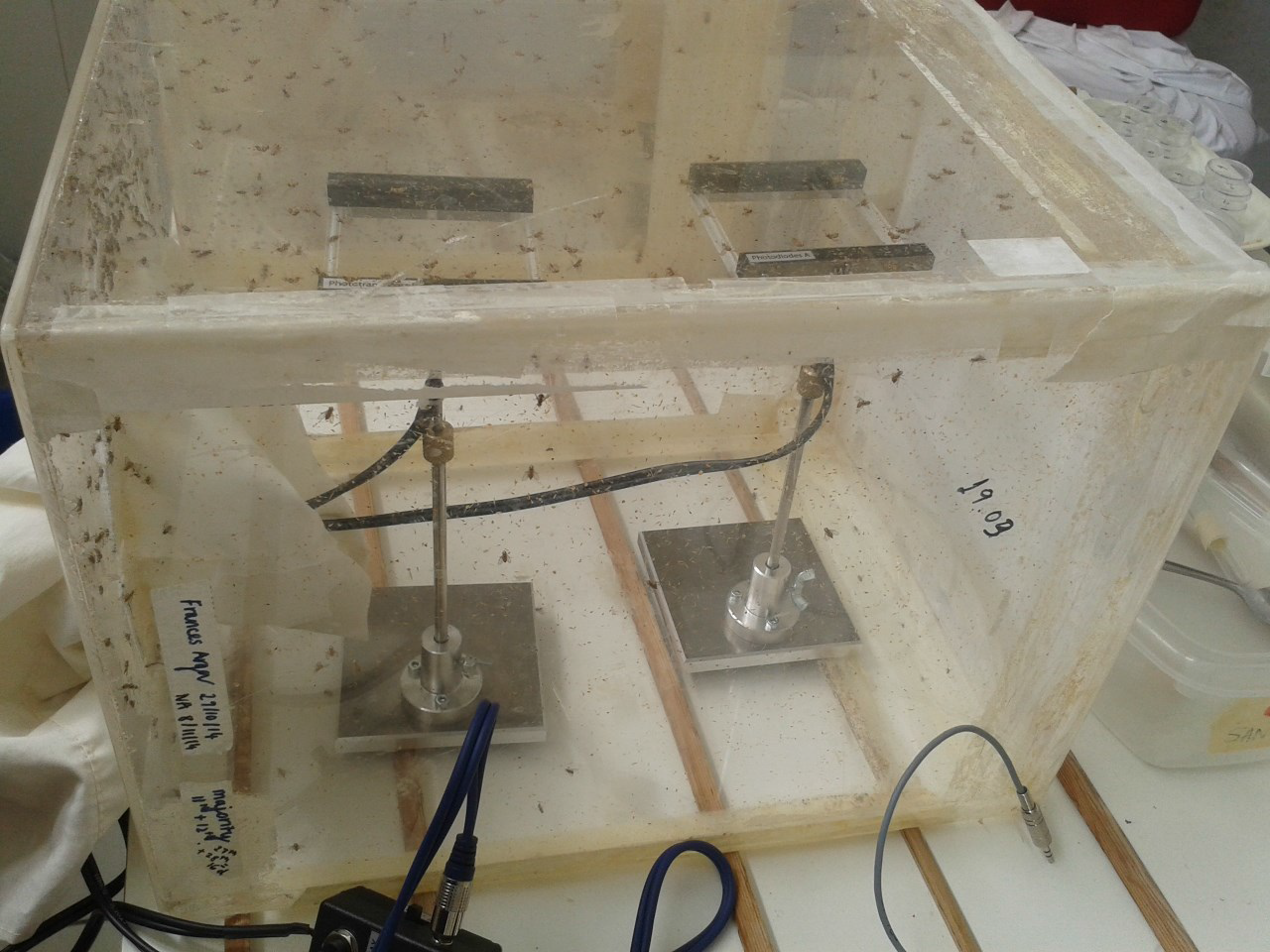

Supplement: S12 Fig — (TIF) [file pone.0140474.s012.tif]

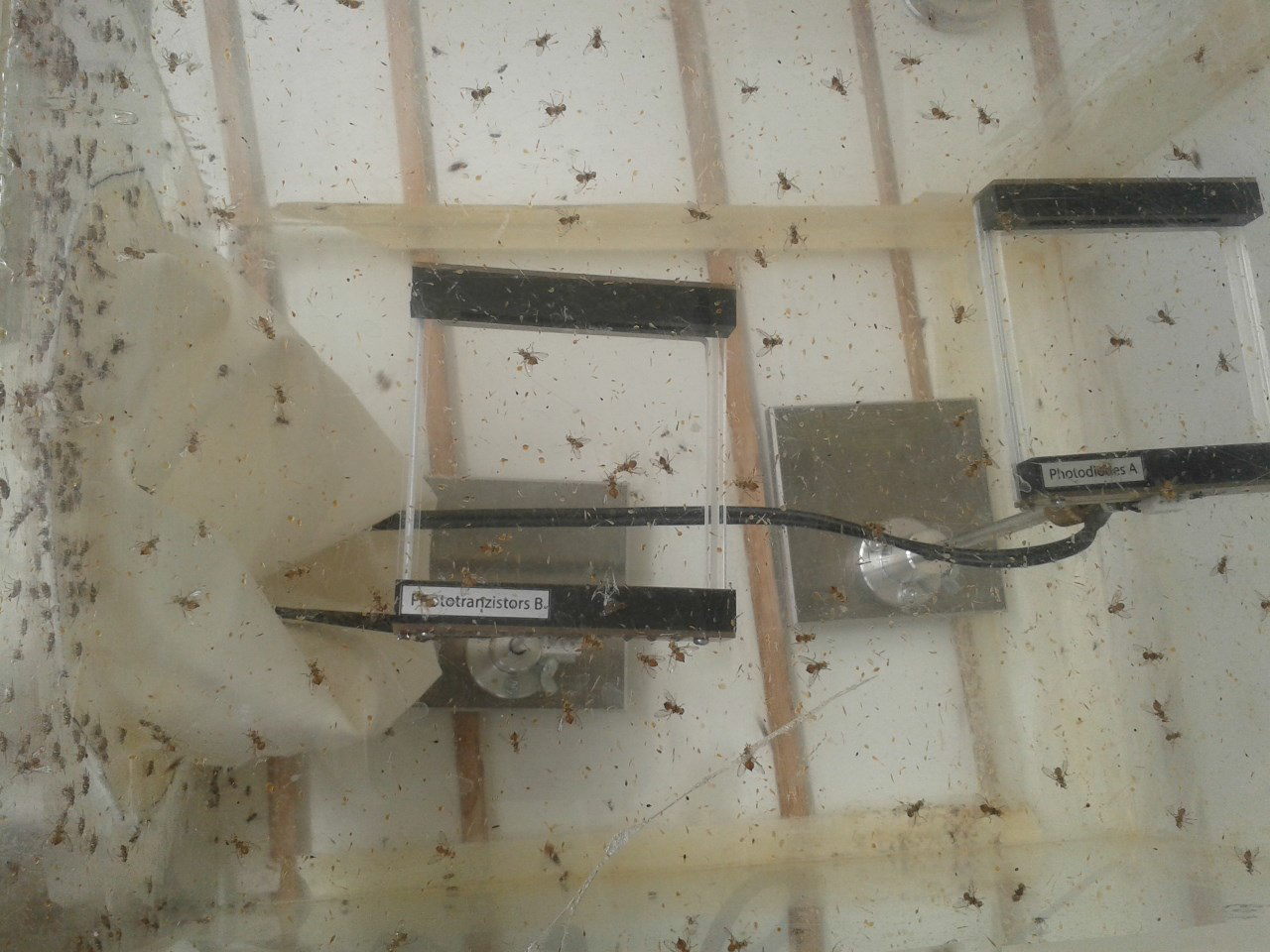

Supplement: S13 Fig — (TIF) [file pone.0140474.s013.tif]

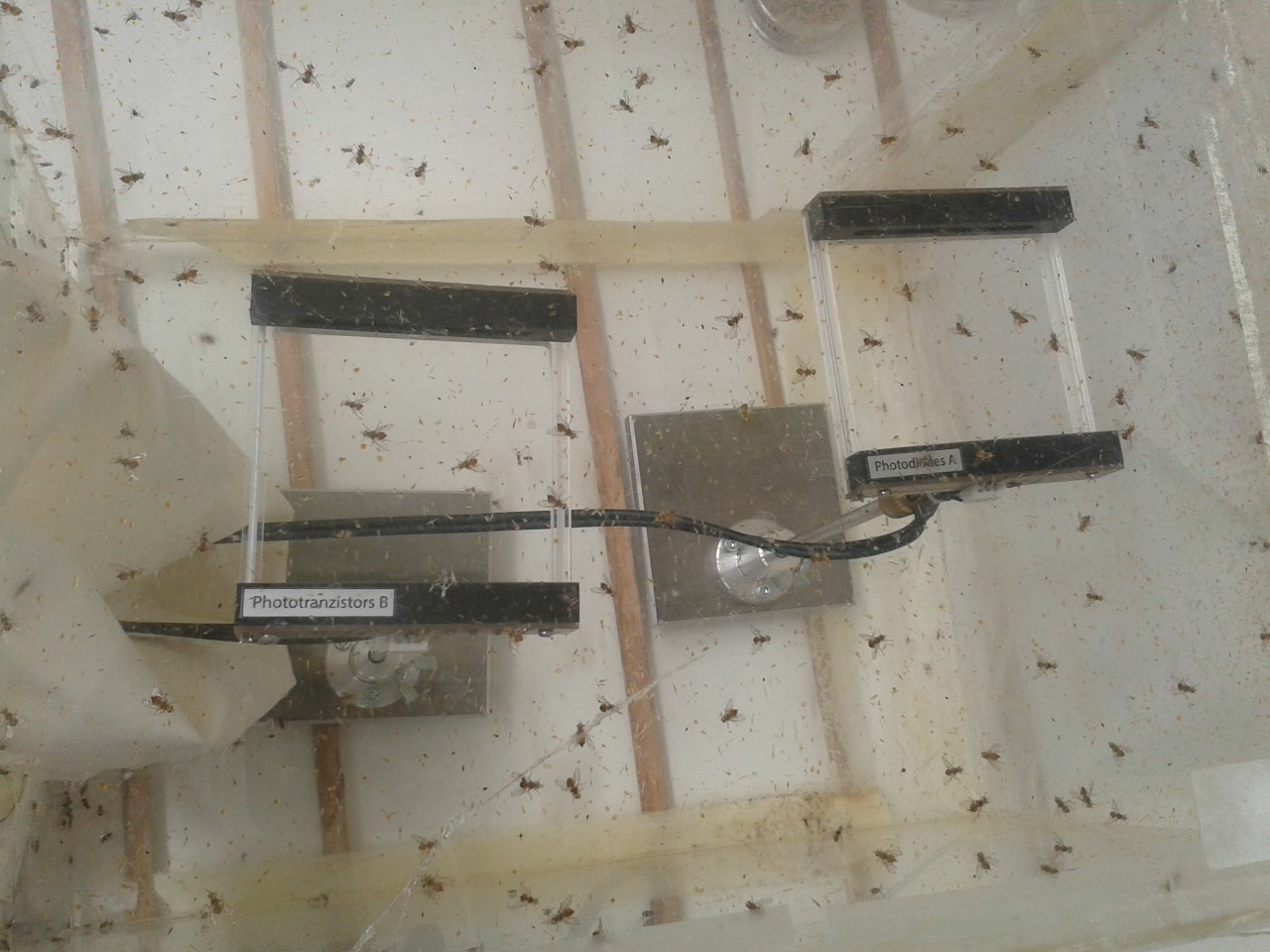

Supplement: S14 Fig — (TIF) [file pone.0140474.s014.tif]

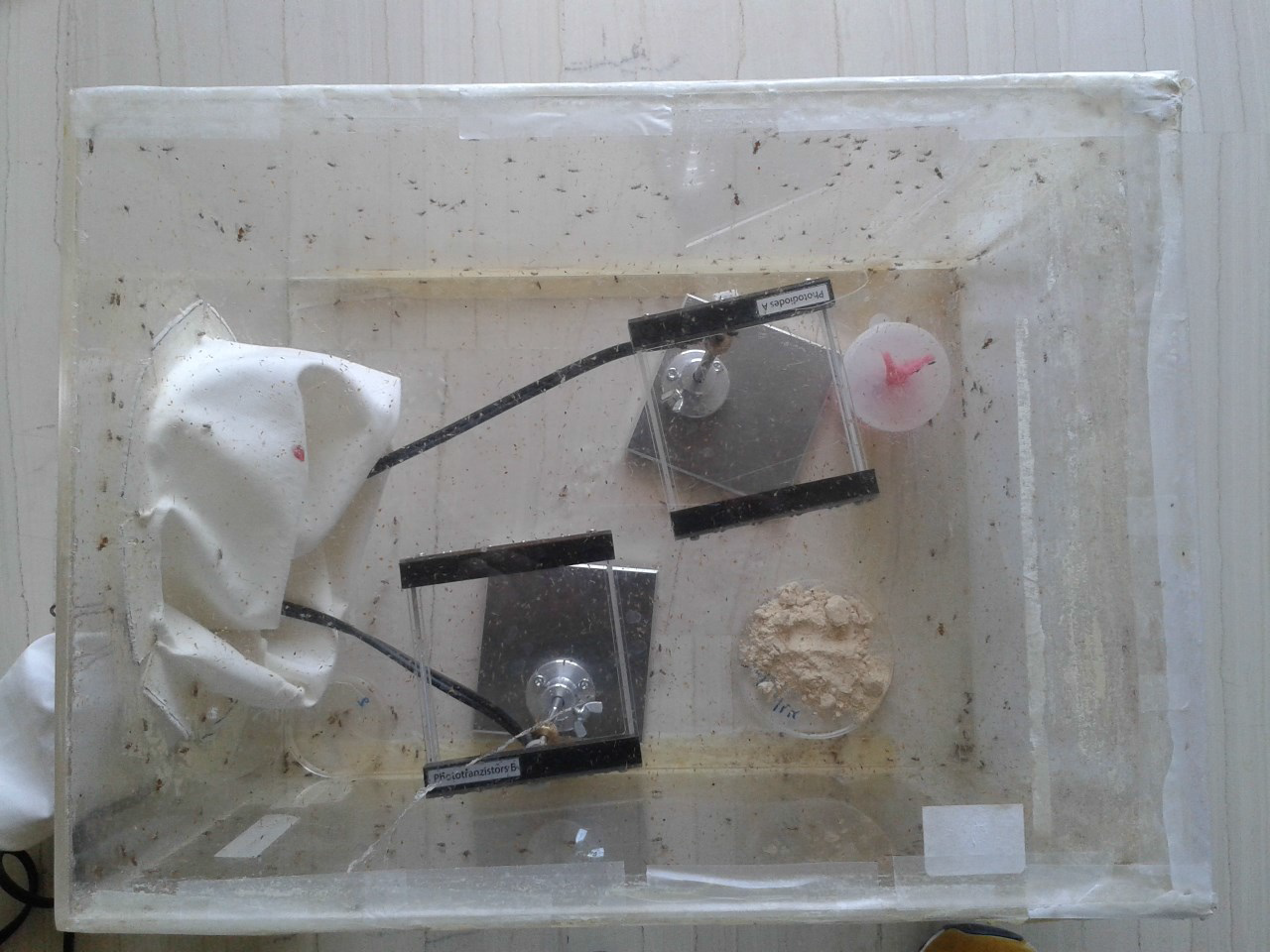

Supplement: S15 Fig — (TIF) [file pone.0140474.s015.tif]

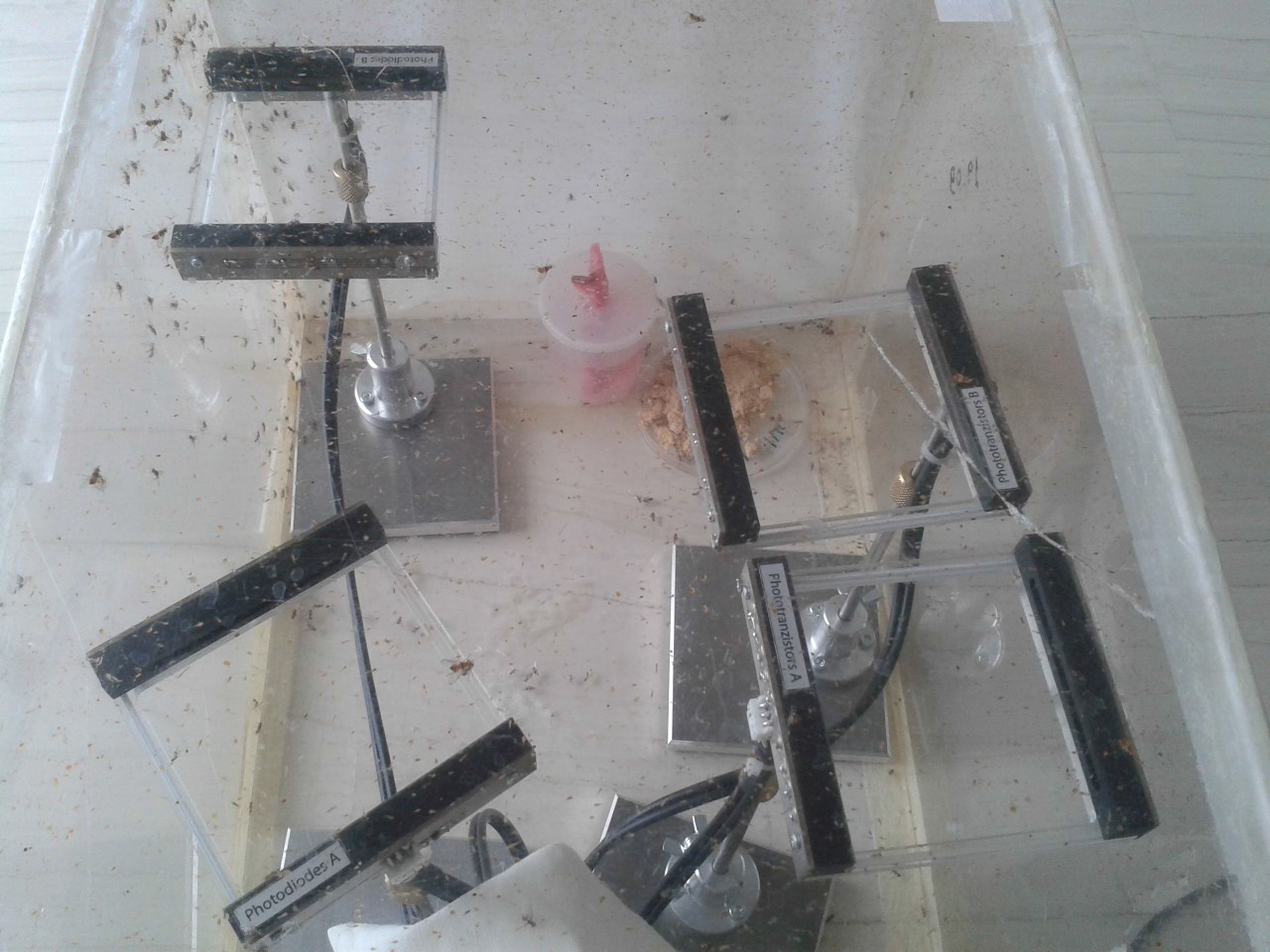

Supplement: S16 Fig — (TIF) [file pone.0140474.s016.tif]

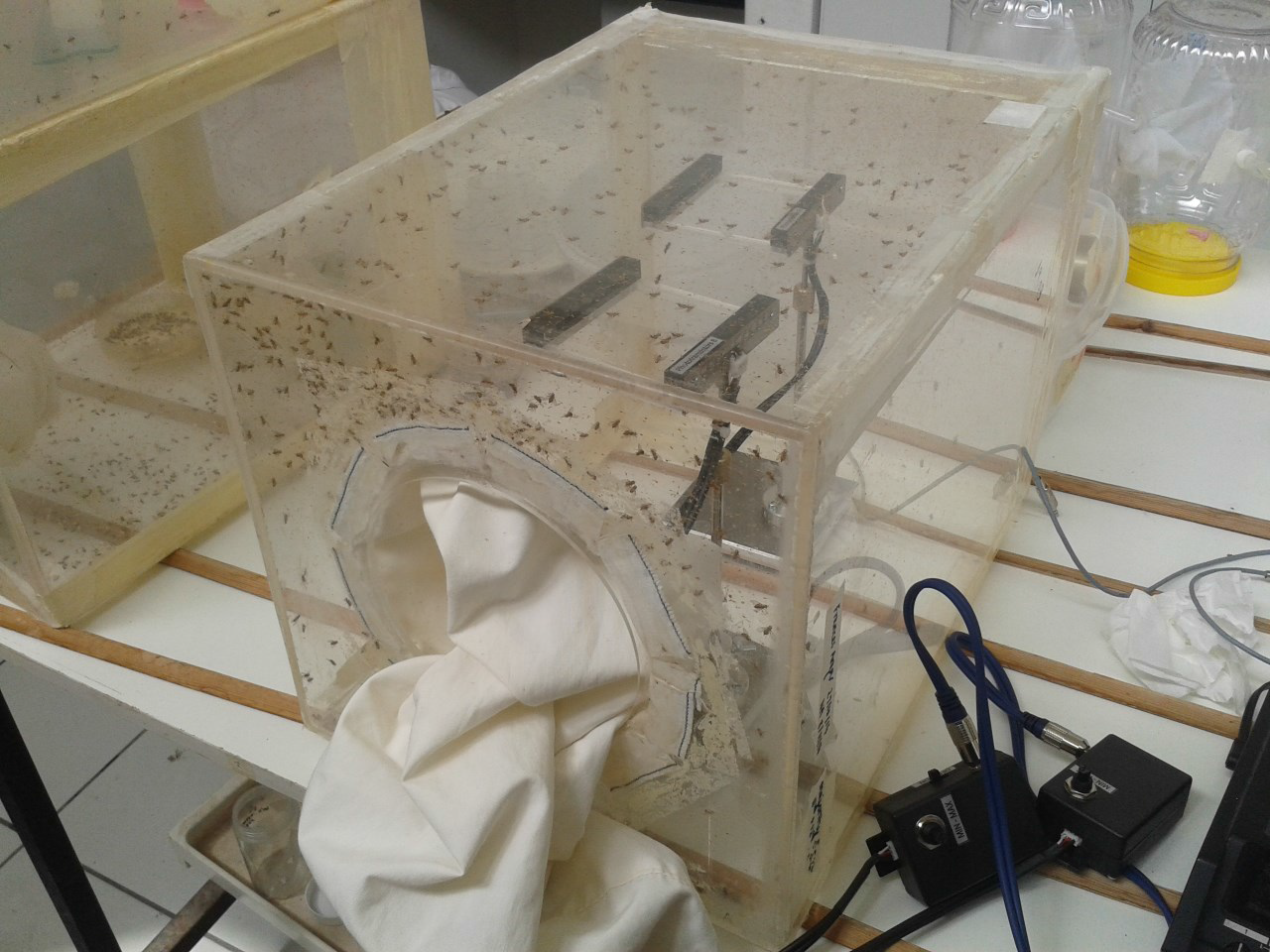

Supplement: S17 Fig — (TIF) [file pone.0140474.s017.tif]

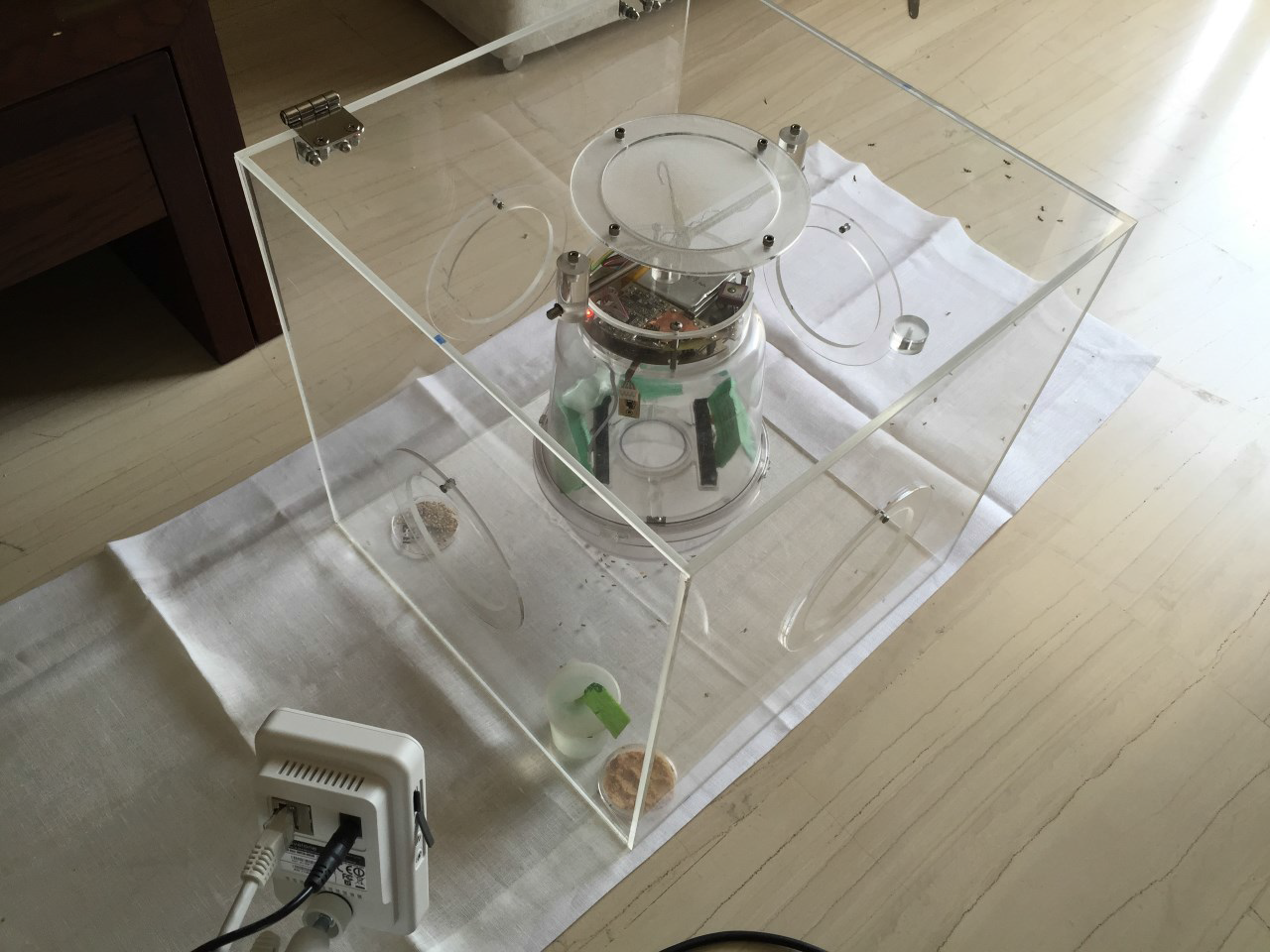

Supplement: S18 Fig — (TIF) [file pone.0140474.s018.tif]

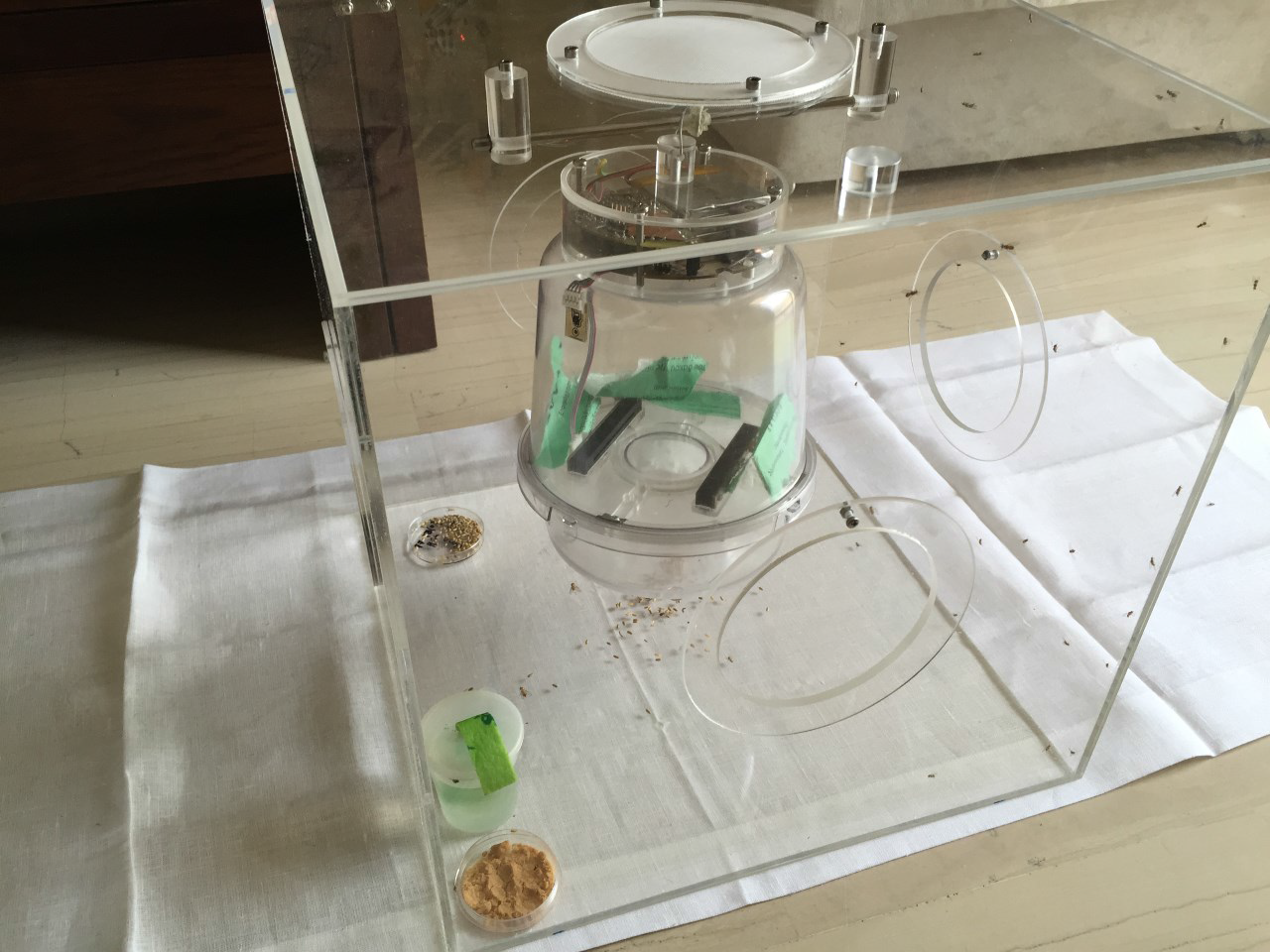

Supplement: S19 Fig — (TIF) [file pone.0140474.s019.tif]

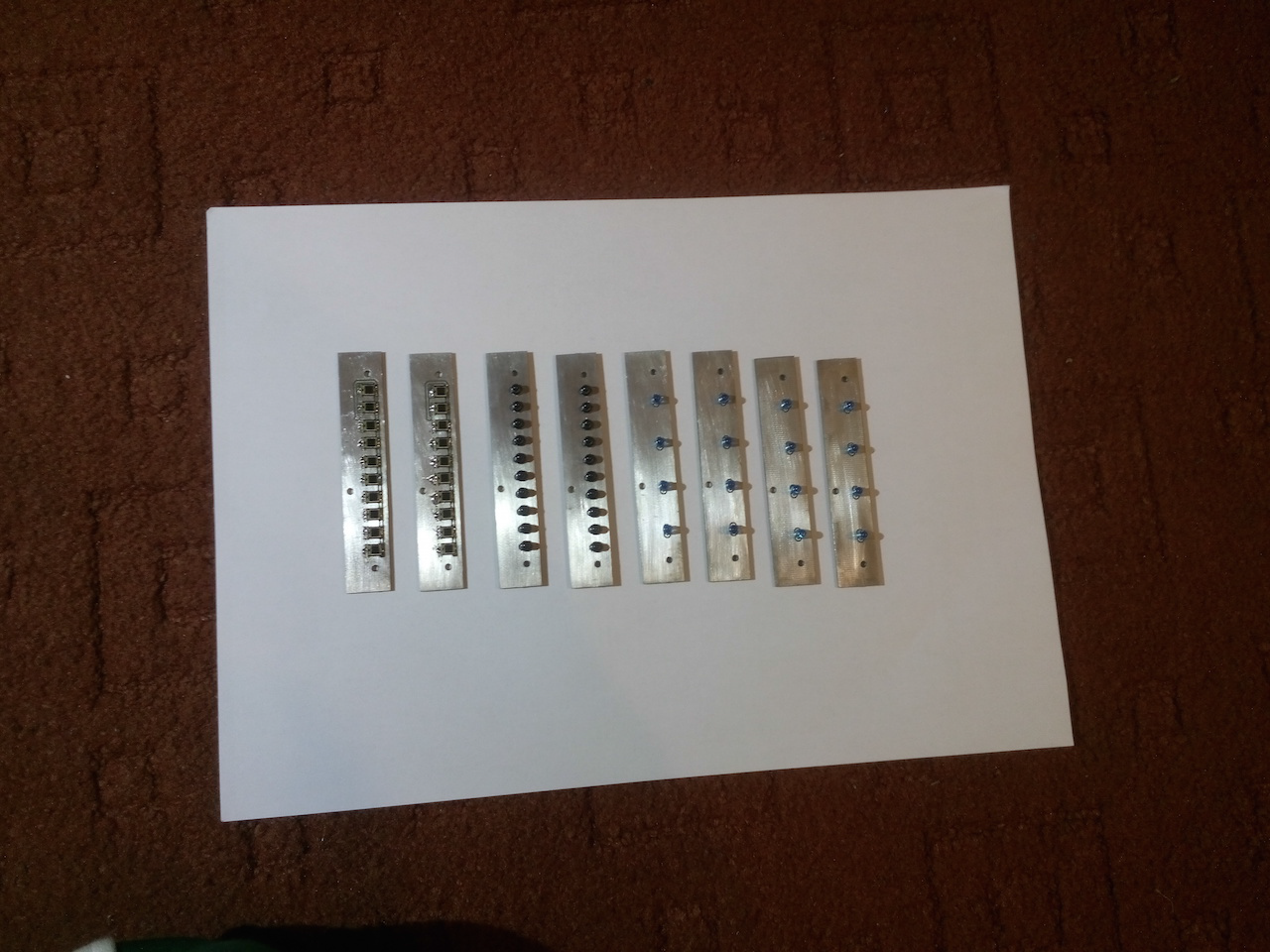

Supplement: S20 Fig — (TIF) [file pone.0140474.s020.tif]

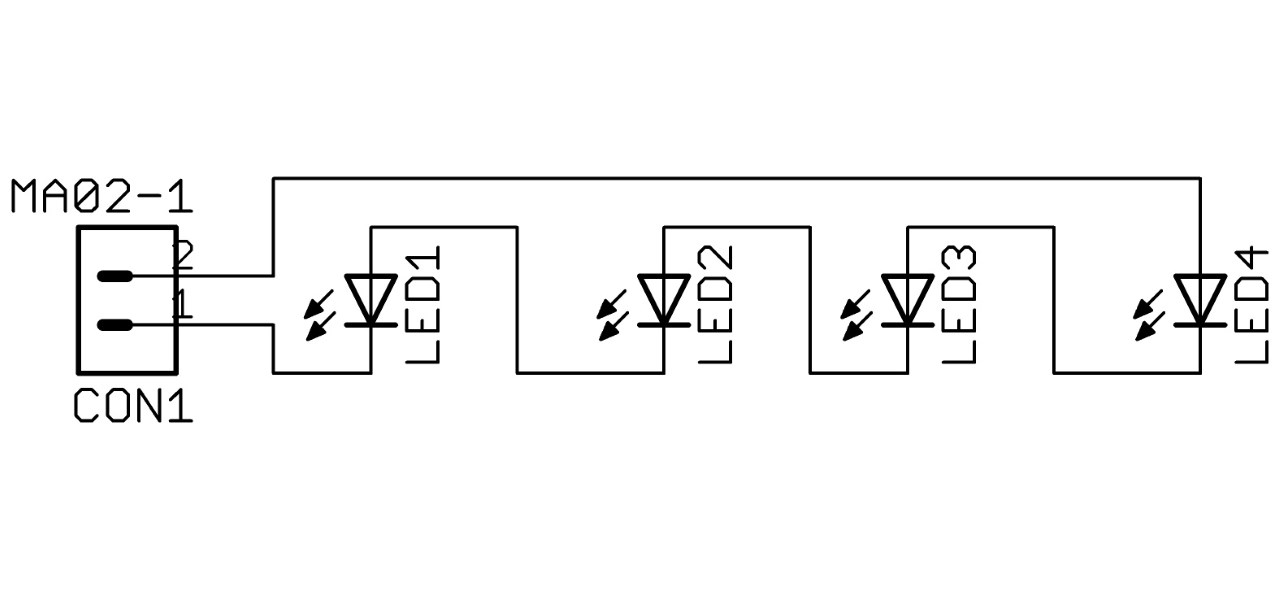

Supplement: S21 Fig — Light is provided by 4 infrared LEDs (940nm) connected in row, led by a 2.7 mA current. (TIF) [file pone.0140474.s021.tif]

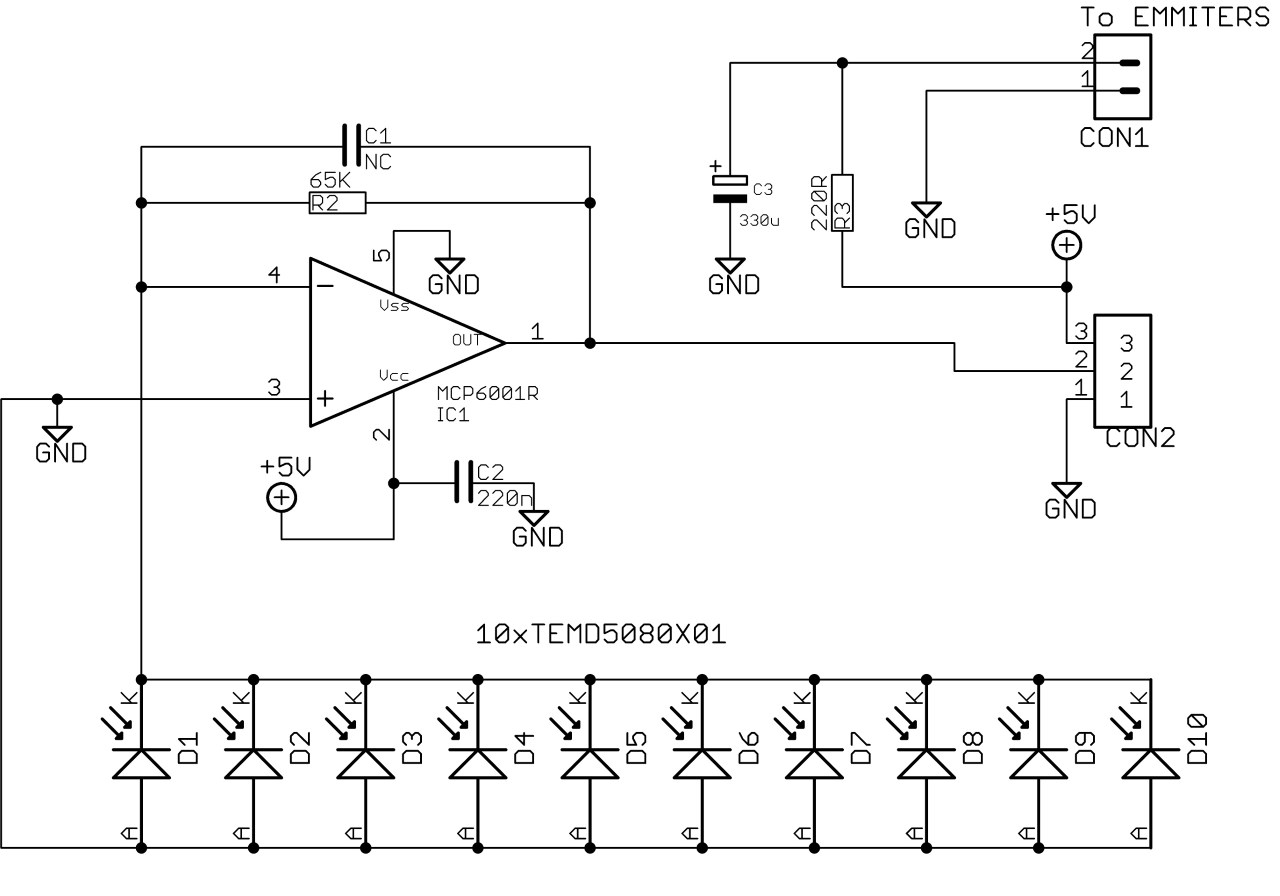

Supplement: S22 Fig — The receiver is a linear array of 10 photodiodes connected in parallel. The received light is amplified by the IC1 and is driven to the band-pass filter. (TIF) [file pone.0140474.s022.tif]

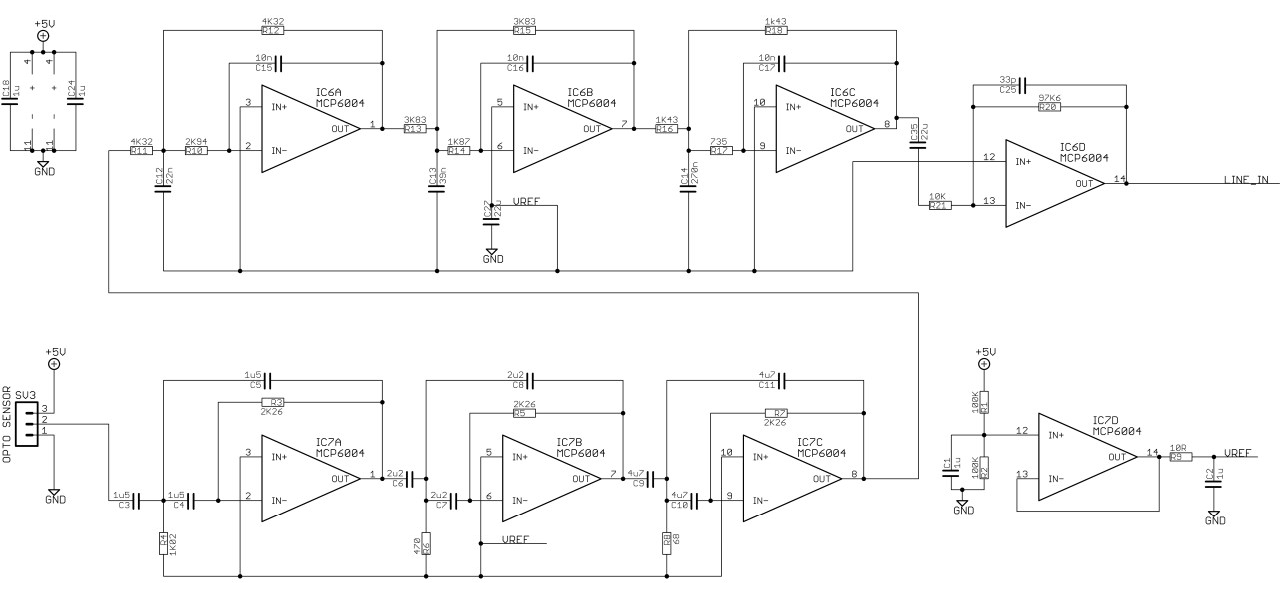

Supplement: S23 Fig — The filtering process is carried out by the high-pass filter IC7 A, B & C and the low-pass filter IC6 A, B & C. Subsequently, the signal is amplified by IC6D and driven to the processor. (TIF) [file pone.0140474.s023.tif]

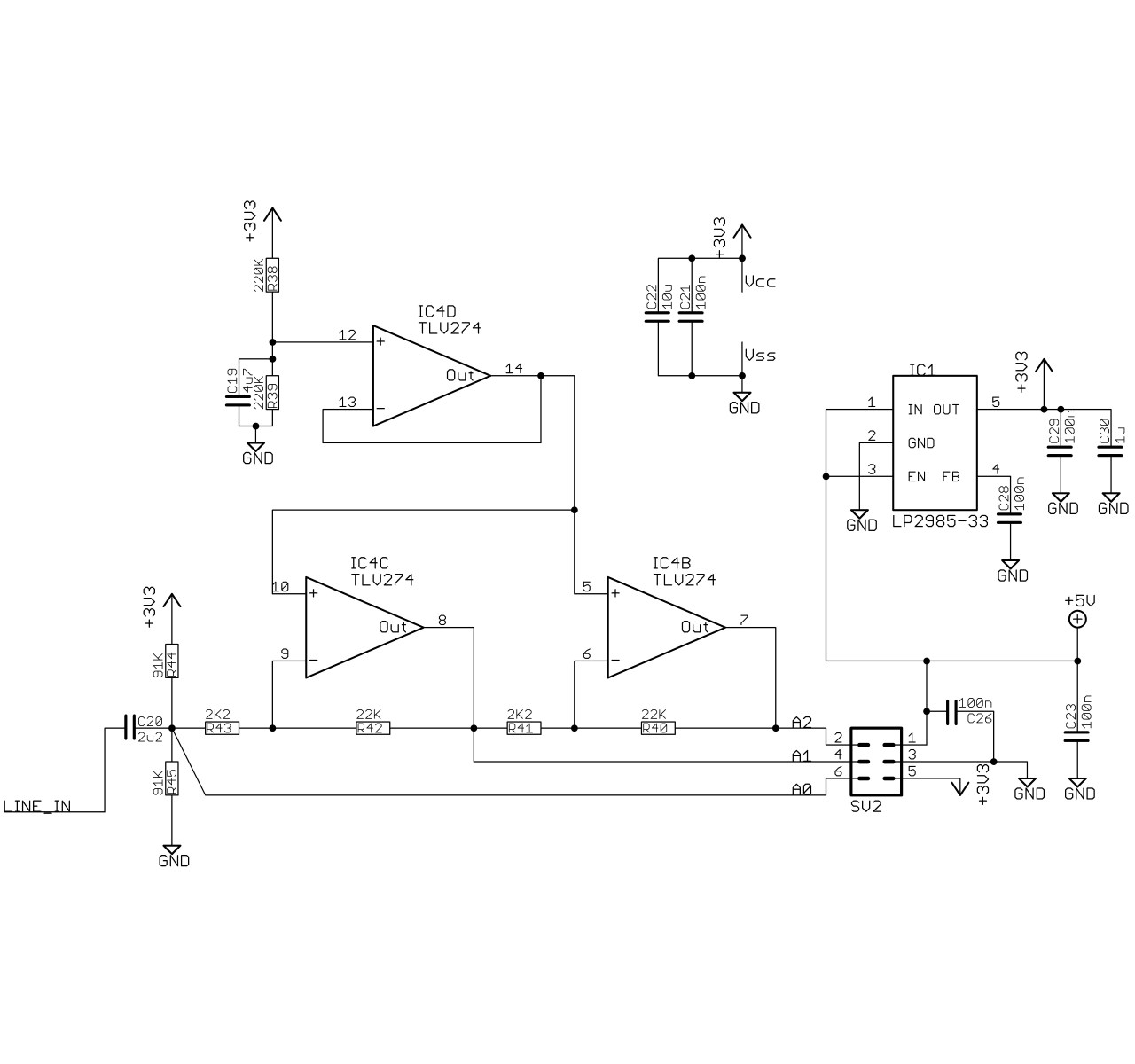

Supplement: S24 Fig — The signal coming out of the filters is amplified by the current circuit by the factors 1, 10 and 100. The signals x1, x10 and x100 are driven to the 3 analog inputs of the microcontroller. (TIF) [file pone.0140474.s024.tif]

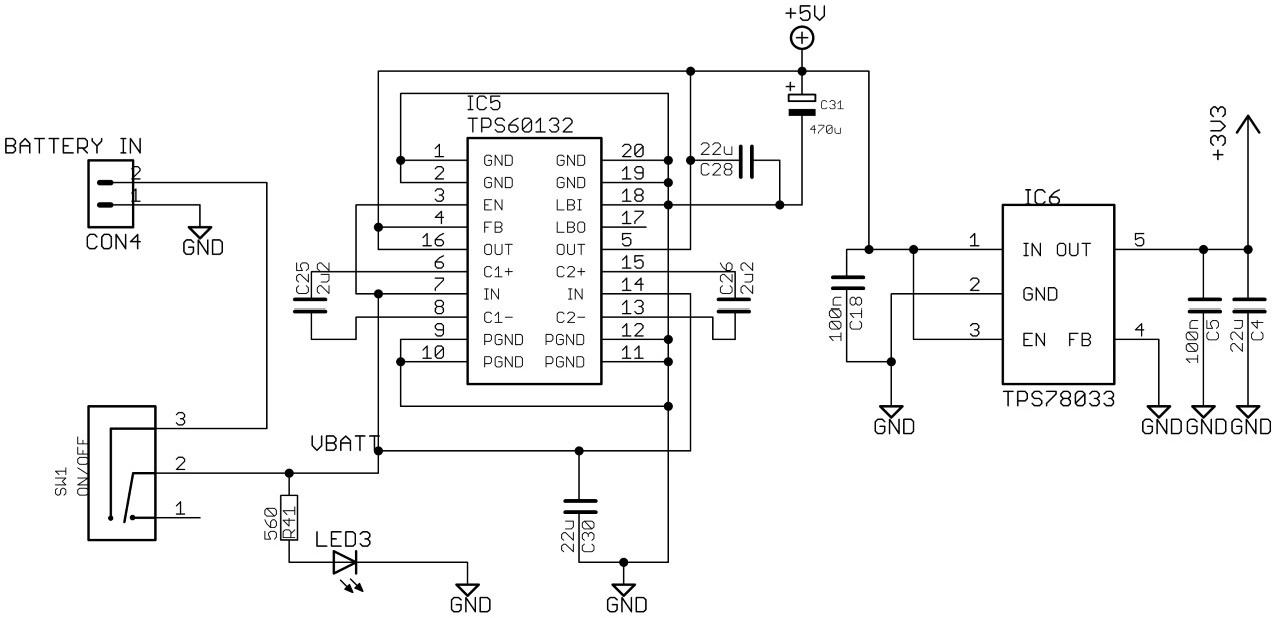

Supplement: S25 Fig — The power supply circuit has as input the Lithium battery 3.7 Vdc and supplies voltages 3.3 Vdc & 5 Vdc for the digital and analogue circuits. (TIF) [file pone.0140474.s025.tif]

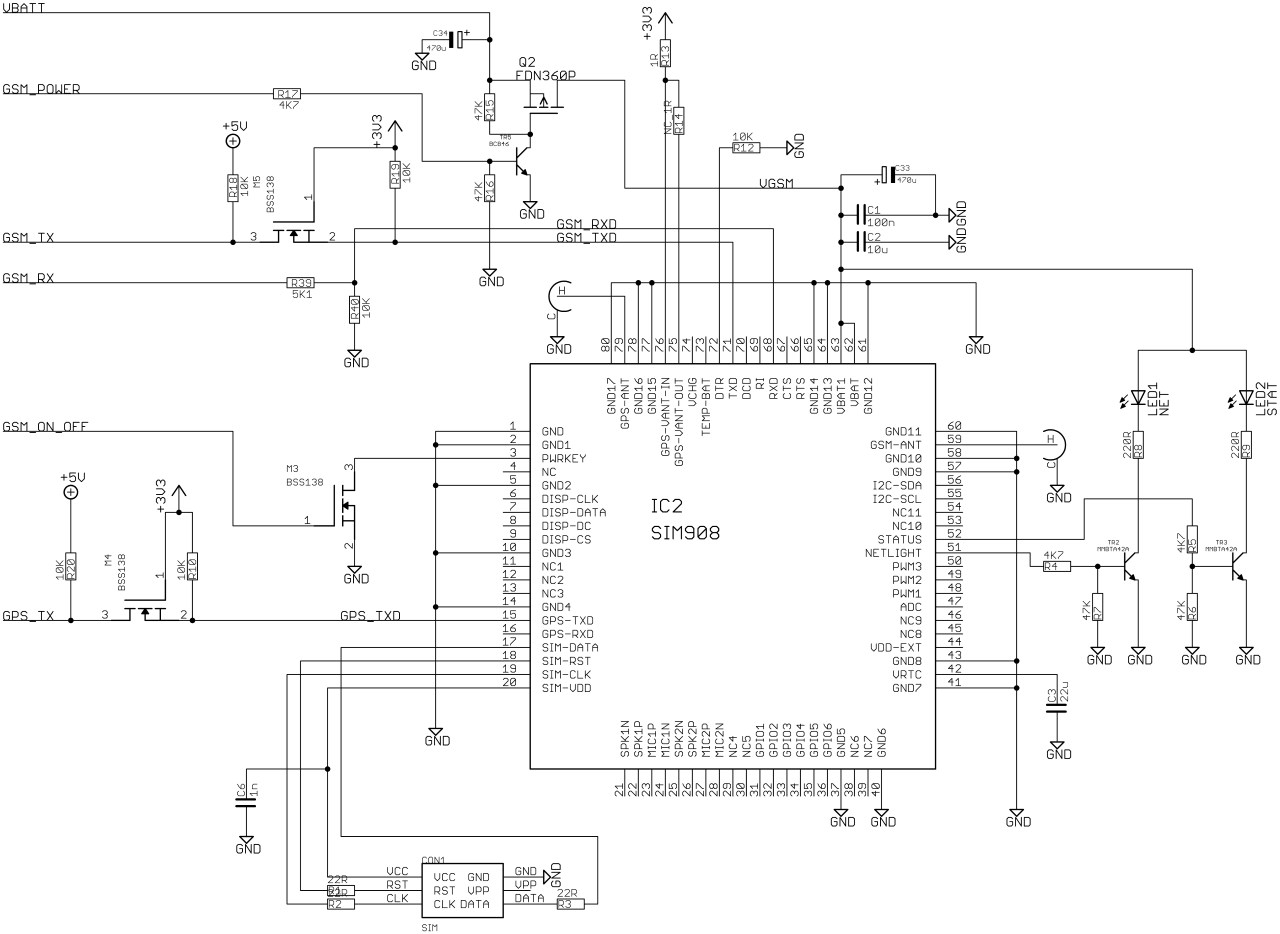

Supplement: S26 Fig — The GSM module sends text data through GPRS. It also embeds a GSM. When not in use MOSFET Q2 cuts its power supply so it does not consume energy. It is controlled via the Microcontroller (ATMEGA2560). (TIF) [file pone.0140474.s026.tif]

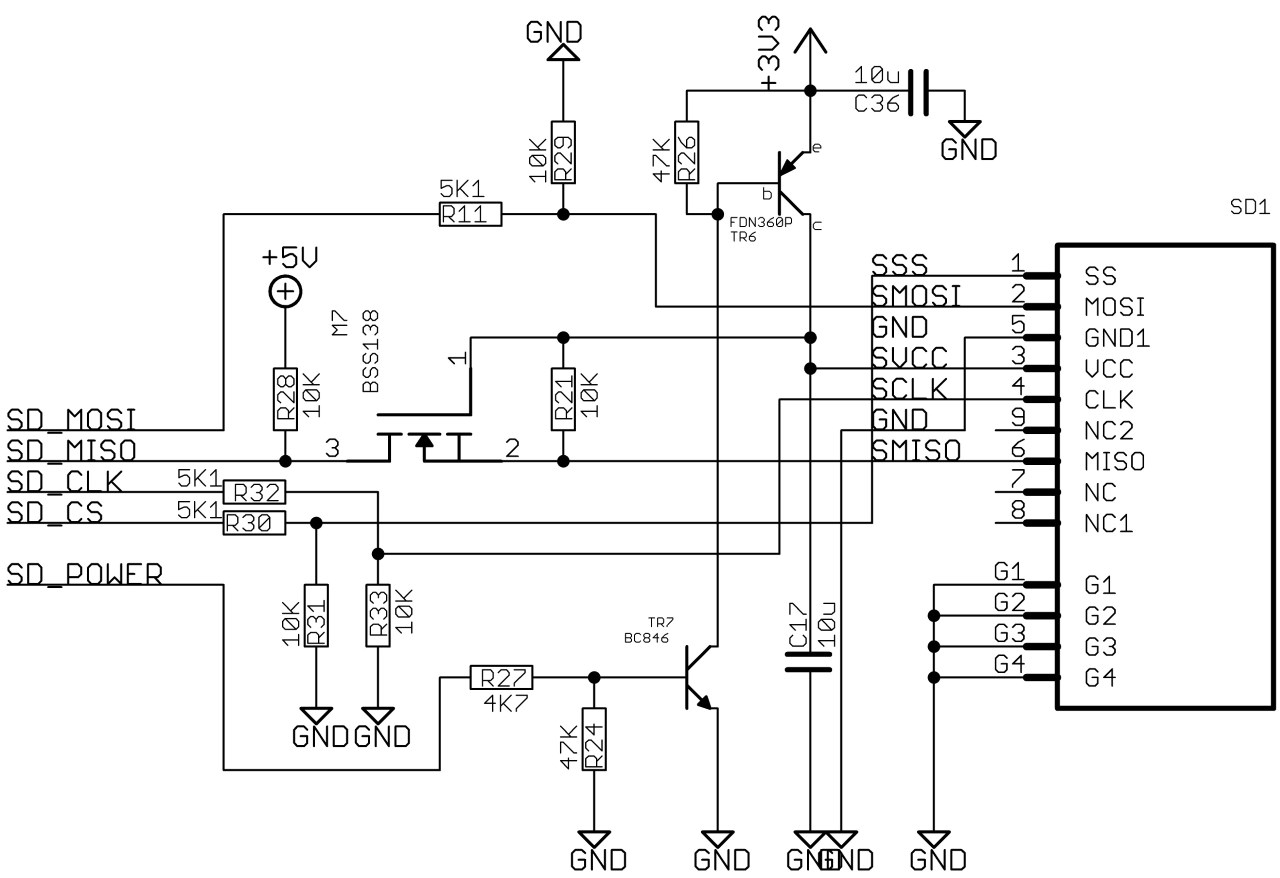

Supplement: S27 Fig — In the SD card the microcontroller stores the recordings (we store the FFT but the actual recording of the ADC can be also stored) for further analysis. When not in use MOSFET M7 cuts its power supply so it does not consume energy. (TIF) [file pone.0140474.s027.tif]

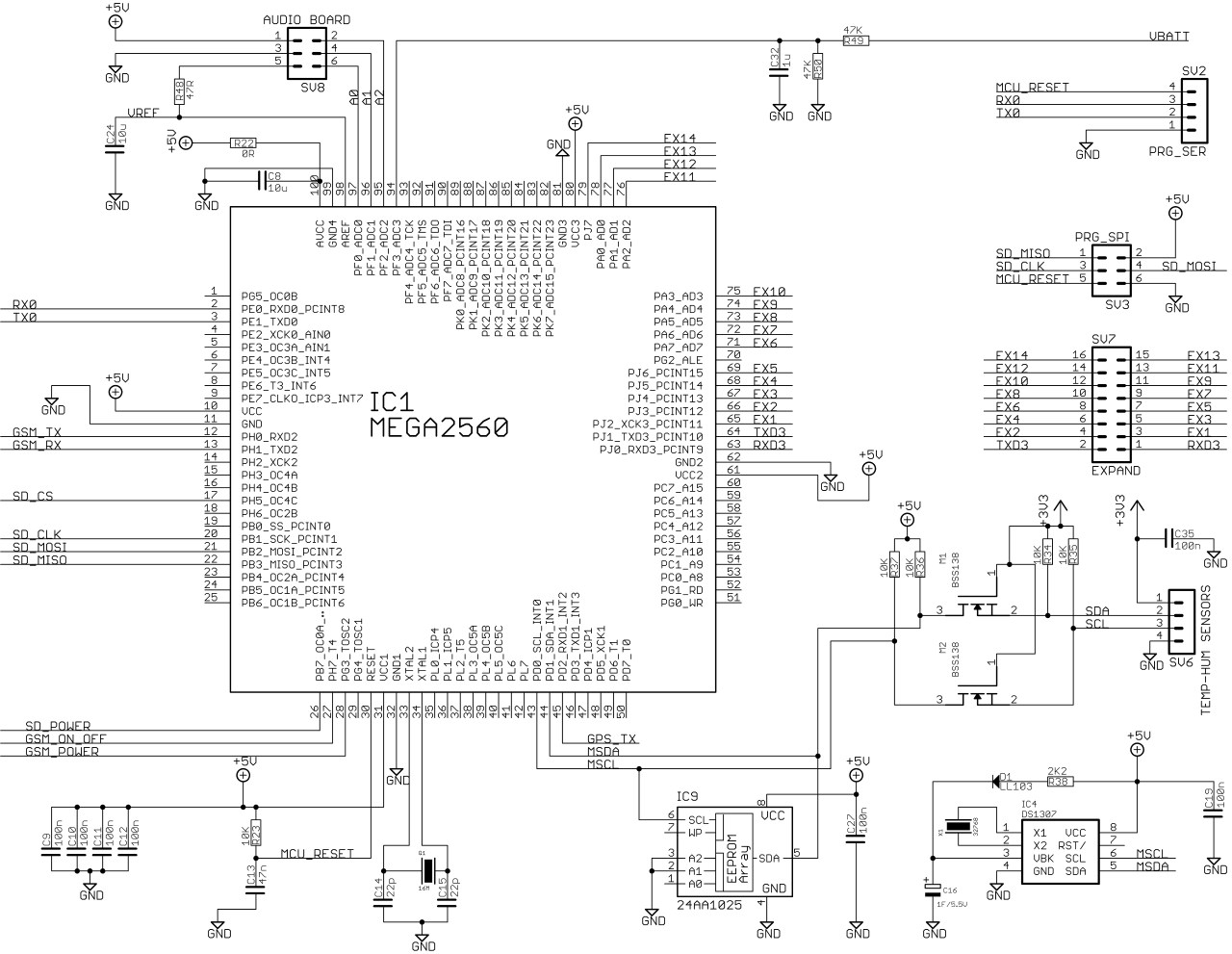

Supplement: S28 Fig — Processor IC1, receives the analog signal from the inputs A0, A1 and A2 and selects the proper gain. IC1 is processing the resulting digital signal. It also controls the GSM module, the humidity and temperature sensors. (TIF) [file pone.0140474.s028.tif]

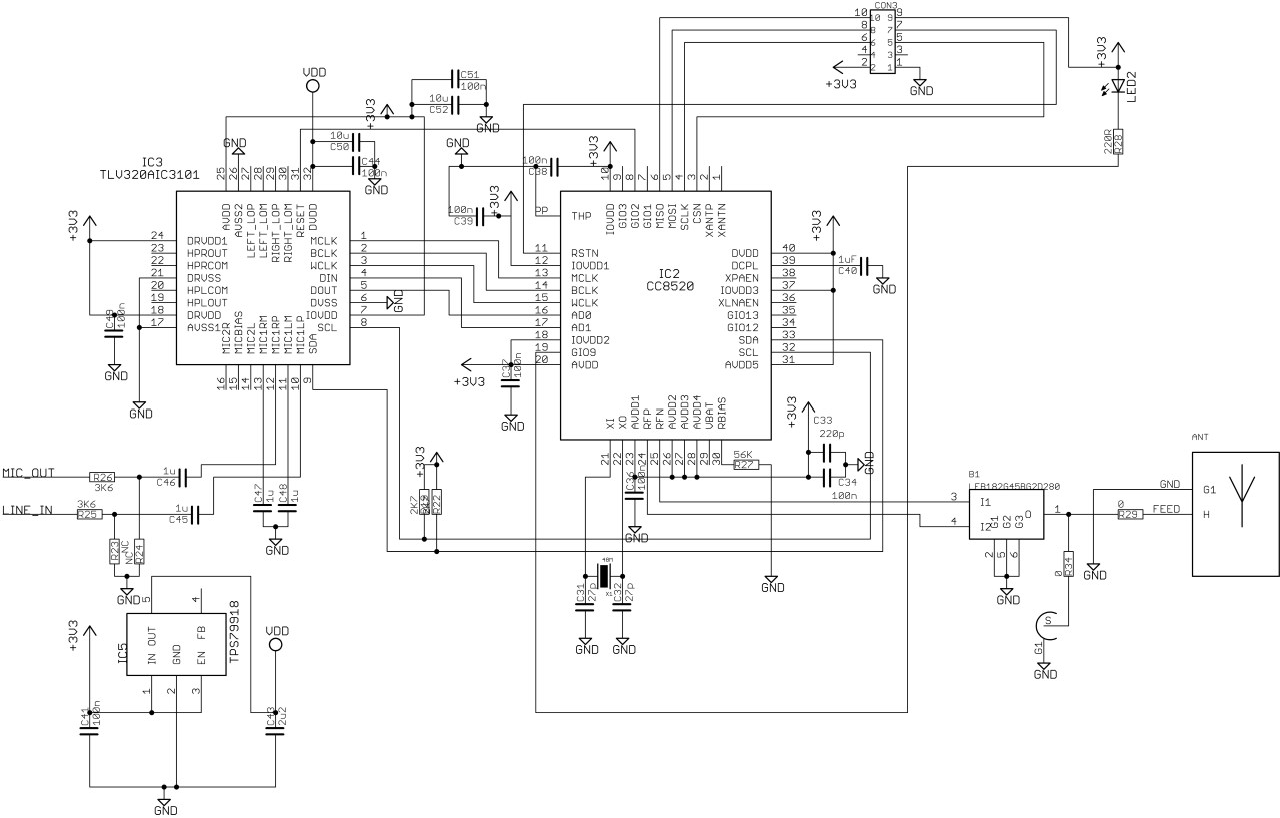

Supplement: S29 Fig — Wireless transmission of the recording of the optoelectronic sensor prior to its entrance to the microcontroller. (TIF) [file pone.0140474.s029.tif]

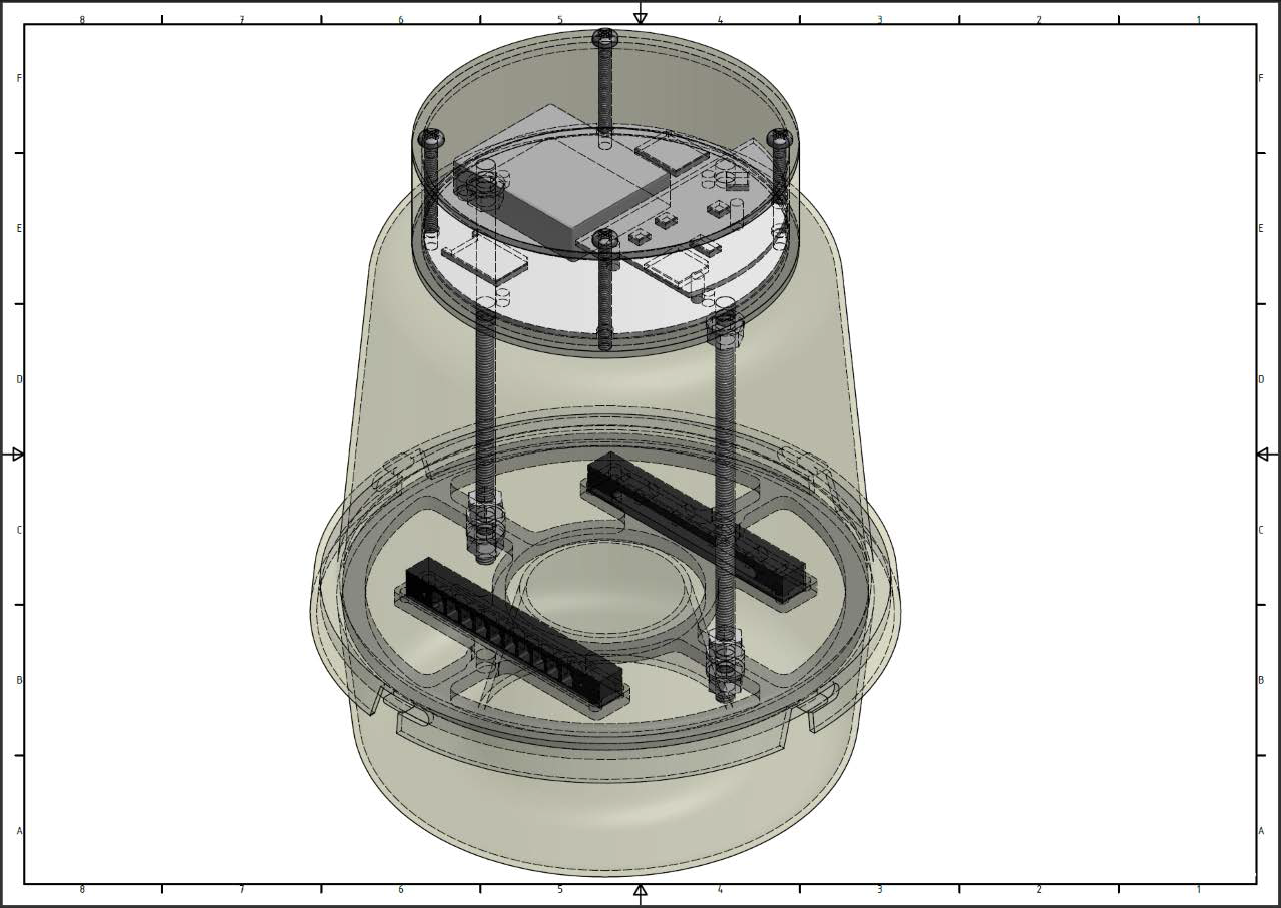

Supplement: S30 Fig — (TIF) [file pone.0140474.s030.tif]

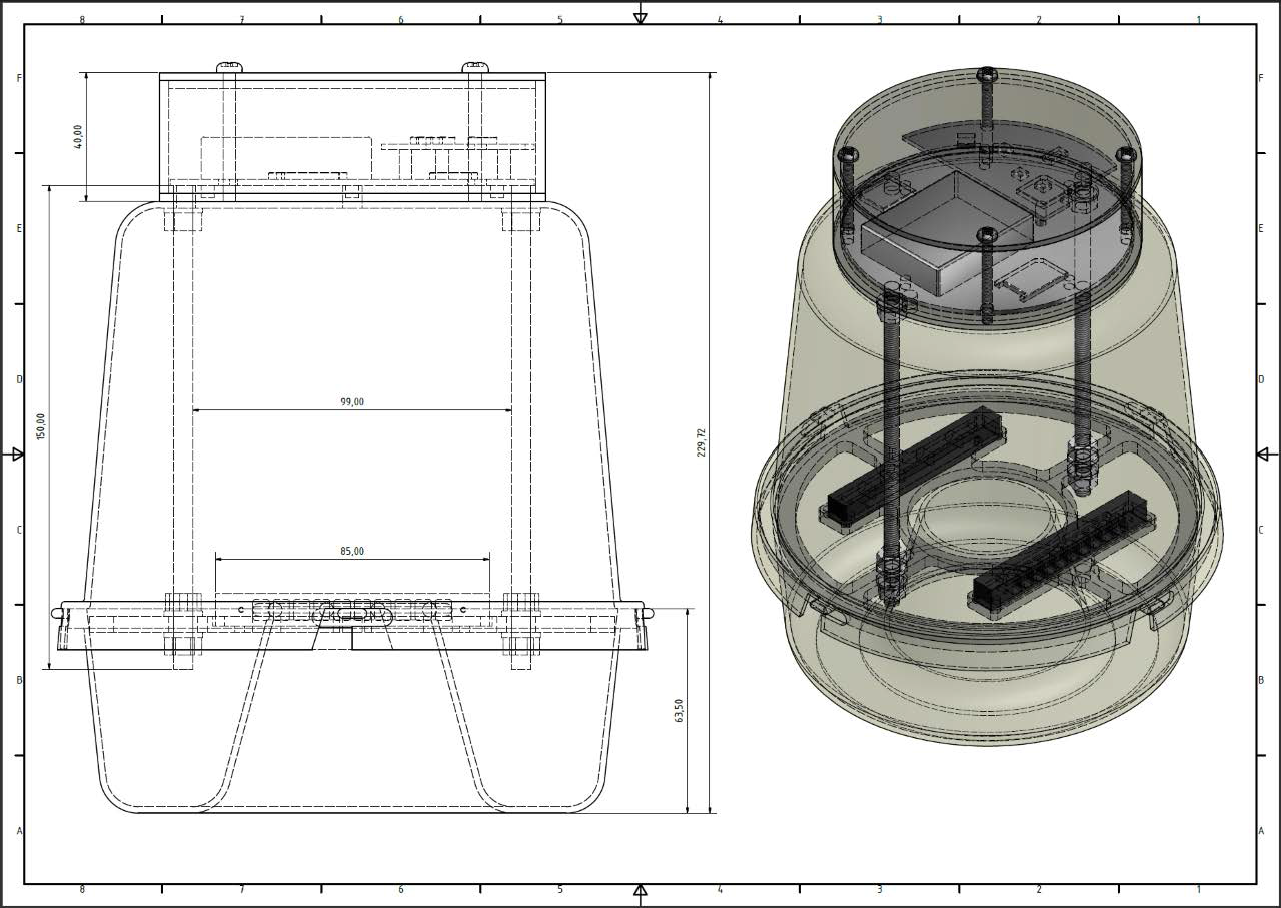

Supplement: S31 Fig — (TIF) [file pone.0140474.s031.tif]

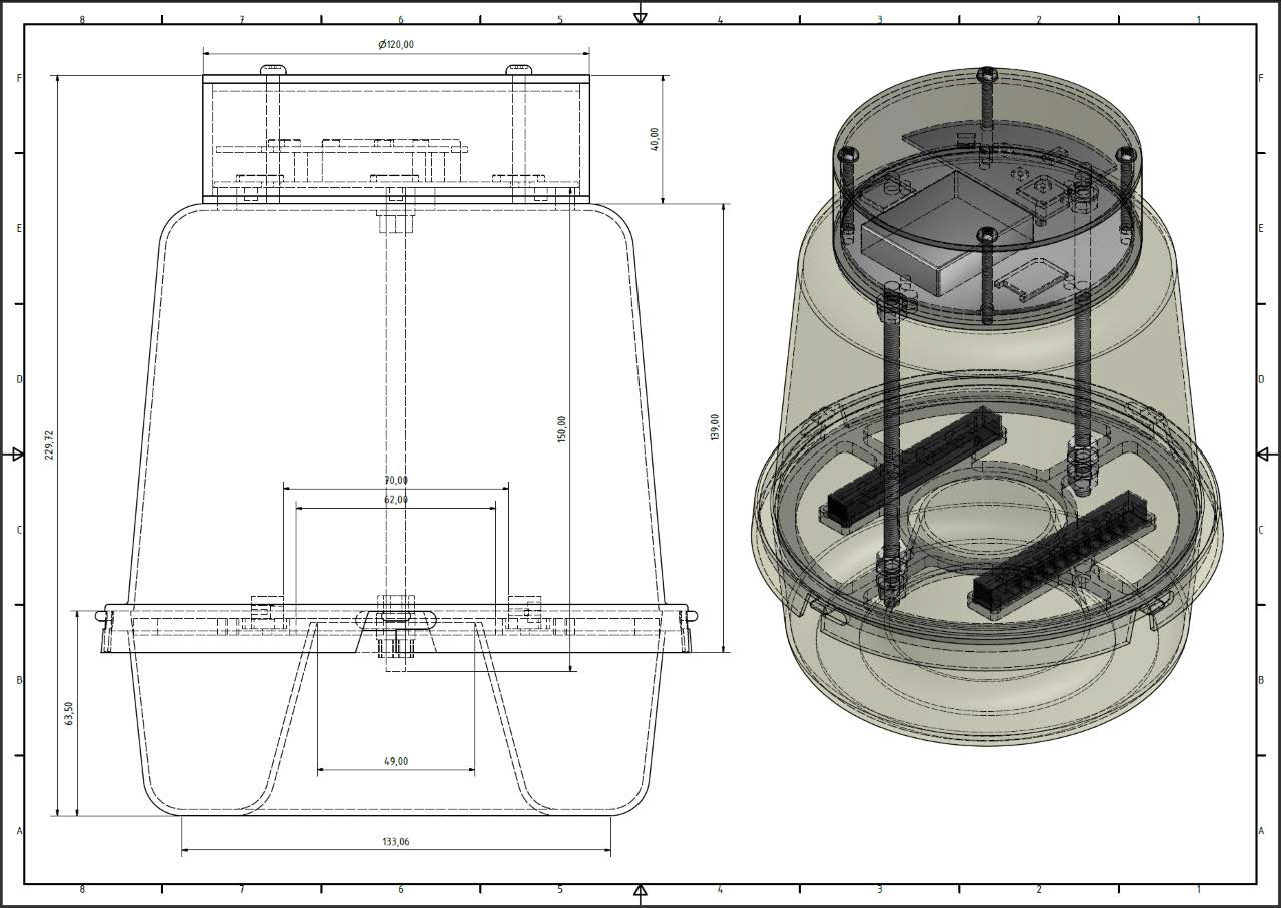

Supplement: S32 Fig — (TIF) [file pone.0140474.s032.tif]

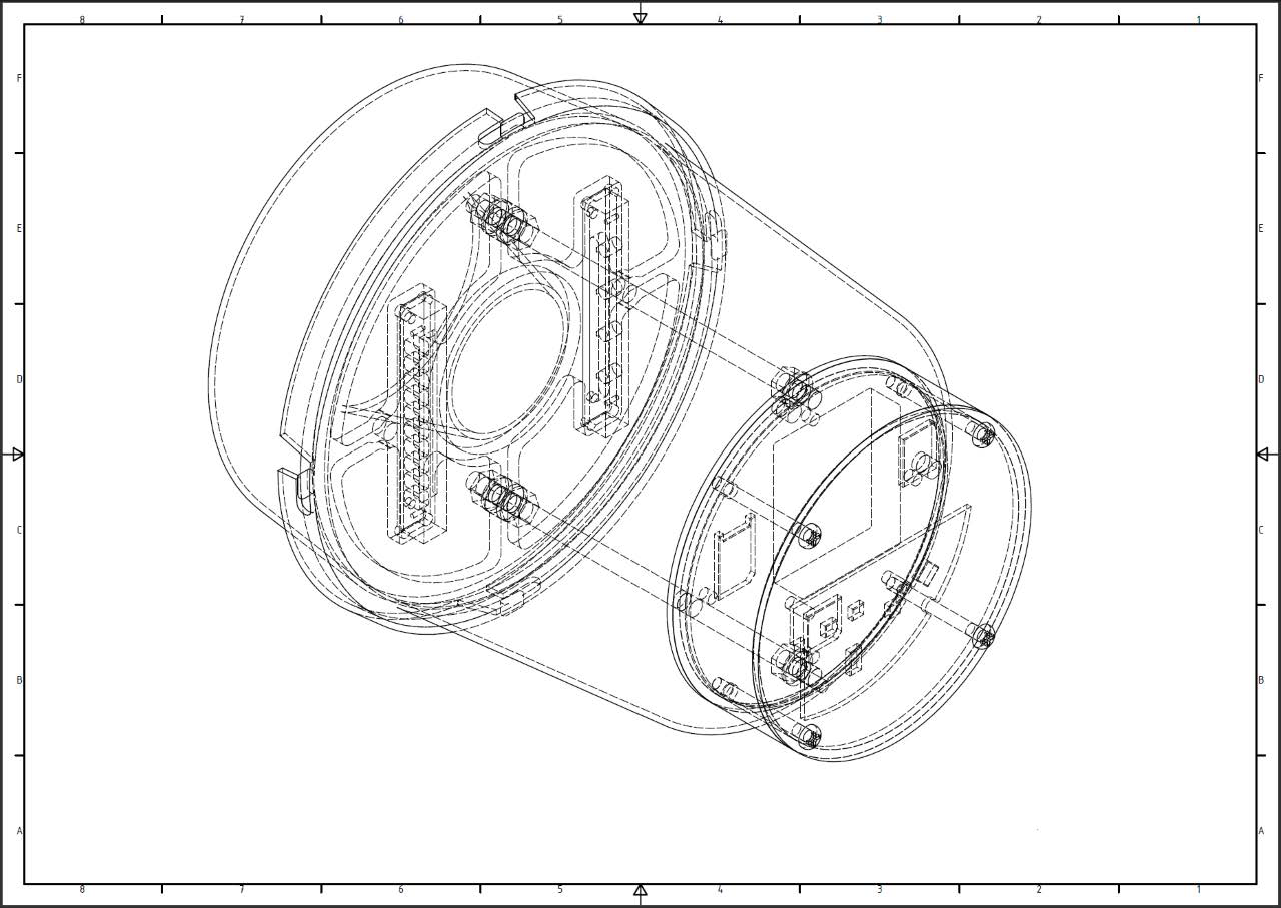

Supplement: S33 Fig — (TIF) [file pone.0140474.s033.tif]

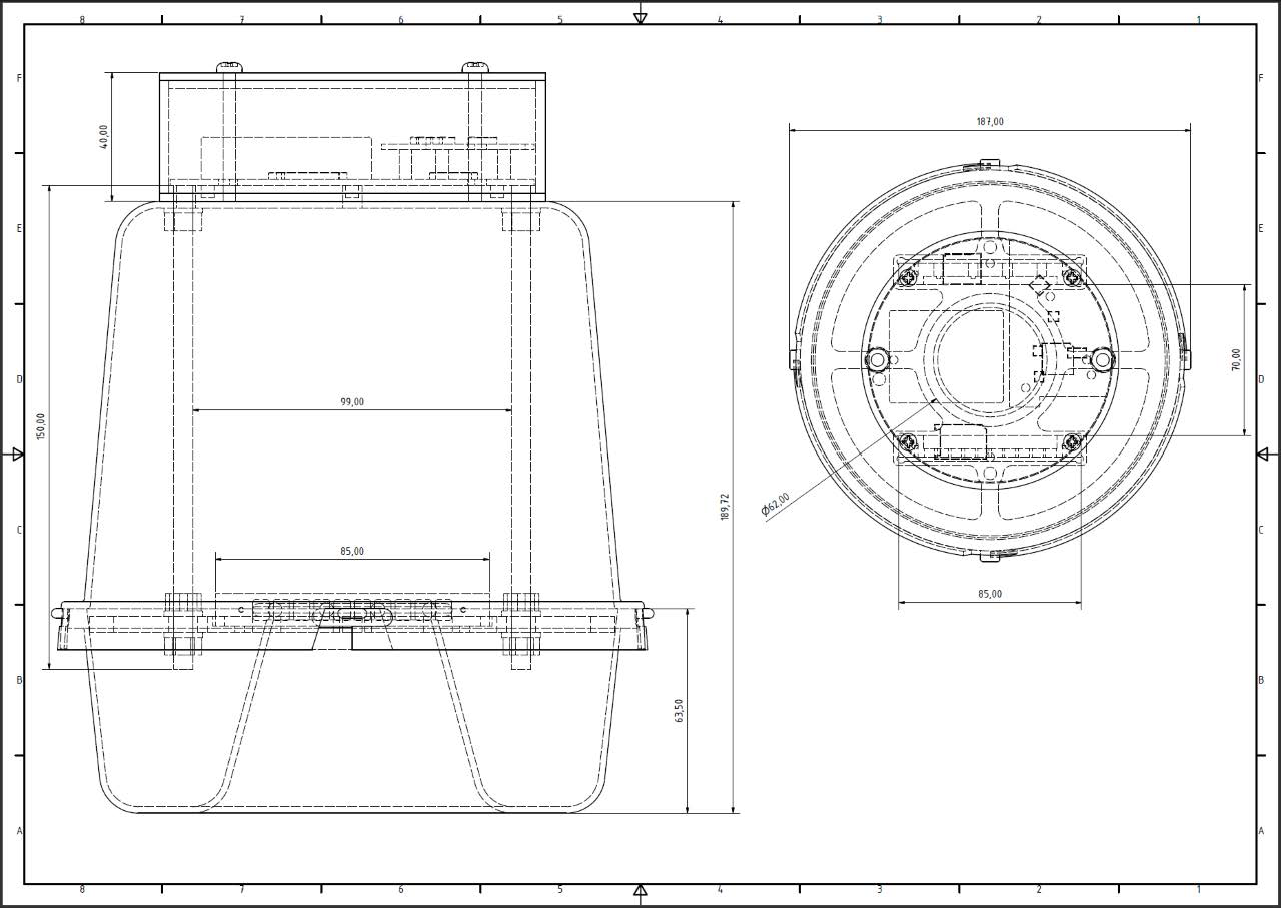

Supplement: S34 Fig — (TIF) [file pone.0140474.s034.tif]

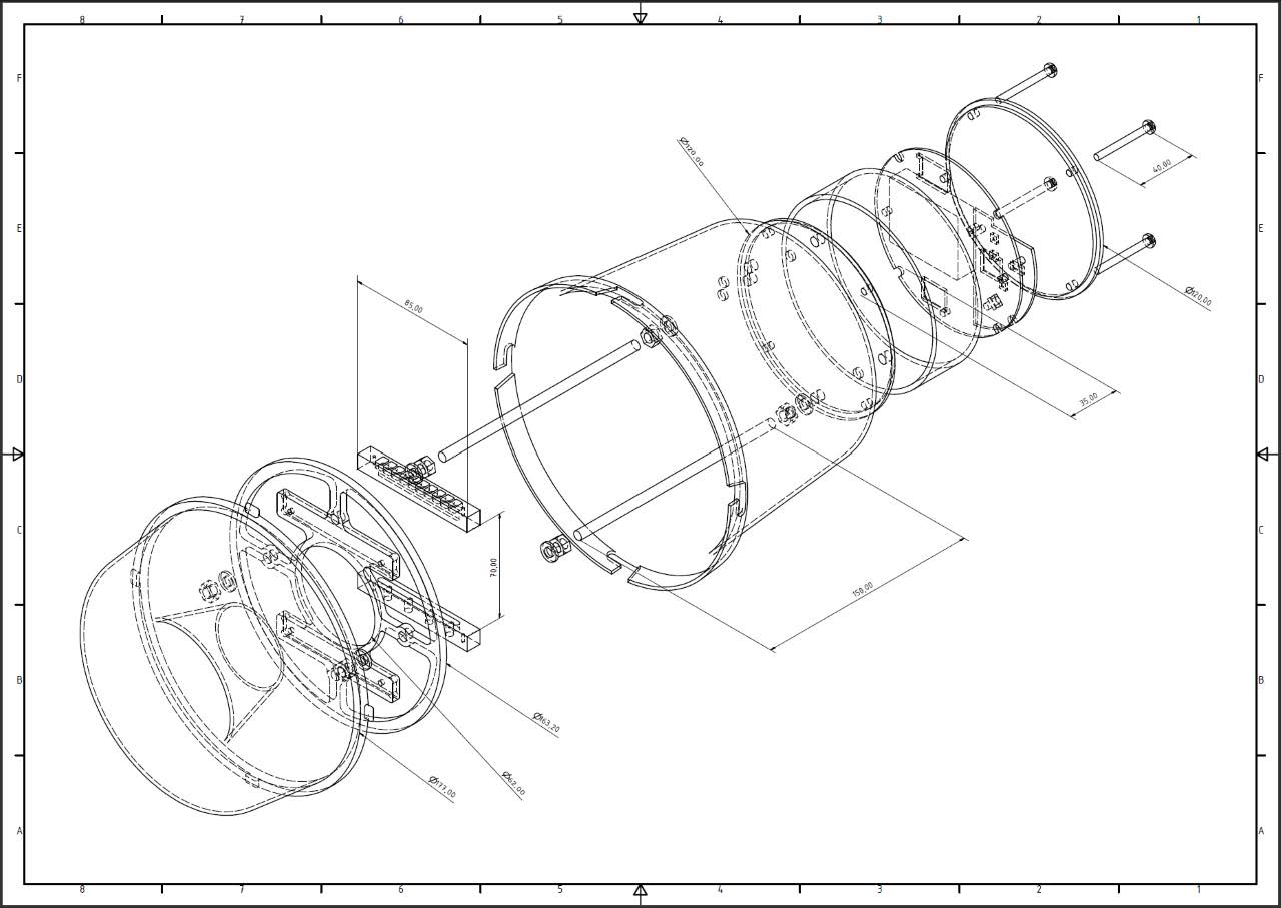

Supplement: S35 Fig — (TIF) [file pone.0140474.s035.tif]
