# Supplementary material for: Correction: Insect Biometrics: Optoacoustic Signal Processing and Its Applications to Remote Monitoring of McPhail Type Traps
Source: PLoS One. 2015 Dec 9;10(12):e0145024. doi: 10.1371/journal.pone.0145024 (PMC4674060; doi:10.1371/journal.pone.0145024)
Supplement: S1 File — (PDF) [file pone.0145024.s001.pdf]

PLOS ONE

RESEARCH ARTICLE

Insect Biometrics: Optoacoustic Signal Processing and Its Applications to Remote Monitoring of McPhail Type Traps

Ilyas Potamitis[1]*, Iraklis Rigakis[2], Konstantinos Fysarakis[3]

1 Department of Music Technology & Acoustics, Technological Educational Institute of Crete, E. Daskalaki Perivolia, 74100, Rethymno Crete, Greece, **2** Department of Electronics, Technological Educational Institute of Crete, Romanou 3 -Chalepa, Chania, 73133, Greece, **3** Department of Electronic and Computer Engineering, Technical University of Crete, Kounoupidiana, Chania, 73100, Greece

* potamitis@staff.teicrete.gr

CrossMark
click for updates

OPEN ACCESS

Citation: Potamitis I, Rigakis I, Fysarakis K (2015) Insect Biometrics: Optoacoustic Signal Processing and Its Applications to Remote Monitoring of McPhail Type Traps. PLoS ONE 10(11): e0140474. doi:10.1371/journal.pone.0140474

Editor: Joseph Clifton Dickens, United States Department of Agriculture, Beltsville Agricultural Research Center, UNITED STATES

Received: July 2, 2015

Accepted: September 25, 2015

Published: November 6, 2015

Copyright: © 2015 Potamitis et al. This is an open access article distributed under the terms of the Creative Commons Attribution License, which permits unrestricted use, distribution, and reproduction in any medium, provided the original author and source are credited.

Data Availability Statement: All relevant data are within the paper and its Supporting Information files.

Funding: We gratefully acknowledge the support of NVIDIA Corporation with the donation of a TITAN-X GPU partly used for this research. The research leading to these results has received funding from the European Union's Seventh Framework Program managed by REA—Research Executive Agency (http://ec.europa.eu/research/rea) (FP7/2007-2013) under grant agreement n°605073 project ENTOMATIC. The funders had no role in study

Abstract

Monitoring traps are important components of integrated pest management applied against important fruit fly pests, including *Bactrocera oleae* (Gmelin) and *Ceratitis capitata* (Wiedemann), Diptera of the Tephritidae family, which effect a crop-loss/per year calculated in billions of euros worldwide. Pests can be controlled with ground pesticide sprays, the efficiency of which depends on knowing the time, location and extent of infestations as early as possible. Trap inspection is currently carried out manually, using the McPhail trap, and the mass spraying is decided based on a decision protocol. We introduce the term 'insect biometrics' in the context of entomology as a measure of a characteristic of the insect (in our case, the spectrum of its wingbeat) that allows us to identify its species and make devices to help face old enemies with modern means. We modify a McPhail type trap into becoming electronic by installing an array of photoreceptors coupled to an infrared emitter, guarding the entrance of the trap. The beating wings of insects flying in the trap intercept the light and the light fluctuation is turned to a recording. Custom-made electronics are developed that are placed as an external add-on kit, without altering the internal space of the trap. Counts from the trap are transmitted using a mobile communication network. This trap introduces a new automated remote-monitoring method different to audio and vision-based systems. We evaluate our trap in large number of insects in the laboratory by enclosing the electronic trap in insectary cages. Our experiments assess the potential of delivering reliable data that can be used to initialize reliably the spraying process at large scales but to also monitor the impact of the spraying process as it eliminates the time-lag between acquiring and delivering insect counts to a central agency.

Introduction

A number of major pests of commercial olives and fruits worldwide are monitored by McPhail type traps as thoroughly described in [1]. Here, we report on the development of an automated

design, data collection and analysis, decision to publish, or preparation of the manuscript.

Competing Interests: The authors have declared that no competing interests exist.

McPhail trap which identifies and counts targeted insects and transmits this information to a central monitoring agency via a wireless communication network. The pest of interest in this study is the olive fruit fly, *Bactrocera oleae*. However, our methods can be used for other insects that are typically monitored with McPhail type traps [2]. Female olive fruit flies lay eggs beneath the fruit surface. These eggs hatch and larvae feed inside the fruit. This causes drop of the fruit or degradation of the quality and value of oil due to increased acidity [2–3]. If left untreated, it is held responsible for losses of 80% of oil value and up to 100% of table cultivars [2–4] and economic damage of approximately $800 million per year [2, 3–7].

For insect species that are of large economic importance such as *B. oleae*, there is a monitoring protocol to be followed on when to initiate a spraying procedure based on insect sampling using traps. The success of the control treatments against *B.oleae* is based on the correct timing; too early will be ineffective due to contact of small proportion of pest population with the insecticide and too late will risk, high pest population levels with inevitably significant qualitative and quantitative losses. For countries, where production of olive oil is a countable percentage of their total income (Greece, Turkey, Spain, Italy, Portugal, Algeria, and Morocco), monitoring and control is handled by the state while agricultural unions and large orchard owners can take further actions. The monitoring protocol is currently based on the standard McPhail trap. Standard McPhail is made of glass, is heavy, fragile and, very expensive compared to plastic ones. They are currently used, mainly because we have accumulated experience of their use over decades. At that time, plastic was not a widespread choice if even existed. Entomologists have been observing the olive orchards for many years using standard McPhail traps. Repeated field observations have resulted into a correlation rule between the optimal time to begin control (mainly spraying with chemical insecticide) and the numbers of captured insect individuals in the McPhail trap. According to 94631/15.4.2002 guideline by the Greek Ministry of Agriculture this rule states that, the first spraying should be initiated when: a) 5–20 *B. oleae* are found per trap for 5 days period, 1 trap per 1000 trees, b) the female to male ratio should be larger or equal to 1:1, c) the percentage of the fertile females is at least 5%, d) the average fruit weight $> 0{,}20$ gr and the core of the fruit starts lignification (the core of coagulation initiation coincides with start creating amino acids in olives), e) favorable weather conditions exist for the growth and development of the insect (temperature 20–35°C and high humidity).

The so called 'decision protocol' is used as a rule of thumb. In practice, reports from monitoring traps are not accepted blindly but serve as supportive evidence. Certified state entomologists adapt the rule to the particularities of different geographical parts of the country and integrate diverge sources of information before granting permission for large-scale spraying. Reports coming out of manual monitoring of traps are accepted with a varying degree of trust. The main difficulty of this procedure is that a large number of glass McPhail traps must be strategically placed on olive orchards, sometimes on distant and remote locations and numerous people should place, maintain and inspect the traps on a 5-day basis from the end of spring till the end of fall. The pest-managers must discern *B. oleae* in the mass of collected maze of dead insects and even extract and deliver the pest to authorities for verification as regards the Greek case. This procedure is complicated, it involves a large number of people that are not always qualified to carry the task but, most importantly, can be easily bypassed by practitioners. Therefore, large economic loss is often reported because of the pest and this is usually attributed by expert entomologists not to the inefficiency of the monitoring protocol but to its opportunistic application that often leads to an 'educated guess' of when and where to start the spraying procedure. The electronic McPhail trap does not revisit other aspects of the protocol but only replaces two stages of it with automated procedures:

A. The classification of the insects is done automatically, as they fly into the trap, the moment they enter, and collective results are transmitted to the central monitoring agency on a scheduled basis without human intervention.

B. Our modified traps are equipped with a Geographical Positioning System (GPS) receiver. Geographical coordinates are transmitted in addition to insect count data. Therefore, one can be sure that the insects counted come from the right place and not from an inappropriate but easier to reach location (e.g. on the roadside) as often in practice. Along with the coordinates, mean values from recordings of temperature and humidity sensors are transmitted in order to be correlated and be used as supportive evidence to assist decision making. Temperature and humidity are registered and transmitted as well, as these environmental parameters are closely correlated to insects' life cycle and reproduction patterns [2].

All processing is performed locally on the trap, while the results are transmitted via a text message, aiming to keep power consumption and operational costs to a minimum. The delivery of data can be remotely set on per event, daily or weekly basis and only the power consumption can set the limit. Therefore the present trap has the potential to monitor the spatial distribution and dynamics of pest populations in real-time.

The reported literature on electronic insect traps that employ optical sensors is sparse [8–10]. In [8], the authors presented a stand-alone device that would count and transmit counts of the oriental fruit fly, *Bactrocera dorsalis*, from the field. This system, however, used the basic functionality of on-off, i.e., counting photo-interruptions due to the passages of insects. It did not involve spectral analysis of recordings and therefore could not discern insect species, an idea introduced by Moore et al. [9]. In [10], a short report has been presented on an instrument that could identify moths in flight but this has not the form or the functionality of a monitoring trap. The first actual prototype trap that integrated all these components and was functional was presented in [11]. This paper is a thoroughly revised work of this idea returning distinct improvements in the following:

a. The trap is modified to account for the fact that, a number of *B.oleae* adults do not fly into the trap, but will rather enter walking and therefore can bypass the sensor.

b. Improved custom-made electronics are developed that are placed on the exterior of the trap as a slim add-on kit and therefore do not alter the internal space of the trap.

c. We carry out detailed controlled experiments with several flying insects including *B. oleae*.

Materials and Methods

In order to produce a usable platform, it is important to balance between the competing needs of accuracy and other priorities such as the cost, real-time performance and power sufficiency. Moreover, considering the platform's long-term exposure to real-field conditions, the electronic equipment should be simple and robust. Sensor's efficiency is also of grave importance, but this should be achieved with low-power consumption and using a low-price sensor. Algorithmic accuracy is highly ranked, but it should be achieved with low-complexity algorithms that will allow real time performance and low power consumption. Construction cost is of importance as well, but this work focuses on monitoring (that requires 1 trap per 1000 trees ~ 10 ha) and not on mass-trapping. The electronic trap, cannot penetrate the routine of agricultural work if it does not fulfill a real need that justifies its added cost, i.e. the reliable timing of the spraying process at large scales and an estimation of where and how dense the problem is prior to and after the treatment. The cost in crop loss due to an erroneous estimate of the

initiation of the spraying process is very large compared to the cost of the electronic traps. Considering that the number of electronic traps to be deployed for monitoring is small, the additional cost of the trap is justified compared to the possible damage.

Provided that a sufficient number of traps is deployed, the monitoring agency can track the status of infestation from day to day for large cultivated areas, spreading up to country level, and assess the impact of the spraying in a timely manner, as there is zero time-lag between the time insects are captured and the time data are reported. This lack of delay between the reality of the infestation and the delivery of the assessment report allows one to efficiently design policies and actions. A time-lag in data reports would mean that people could be reflecting on a situation that may as well have evolved to another unknown state by the time they decide on an action plan. This level of service could only in theory be achieved by manual means, as it would require an amount of funds that would practically be difficult to secure. The device (see Fig 1) presented in this work carries out the following tasks:

a. It attracts insects, either with food-baits or pheromones, as classical McPhail type traps do.

b. As they fly in the trap, an opto-electronic sensor composed of an array of photoreceptors that acts as a receiver and an array of infrared LEDs on the opposite side of the circular entrance guard the entrance by forming a light gate.

c. As the insect flies in, its wings interrupt the flow of infrared light from emitter to receiver. The signal of the wingbeat received is of very high signal to noise ratio (SNR) and resolves the fundamental frequency of the wingbeat as well as several harmonics up to 2 kHz.

d. The analog signal of the wingbeat recording received from the opto-electronic sensors is directed to a microprocessor embedded in the trap that analyses the frequency content (i.e. the spectrum) of the acquired recording. The aim is to extract the fundamental frequency and the way the energy is distributed on the harmonics of the recording. We show that this information, extracted from typical 30–500 ms duration flights of *B. oleae* is enough to reveal species identity of the entering insect. Identity is attributed by calculating a distance metric from the spectrum of the unknown incoming recording to the spectrum of pre-stored prototype spectra of the pest.

e. The insects are counted one at a time while alive and upon their entrance to the trap, and, therefore one avoids to confront the maze of insects that, due to piling and disintegration are difficult to be reliably recognized and counted visually.

f. The electronic circuit stores insect counts and target counts internally and transmit counts according to a preset timetable using the Global System for Mobile Communications (GSM) network. The detection results of all entering insects are accumulated on per-day basis and a Short Message Service (SMS) message, with the results is emitted from the field straight to base. The SIM card and the GSM antenna are embedded in the microprocessor's hardware. The time-schedule of data delivery can be reset remotely by the user, by sending a typical short message (SMS) to the trap.

Signal Processing of Optoacoustic Recordings

It takes a flying *B. oleae* 30–50 ms to cross the beam in a direct quick flight but can reach to 300 ms in slower types of flying patterns and even more in rare cases. The light fluctuation is recorded by the sensor as it crosses the light path from emitter to receiver and subsequently analyzed (see Fig 2 for a typical case of an in-flight recording). Fourier transform can reveal how the energy of the recorded signal is distributed on its constituent frequencies. The basic

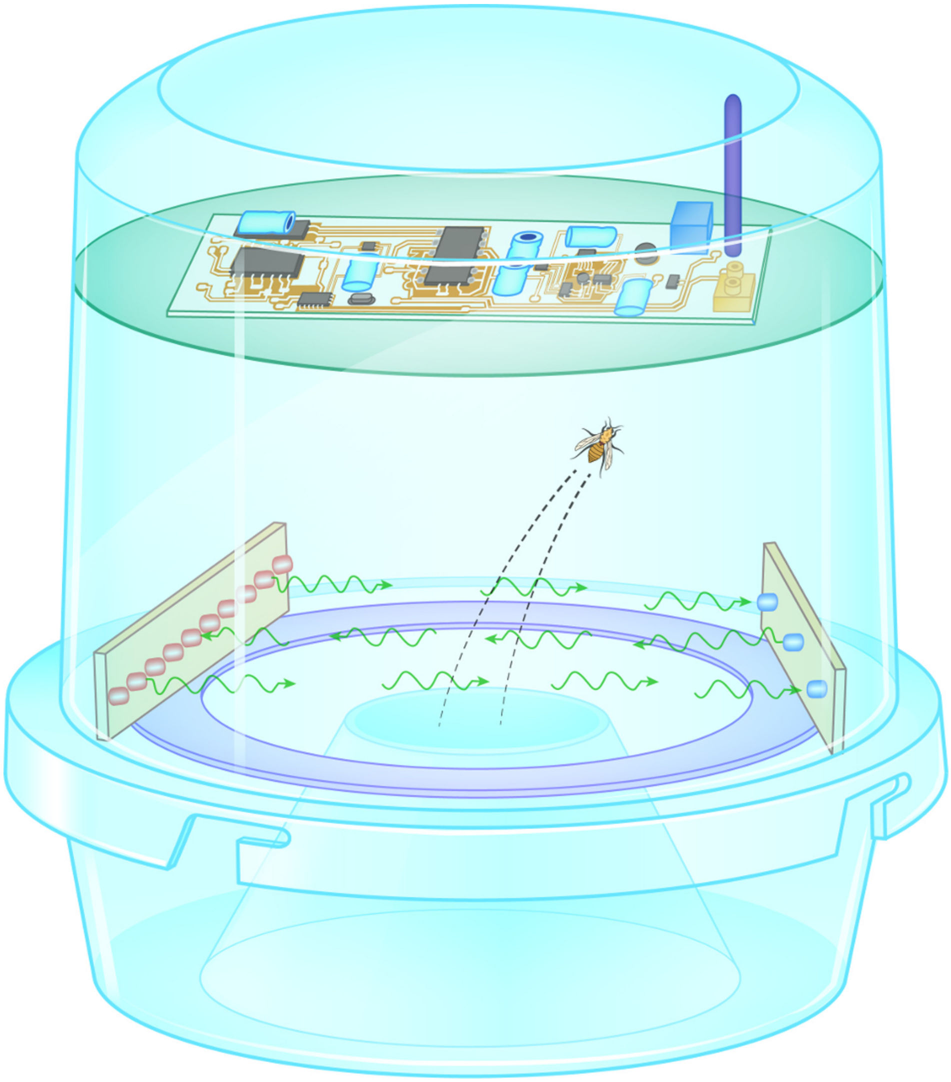

Fig 1. A sketch diagram summarizing the concept of the electronic McPhail trap. The insect flying in occludes with its flapping wings the path of light from emitter to receiver. The electronics of the trap analyze the light fluctuation of the receiver. Light intensity fluctuations constitute a 'biometric signature' directly related to insect's wingbeat frequency, size and shape of its wings. The signature is compared to pre-embedded patterns from the target pest. Finally, counts of the target pest, temperature, humidity and GPS coordinates are transmitted through the mobile GSM network from the field to the monitoring agency.

doi:10.1371/journal.pone.0140474.g001

idea can be grasped in Fig 3, exploiting an analogy to the way in which the human ears and brain discriminate among musical instruments [12]. This figure shows two different real recordings of musical instruments playing the same note (A4). Though they sound alike, the human ear can easily discern from which instrument they originate. They sound alike because they play the same note and share the common fundamental frequency of 440 Hz (corresponding to A4). The sound of the instrument is not a pure sine and therefore, does not only demonstrate a single frequency at 440 Hz but also possesses harmonics (frequencies at integer multiples of the fundamental). The identity of the instrument is captured in the relative distribution of energy on the harmonics. Note in Fig 3- (right) how the flute and the violin share the same fundamental but the distribution of energy on the harmonics is quite different. If the flute and violin in Fig 3 where the sounds made from insects' wingbeat, they would correspond to

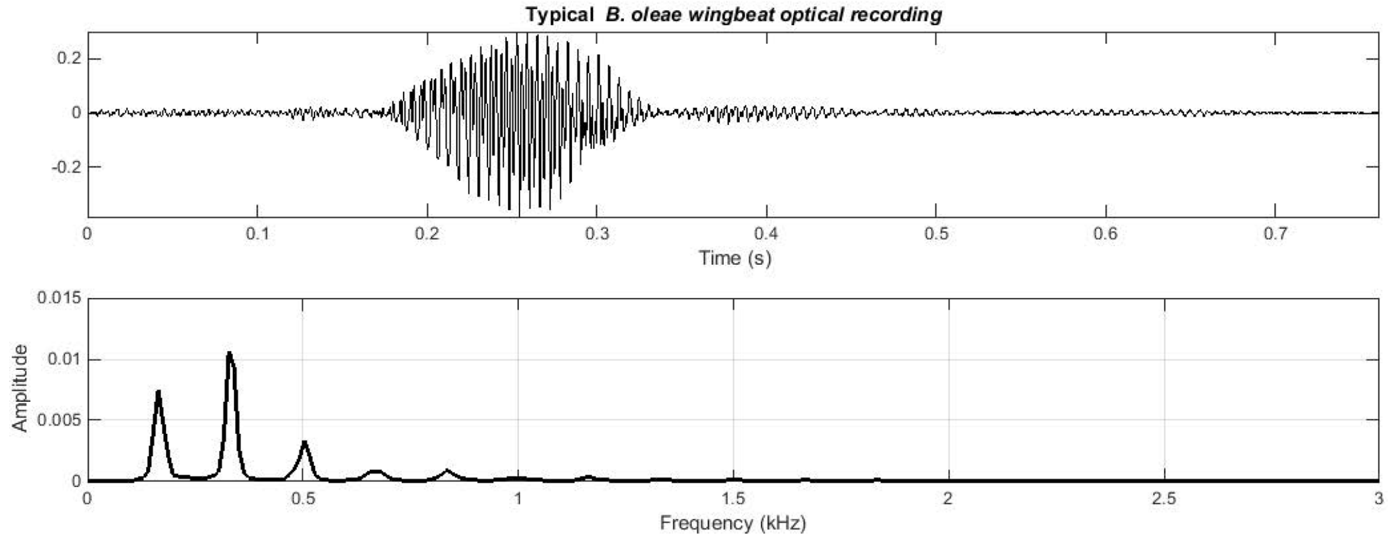

Fig 2. (top) A typical 200 ms *B.oleae*. wingbeat event recorded as the insect crossed the optical sensor (18°C). (bottom) Magnitude spectral density. The fundamental frequency of *B. oleae* typically drifts between 170 and 210 Hz.

doi:10.1371/journal.pone.0140474.g002

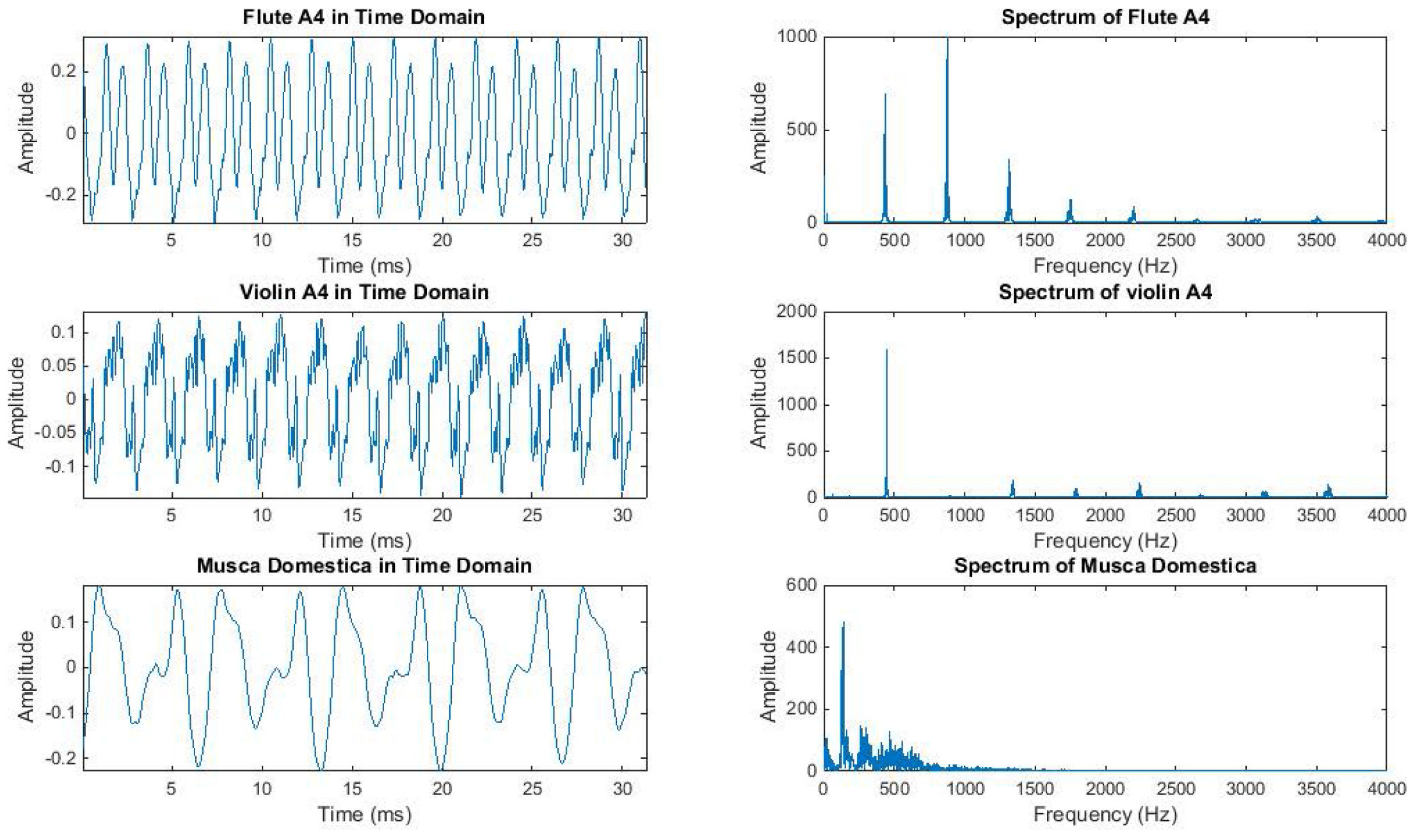

Fig 3. Top: Flute playing Note A4 at 440 Hz. Middle: Violin playing the same note. Bottom: Wingbeat of the insect *Musca domestica*. The time domain plots show 30 ms of data. The spectrum is derived from approximately 2.5 s from the same signals. Note how the flute and violin time-domain plots have the same period but different shapes leading to a different weighting on the importance of the harmonics.

doi:10.1371/journal.pone.0140474.g003

the -not uncommon- case where two insects beat their wings with exactly the same frequency. Insect species can still be recognized due to the differences on the relative energy levels of the wingbeat harmonics. Even the slightest morphological differences (i.e. size, shape, and mass of the wings) as well as stiffness and kinetic properties of the muscle system controlling flight will be reflected on the wingbeat spectrum.

Therefore, during classification we will not only take into account the fundamental frequency, that is the frequency that the insect beats its wings, but also the harmonics produced by the movement of the wings. The harmonics produced are related to the size and shape of the wings. The slightest difference leaves an acoustic imprint. Insects of the same species (e.g. *B. oleae*) will have differences in the spectrum of their in-flight wingbeat, as they are unrepeatable biological organisms. These differences have an imprint on the fundamental frequency which is characteristic for each species and drifts slightly around a central value but also on the distribution of harmonics. The spectrum of individuals of the same species do not have the almost absolute repetitiveness of a note of a musical instrument. However, their spectrum follows a consistent, recognizable pattern and the differences in the spectrum of the same species (inter- species spectral variability) are expected to be smaller compared to other insects of different species whose wings have different shape, size and wingbeat frequency (intra- species spectral variability). As reported in [13] for the case of bumblebees, *B. oleae* is also expected to hold a rather constant wingbeat frequency, in a given temperature, regardless of the speed and flight pattern they hold.

The 'Candlestick' Sensors

The electronic McPhail trap, while operating in the field, compares the recording of the flying in insect with pre-stored recording of the pest that act as prototype patterns. Based on this comparison, it decides on the identity of the incoming unknown insect according to a distance measure. These prototype patterns are recorded in the laboratory, by placing sensors (see S1–S5 Figs) that sense the wingbeat of flying insects inside spacy insectary cages (see Fig 4 and S6–S19 Figs). We need to record insects demonstrating flight habits and behavioral modes close to the ones encountered in nature. We hereinafter describe how to record these prototypes, by starting from where and how to produce a large number of insect specimens: The female *B. oleae* egg-lays inside ripening fruit of olive trees in the field, by making a puncture with the ovipositor into the skin of the olive fruit. This act releases a tiny amount of oil out of the fruit and the trained human eye can see from distance that the olive tree is greasy. We collect greasy olive fruits that are visually confirmed to carry an oviposition puncture, from trees and place them on a sieve.

The larvae inside the olive fruit are in various instars and they feed upon the pulp until they exit, usually as third instar larvae which pass through the sieve to pupate. Then they are collected and grown in an insectary cage. As larvae turn into adult insects, we supply them with yeast hydrolysate-sugar diet and water to sustain them to life. We keep only first generation insects, as breeding generations of insects in captivity results into degeneration that might affect the flying mechanism. Denoting as day 0 the time we collect olives from trees, then at day 0–12 the 3^{d} instar larvae come out and, from day 8–20 they turn into adults. The same day (few hours later), they can fly. Adult insects are free to fly without induced stress inside the cage and while they fly they pass incidentally through the 'candlestick-like' sensors placed inside the cage (see Fig 5). We place a large number of *B. oleae* only in cages and the recording of their wingbeat occurs the moment they pass through the rectangle of the sensors on a random basis. The size and shape of the sensor is designed is a way that is possible to pass it through the entrance of a normal insectary cage. This innovative setting allows us to take

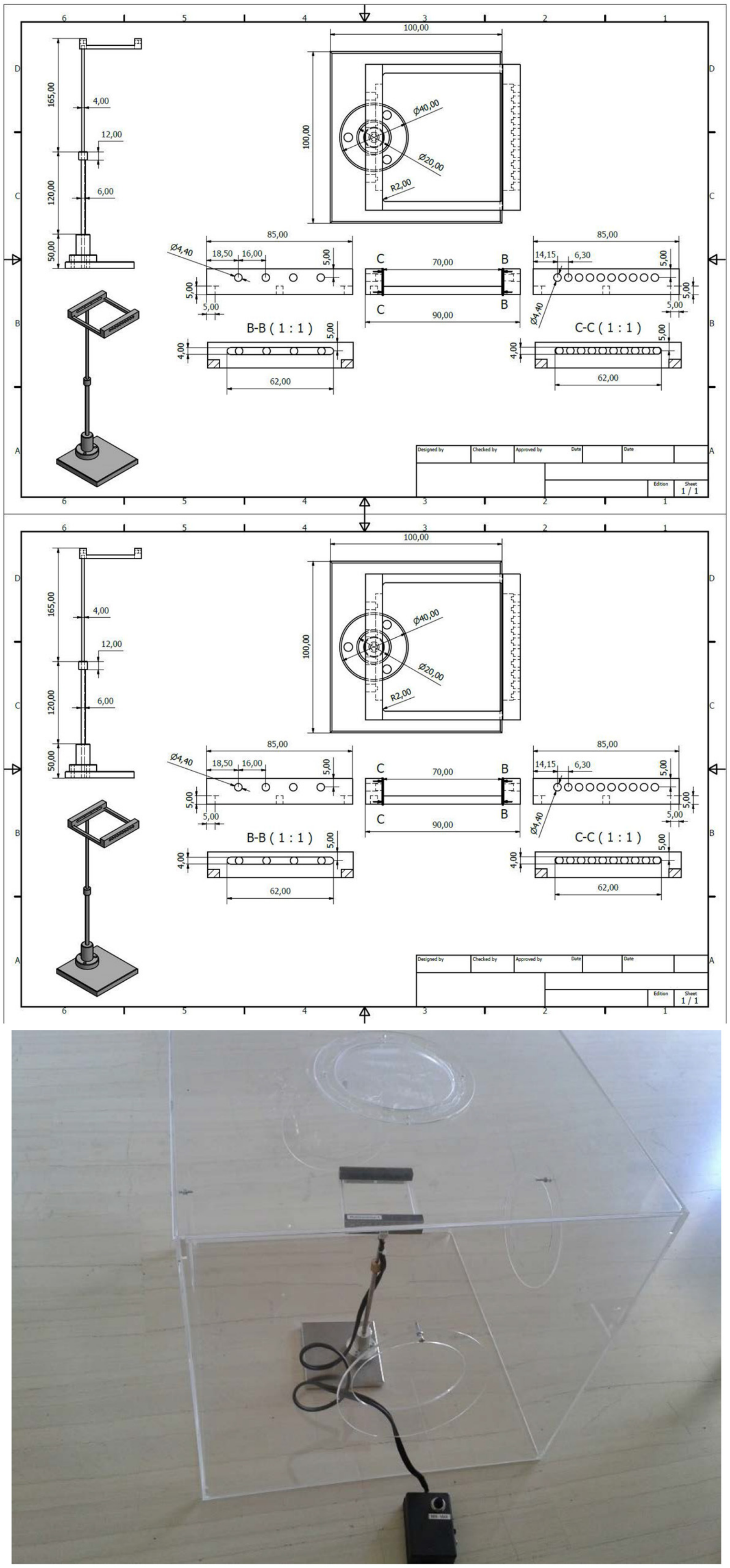
B-B (1 : 1)
C-C (1 : 1)
Designed by
Checked by
Approved by
Date
Edition
Sheet
1 / 1

Fig 4. (top) A diagram of the candlestick optoelectronic sensor in mm, (bottom) the optical sensor in its final standing inside the insectary cage. As insects fly freely in the cage, some of them randomly pass through the square thus interrupting the light path from emitter to receiver.

doi:10.1371/journal.pone.0140474.g004

effortless recordings of a large number of flight cases in a practical way, as insects are generally difficult to manipulate. The sensors can record unattended for a number of days, or indefinitely by connecting the kit containing the battery to a charger. In Fig 5, the insectary cage contains about 500 adults of *B. oleae*. Depending on the insect density of the cage, one can have an order of hundreds to thousands wingbeat recordings in just few hours.

Comparing Photodiode and Phototransistor Arrays

We have fabricated two versions of the optoelectronic device; the receiver is either an array of photodiodes or phototransistors (see S20 Fig), while the emitter is always an infrared light source. Experimentation with a laser as a light source has been reported in [14]. The laser source consumes more power that the infrared light and produces comparable results to the infrared light source. Since power sufficiency is crucial for a device working in the field, we choose the infrared light source as an emitter. Both optical sensors are embedded in the same cage thus recording the same insects at 20°C, 60% humidity. We observe that photodiodes can track better the harmonics of the flying insects compared to phototransistors. Phototransistors have a slower rise and fall time, at around 16 μSeconds while the photodiodes around 80 nSeconds. Moreover, the reception area of photodiodes is slightly larger than phototransistors, allowing for longer recordings, as the insect spends slightly more time in the field of view. The same figure demonstrates that the insects indeed hold a relatively constant wingbeat regardless of the flight pattern and angle of pass through the detector. Since the power spectral density is derived by hundreds of free-flying insects performing thousands of passes, large variations in the frequency of the wingbeat would appear as flat areas in the spectrum which is not the case here as can be verified in Figs 6 and 7.

Comparing Optical Sensors to Microphone Transducers

The waveforms generated by the flight sounds of *B. oleae*, were also recorded by a low noise, small aperture gun microphone (MiniDSP, UMIK-1 omni-directional measurement

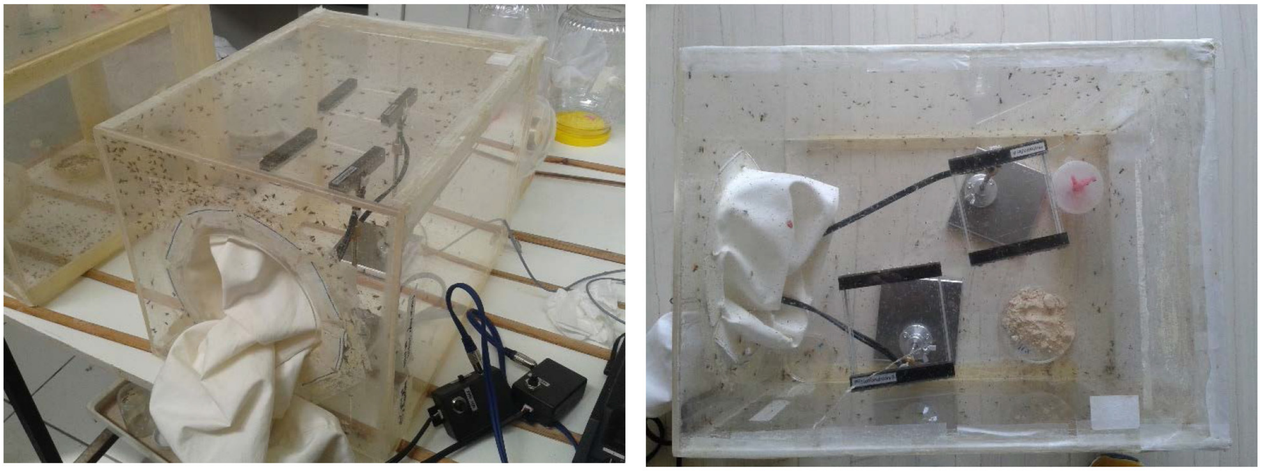

Fig 5. The optical sensors in their final setup, inside insectary cages with *B.oleae*. Each time an insect incidentally flies through the rectangle, a recording of a wing flap is acquired.

doi:10.1371/journal.pone.0140474.g005

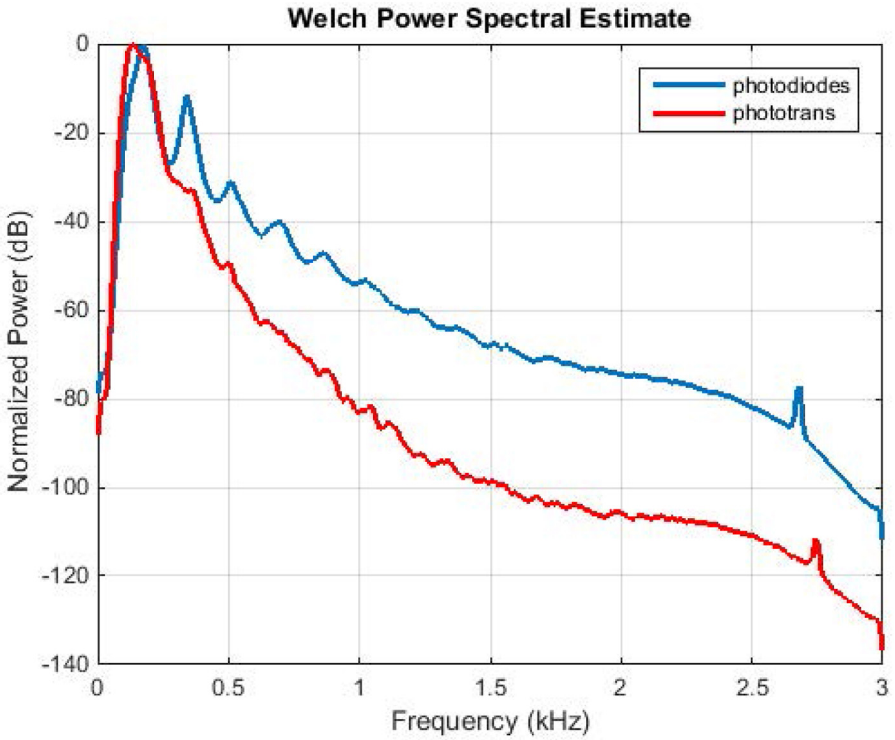

Fig 6. Normalized Power Spectral Density of *B. oleae* wingflap as measured by a photodiodes array vs a phototransistors array. Both sensors resolve the fundamental frequency of the wingbeat around 180 Hz. The diodes resolve better the harmonics at multiples of ~180 Hz.

doi:10.1371/journal.pone.0140474.g006

microphone) inserted in the cage containing the same insects from which the optical recordings were taken. We recorded 24 hours of insect activity. In Fig 7 we compare the power spectral density of the microphone recordings to the spectrum of the photodiodes sensor recordings. The optical sensor follows closely the microphone for the first 4–5 harmonics. The microphone can resolve higher harmonics as well. The recording took place at the same environmental conditions as the experiments with the optical sensors.

One should not rush to see a benefit of the microphone to this task compared to the optical device:

The optical sensor records an event only for the time that the light from emitter to receiver is interrupted and therefore the wingbeat events are ad-hoc shorter in time than events recorded by a gun microphone in a small cage containing a large number of insects of the same species. Special measures have been taken in order to make possible a microphone recording in the lab and these measures cannot be applied when operating in the unconstrained field: The cage was placed in a quiet chamber in the laboratory providing low-noise conditions to study insect sounds. In normal operational conditions, the microphone would pick up sound from all directions, as the field is exposed to relative high noise levels (due to wind, cicadas, birds and machinery), thus making unpractical to be used for the task at hand.

We observed closely the behavioral mode of *B.oleae* by inspecting visually the entrance of insects to the sensors in Fig 5 and by tagging the corresponding waveforms. We group the behavioral mode in two large sub-groups:

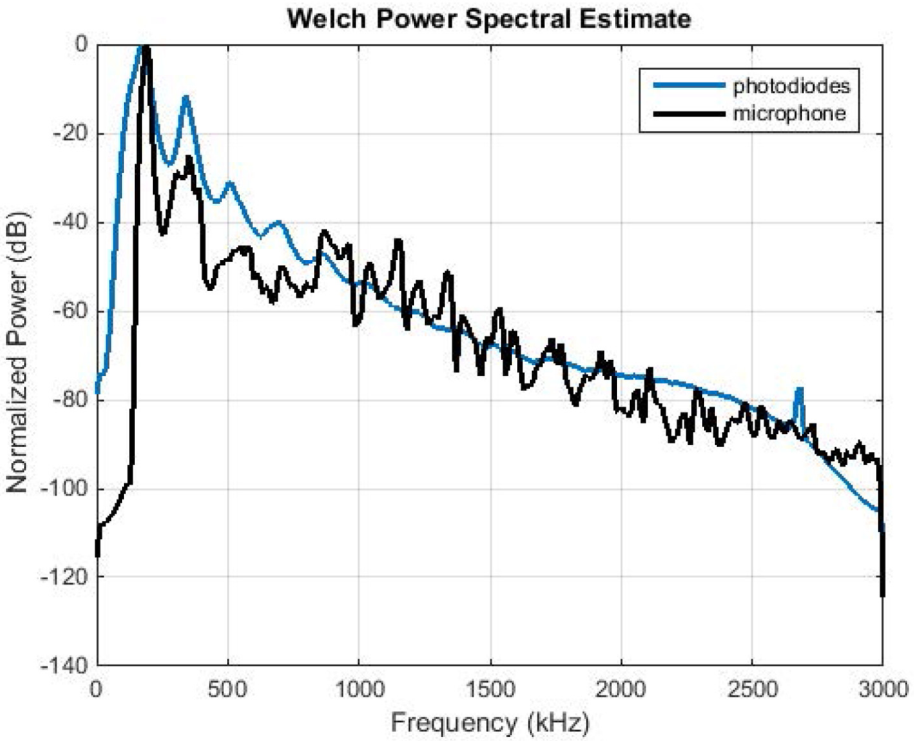

Fig 7. An optical sensor and a microphone transducer embedded in the same cage holding *B. oleae* insects. PSD of photodiodes array vs microphone transducer. Both sensors resolve the fundamental frequency of the wingbeat and have good accordance until the 5th harmonic. In this particular controlled setup, the microphone can resolve higher harmonics as well.

doi:10.1371/journal.pone.0140474.g007

a. Direct, fast crossing of the sensor either by moving upwards or diving in at an almost straight path. It takes the insect 30–50 ms to cross the field of view of the sensor. In Fig 8 we present a dozen of different events of this behavioral mode and their associated frequency analysis.

b. Slower flying movements like if the insect is strolling. The movement may involve coordinated turns and hovering. This mode of flight can take roughly 50–300 ms to cross the receiver but depending on the angle of the entrance can reach even more.

Sensor Electronics

The electronics can pick up interference from electric lamps and must, therefore, be operated only in daylight or DC-powered light in the laboratory. They also operate in total darkness. It is possible to use electronic configurations that are immune to interferences but this increases the cost of the sensors and we need to keep the cost down in order to be acceptable by the end-users. More sophisticated electronics that send 60 kHz pulses instead of continuous light to the flapping wings of the insect that modulates the amplitude of the high frequency carrier, clean the low frequencies from interferences and demodulate back the wingbeat at acoustical frequencies are immune to low frequency interference variations caused by AC powered electric light [14] (see S1 Video). Another way to deal with interferences is to pick them up and subtract them as suggested in [15]. The latter solution has the drawback of needing calibration. As interference from electric light is not expected when the trap will be operational in olive

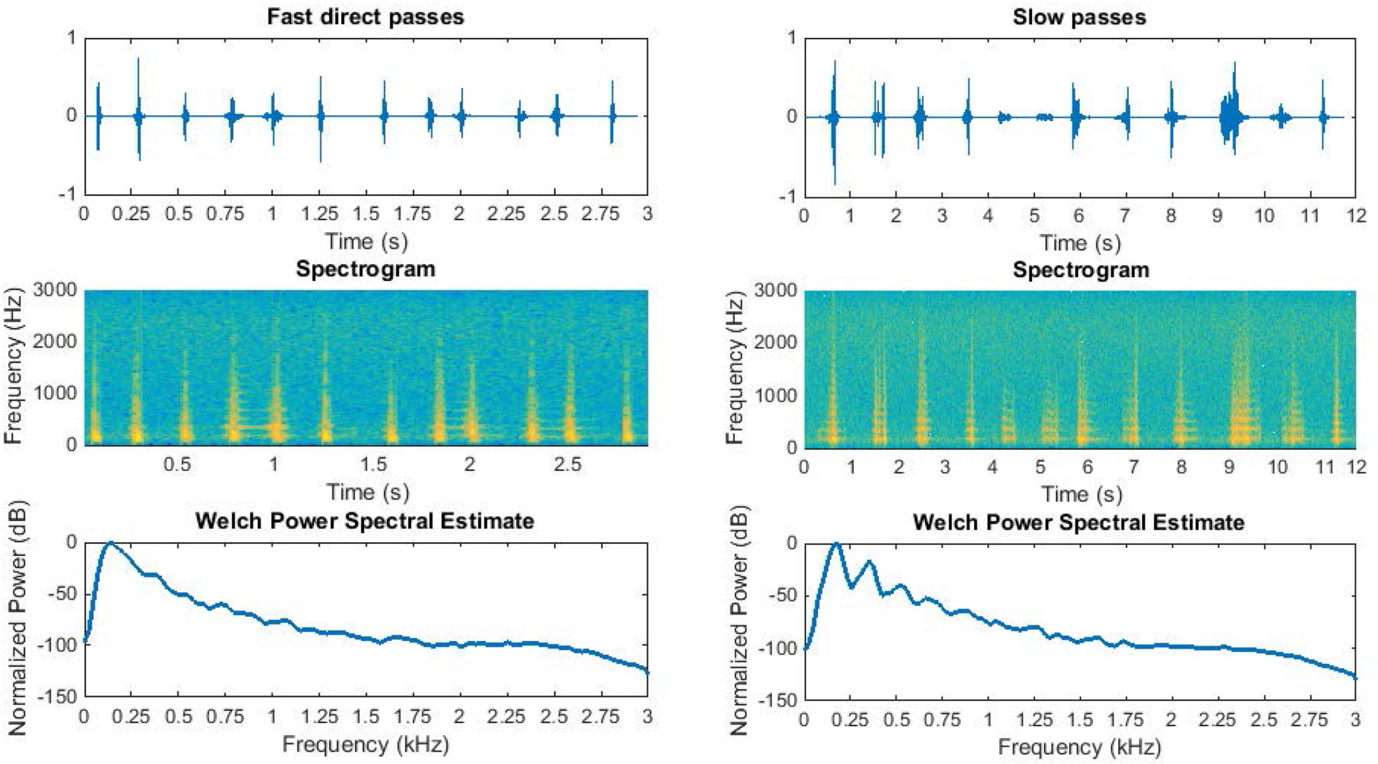

Fig 8. PSD of photodiodes array resolving the spectrum of two typical flying patterns: straight, short-time passes (left) and slower maneuvering passes of insects (right).

doi:10.1371/journal.pone.0140474.g008

orchards, this available technology is not employed in this trap, so that we keep the cost down (see Appendix for a cost analysis).

Light for the sensor was produced by 4 infrared LEDs, operating at 940nm, connected in row (see S12 Fig). The multiple LEDs solution was chosen for two reasons: a) to ensure uniform light distribution across the entrance of the trap, b) to reduce power consumption. If one employs a single LED one must increase the distance between emitter-receptor to cover the whole entrance. This in turn leads to an increase of the current that needs to be supplied (a single LED needs around 26mA). The use of 4 LEDs reduces this distance from emitter to receiver, allowing a current of 2,7mA to be used, which in turn requires roughly 1/10 of the power of a single LED.

Wingbeats are low in energy level. Despite this low power level the electronics provide recordings with very high SNR (see S2 Video). The recordings of Fig 2 and Fig 8 have not been post-processed with noise enhancement algorithms and one can note that, in the absence of an event, noise is of very low amplitude. The high-pass filter embedded in the circuit cuts the very low frequencies that are due to the main-body movements of the insect. In the Supportive Information section we include detailed schematic diagrams of main-board electronics in S21–S29 Figs.

Embedded Software

The embedded platform runs a constantly-looping program (see Fig 9) which processes data captured by the sensors. The board can be programmed in C/C++ using any Atmel AVR compatible programming environment, including the Arduino IDE if ease of use is a prime goal. The line-level output from the optoelectronic sensor is routed through a series of operational amplifiers in order to ensure the signal's amplitude is adequate. The signal is then processed by the Analog-to-Digital (A/D) converter of the embedded platform, capturing 512 samples coded with 10-bit resolution, at 4 kHz sampling rate, with the resulting data being stored in a circular buffer. The signal's root-means-square (RMS) value is calculated and, if it exceeds a pre-defined threshold, we call that an event has occurred, i.e. an insect has crossed the sensor's beam. This increases the counter of recorded events by one and triggers the second stage of processing of the captured data. In more detail, a (Hamming) window function is applied on the data and then a Fast Fourier Transform (FFT) is used to get the frequency domain representation of the signal. The magnitude values of the FFT are then normalized in order to account for possible gain differences, using the following function that was found to work better that normalizing with the max value [16–17]:

$$s_i/\sqrt{\sum_{i=1}^{N} s_i^2}\,, i = 1, .., 512. \tag{1}$$

Once the data is normalized according to (1), it is compared to the reference spectra stored in microcontroller's EEPROM. In its current iteration, six reference recordings is the maximum that the microcontroller's EEPROM size of 4 kB allows, but this can trivially be expanded in future versions of the hardware platform.

Based on the comparison of the captured signal's spectrum to the stored references, and depending on if it matches the pre-defined similarity criteria, the investigated event can be classified as a match or not. In the former case, the counter of target species matches is increased by one and auxiliary data is collected by the system's sensors (namely temperature, humidity and GPS coordinates). The resulting dataset transmitted via SMS to one or more recipients.

The Assembled Electronic McPhail Trap

The trap carries a photodiodes array sensor identical to the one used to record the reference patterns in the laboratory (as depicted in Fig 5). The identicalness of the sensors is essential in order to ensure that the sensors will not induce mismatch between wingbeats recorded in the lab (see Fig 10) that serve as prototypes and recordings of the unknown, entering insect. The emitters are placed in a dark plastic thin container in order to be held aligned opposite to the receivers. The same type of containers provide shade to the optical receivers. Environmental light and reflections must be attenuated as much as possible allowing mostly emitters' light to reach the receptors. It is important to avoid physical light coming from outside the trap to act as an emitter as then insects inside the trap may modulate physical light and give false measurements. The electronics are placed as an independent add-on, attached to the plastic top of the trap, so that we effect minimal disturbance of the internal space of the trap (see Fig 11, S30–S35 Figs and S3 Video). It is our effort that the insects sense a typical McPhail type trap, as the literature has tested extensively insects' response to it. There is an intentional gap between the border of the inner entrance of the inverted funnel and the support of the sensors (see Fig 12). Video recordings of traps in the field as well in the laboratory have shown that *B. oleae* enters the trap either by flying or by landing on the outside of the trap and walks up through

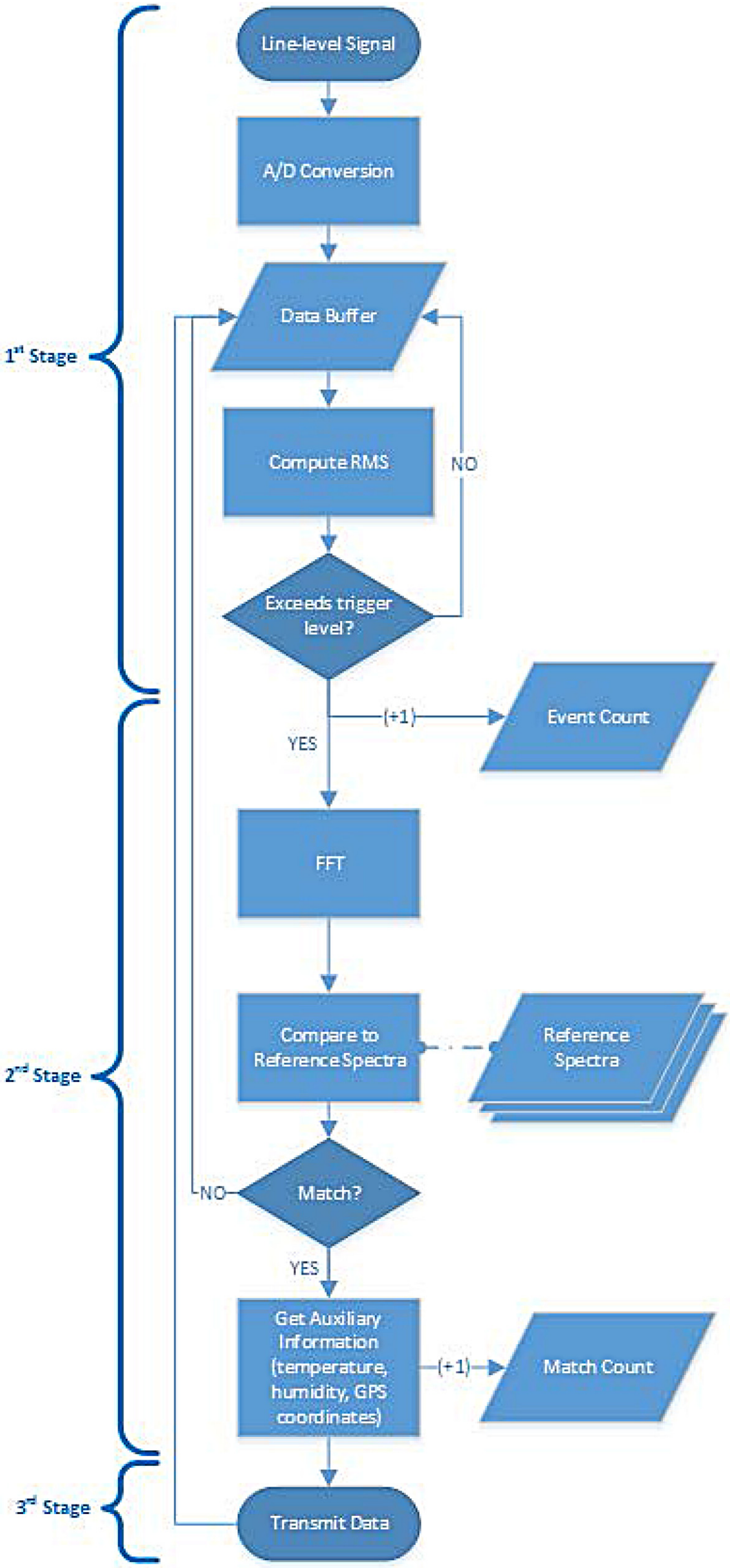

Fig 9. Flow chart of processing stages. Processing is continued to the next stage only in case of a positive event otherwise transmission stage rests in sleep mode

doi:10.1371/journal.pone.0140474.g009

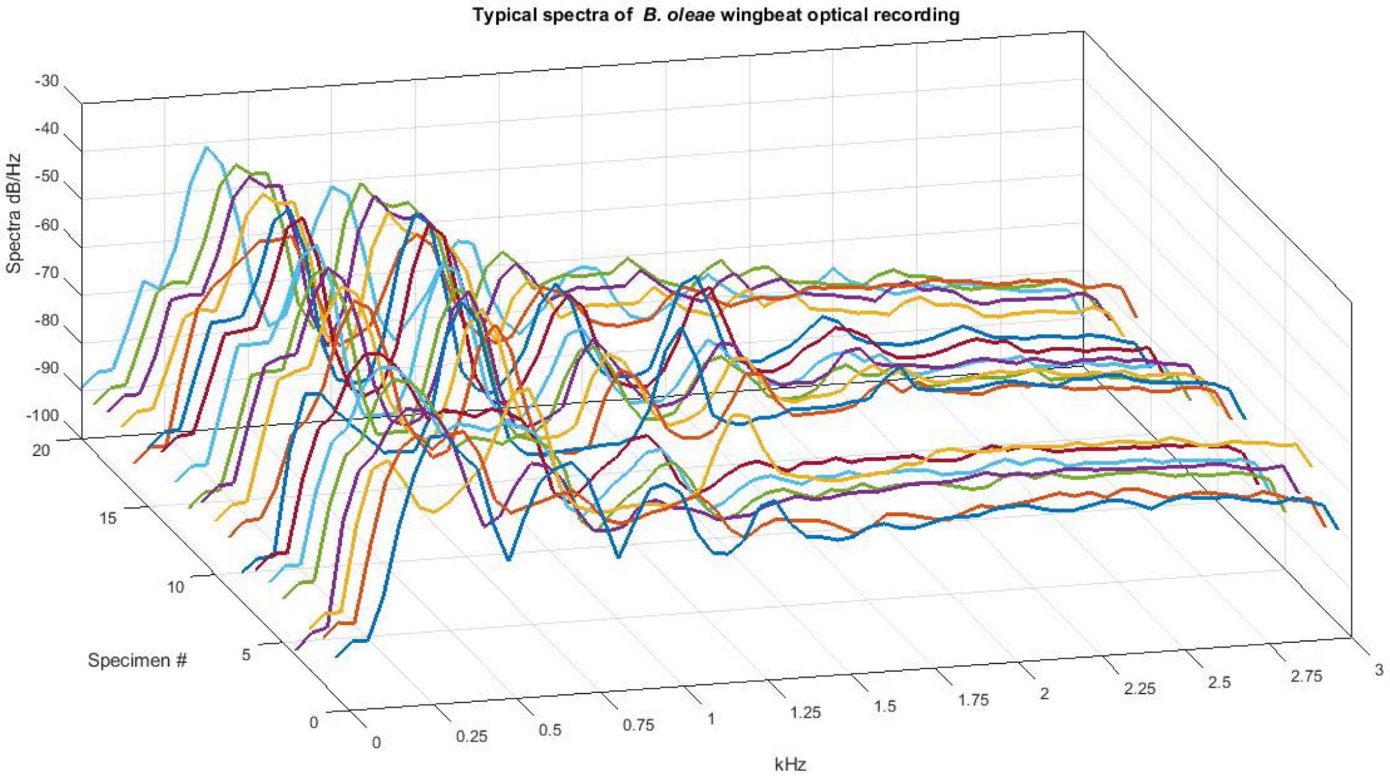

Fig 10. Spectra of 20 different cases of *B. oleae* optoacoustic, in-flight recordings.

doi:10.1371/journal.pone.0140474.g010

the outer entrance of the inverted funnel to the inner entrance following the chemical signals emerging from the entrance. Then, in the vast majority of cases, walks along the circle on the thin, uncomfortable border of the entrance and it typically flies from the internal border to the top following the higher concentration of the evaporated bait gathered in the top part of the trap. The gap is necessary, and in-fact, an efficient and cost effective solution that ensures that the insect will not have an available path to by-pass the sensors by walking. The sensor is able to detect and record an insect as long as it flies (see S1 Multimedia for optoacoustic recordings of several flying insects and S4 Video).

Uploading the detection results using GPRS

We have experimented with the applicability of GPRS that is available on the communication chip embedded in the trap and connects the trap to the Internet. It can be used to transmit results as SMS messages, including counts of detected insects and associated metadata (i.e. battery status, GPS coordinates, temperature and humidity) to a web interface. In line with the goal of producing an accessible and cost-effective solution, it was important to implement this web/backend functionality without resorting to any costly and/or hard to setup and operate software platforms. To this end, we exploited the dweet.io platform (https://dweet.io/), a free web service that facilitates simple posting of data online from various internet-enabled devices. To post the data online, each trap uses GPRS communication (HTTP in specific) to access a specific URL, formatted in a way that passes the associated parameters to the dweet.io servers.

As we want to aggregate and visualize this data, we need to extract it from servers and forward it to another platform. For this purpose, we make use of another free service, namely

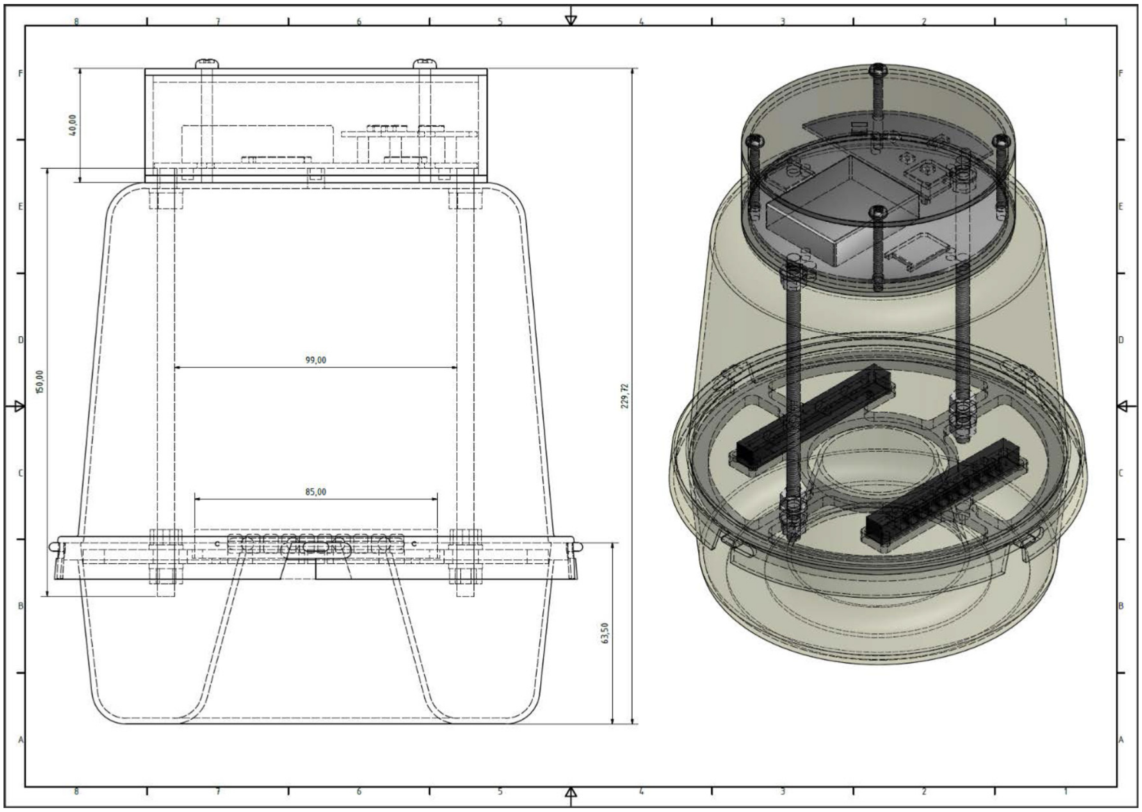

Fig 11. A detailed 3D CAD of the electronic McPhail trap. Dimensions are in mm.

doi:10.1371/journal.pone.0140474.g011

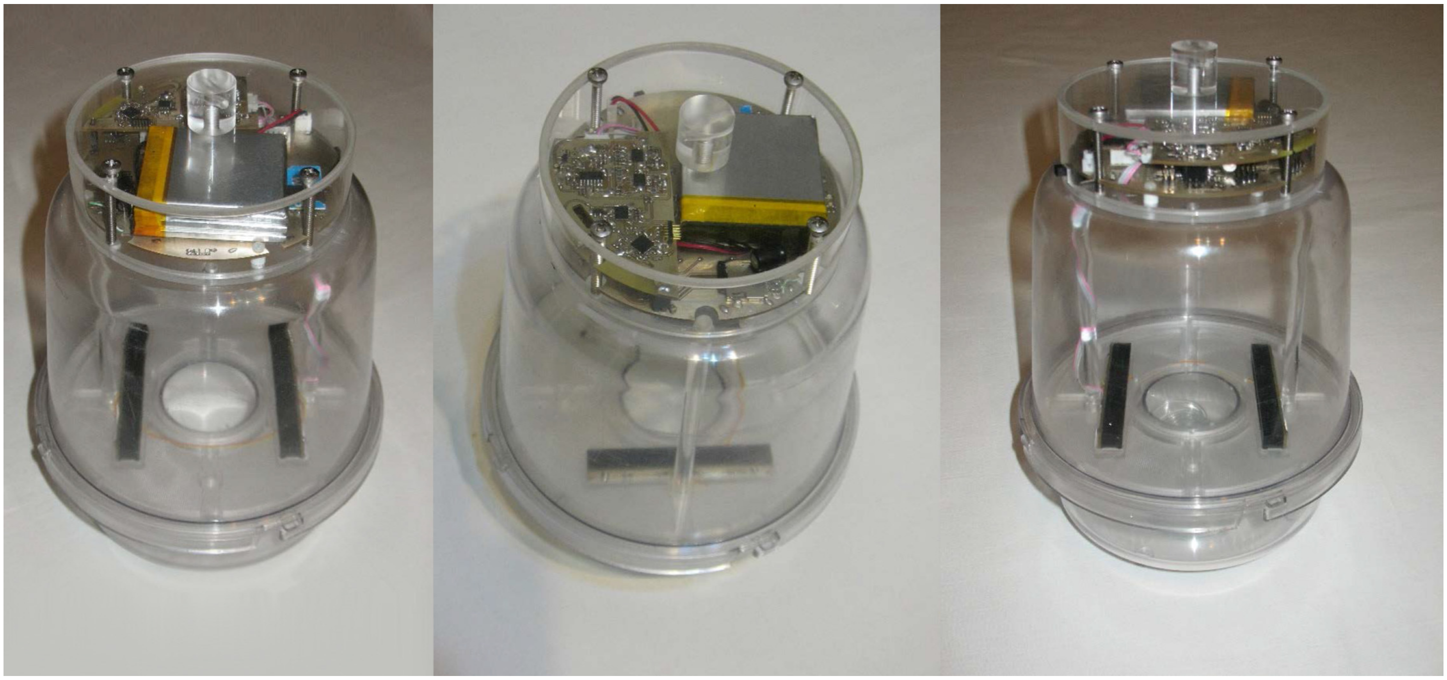

Fig 12. Different views of the prototype electronic trap.

doi:10.1371/journal.pone.0140474.g012

Freeboard (https://freeboard.io), which allows the visualization of the transmitted information in an intuitive, and easy-to-setup manner. We then, create our dashboards which may feature various visualization options, such as counters, graphs, GIS maps and other user interface elements. Thus, we combine the two platforms to enable real-time monitoring of the status of our deployed traps though a user-friendly online web interface, at no extra cost.

A simpler transmission method was also implemented, which involved having the platform send a daily SMS message with target counts and total events to a predefined mobile phone number. This way, the owner of the olive orchards being monitored can get updates, even when no internet access is available and/or when one is not computer inclined and thus not comfortable with using the web interface.

Although we plan to study the case that the traps form a wireless network that collects data from each trap using hop communication and there is only one transmission from the gate to the central station we did not choose this option in this work. The traps must be strategically distributed to cover the whole area of interest and this may include distance of several kilometers, hills and other obstacle that can prevent nodes to contact each other. Moreover, farmers would rather avoid dealing with the complexity of technology and possible problems with incompatibility of diverse technologies.

Results

The objective of the verification module is to examine if the generated optical fluctuations modulated by the wingbeats of the incoming insect belong to *B. oleae* or not (a two class problem). Therefore, the analog recording of the optical sensor is forwarded to the microprocessor placed on top of the trap where is subjected to Discrete Fourier Transform in order to get the frequency signature of the incoming wave and subsequently compared to pre-embedded frequency signatures of the target insect derived from free flights in the laboratory.

In Fig 10 we show an example of 20 distinct free-flight passes of *B.oleae* adults taken with the candlestick sensors, to demonstrate the consistency of the frequency signature.

One can note by looking at Fig 10, but also from inspecting the spectrum of hundreds in-flight cases that the spectrum provided by the optoacoustic sensors is invariant to the entrance angle and behavioral mode of the flying insect. This is reflected on the fundamental frequency as seen in Fig 10, that demonstrates only a small drift among individuals and this is a key finding for the classification stage, as we need to classify the incoming insect and we have no control on its flying mode.

One can get a variety of features out of a recording but, as analyzed thoroughly in [16–18], we believe that the unprocessed spectrum and possibly certain simple transformations of it (e.g. frequency pooling through a filter-bank, logarithmic amplitude compression) are a better choice than more sophisticated features coming from estimators (e.g. *f0*, harmonics, autoregressive features etc.) for this task. We therefore focus on the spectrum solely. Real-time verification based on the embedded circuits (see Fig 12), has to choose a simple rule, in order to be compatible with power and time constraints.

The derived spectrum of the incoming insect is compared against the spectrum of four pre-embedded spectrum prototypes. These prototypes are derived by performing K-means clustering on a set of *B. oleae* spectra different from the verification set. The K-means algorithm is a fast clustering algorithm [19] that, in our case, partitions all given spectra in groups, an represents each group with a mean spectral vector. This set is composed of 403 recordings taken with 'candlestick' sensors as demonstrated in Figs 4–5 (see S2 Multimedia for the dataset). The K-means algorithm returns the four most important cluster centers that correspond to different spectrum types of *B. oleae*. Then, the absolute distance of each of the 403 cases from the 4

prototypes is measured and the smallest distance is tracked. We calculate the distance of the amplitude spectrum of each recording from each prototype by using the absolute norm and we keep the minimum distance.

A threshold derived by the mean of the tracked distances and enlarged by 3 standard deviations is kept as the expected maximum divergence of *B. oleae* from the prototypes. During operation, if the spectrum of an incoming recording achieves a distance that is more that this threshold, it is set to a non-target species. Therefore, we need to extract threshold and reference spectra only from the target species that we definitely know, and we do not make assumptions for the incoming insects that will be unknown to us and probably different form the ones encountered in the validation set.

The data set of the target insect is composed of 230 recordings of *B. oleae* insects different from the 403 cases of the validation set. The data set is a real matrix **S** having dimensions NxM = 230x256, where N is the number of recordings, M the number of FFT coefficients, and the labels are 230 ones ("1") set for the target species. Manual inspection of several McPhail traps in the field revealed, in addition to *B. oleae*, several kind of flies, butterflies, bees, wasps, mosquitoes, *C. capitata* and even cicadas. We do not further analyze the 'everything else' case, as we are interested only on verifying the existence of a single targeted pest and therefore we set the label ("0") for them. One should be aware that species recognition of multiple species is possible and straightforward [17–18], extending the applicability of the electronic trap to various other insects of economic importance (e.g. *C. capitata*, *B. dorsalis* and many others). In order to quantify expected results we start a series of experiments with gradual increase of difficulty involving insects depicted in Table 1. For these experiments we measure the precision, recall and F1-score. Precision (P) is defined as the number of true positives (Tp) over the number of true positives plus the number of false positives (Fp).

$$P = \frac{Tp}{Tp + Fp}$$

Recall (R) is defined as the number of true positives (Tp) over the number of true positives plus the number of false negatives (Fp).

$$R = \frac{Tp}{Tp + Fn}$$

These quantities are also related to the ($F1$) score, which is defined as the harmonic mean of precision and recall.

$$F1 = 2\frac{P * R}{P + R}$$

High precision relates to a low false positive rate, and high recall relates to a low false

Table 1. Automated verification of B. oleae. Various insect species tested against the target species.

Species	No. of records
B. oleae	230
Anopheles gambiae & Culex pipiens molestus	80
Apis mellifera	205
Chironomidae family	37
Lonchaea aristella	492

doi:10.1371/journal.pone.0140474.t001

negative rate. High scores for both show that the classifier is returning accurate results (high precision), as well as returning a majority of all positive results (high recall) [19].

First, we verify the presence of *B*. *oleae* against two mosquito species *Anopheles gambiae* and *Culex pipiens molestus*. The experimental results in Fig 13 and Table 2 show very good capability of discerning *B*. *oleae* against these mosquito species. All recordings in the verification experiments with the exception of bees are derived from insects either flying in or by walking until the internal border of the trap and then flying-in (see Fig 14). One should have in mind that this was not a difficult scenario as the spectrum of a typical case of a *B*. *oleae* wingbeat is lower in frequency content than the high pitched mosquitoes (see Fig 15).

The next experiment deals with a species that is closer to the frequencies of *B*. *oleae*, and this is *A*. *mellifera* (the western honey bee). Bees produce low frequency wingbeats with a fundamental frequency around 140–150 Hz which is lower than the 190–210 Hz of *B*. *oleae* as seen in Fig 16. Their harmonics, however, are partially overlapping. The capability of the system to discern which is which is again very high as shown in Fig 17 and Table 3.

We then move to the *Chironomidae* family of non-biting midges. This family's wingbeat spectrum shares partial resemblance to B. oleae but with a very different distribution of power in its higher harmonics (see Fig 18).

The capability of the system to discern among insects is again adequate as shown in Fig 19 and Table 4, although we see a drop in the classification accuracy as the different spectra converge to common shapes.

Then we move to a difficult case: *Lonchaea aristella* (Diptera: Lonchaeidae) is a fly as *B*. *oleae* with a wingbeat spectrum completely overlapping with *B*. *oleae* (see Fig 20).

In this case the decision rule to classify insects based on the absolute difference of the spectra fails to discern the different species. This is due to the fact that the fundamental frequency, that is almost the same in both species, dominates in the calculation of the distance measure. Thus, we examine other ways of classifying the spectrum that do not rely on a single number. First, we resort to off-line, model-based classification that bases the decision to more parameters than a single threshold, and is able to capture variable interactions to a large depth. Off-line pattern recognition may classify data using more computational demanding algorithms, in order to set a rough limit of what can be achieved on the specific dataset, provided we had no memory and power constraints. Since we have fixed our approach to rely exclusively on the spectrum and its transformations, we employed well-established, state of the art, machine learning techniques that are capable of dealing with high-dimensional datasets. Support Vector Machines (SVM), Random Forests (RF), Randomized Trees Classifiers, as well as the Gradient Boosting Classifier (GBC), are known to be able to handle efficiently high dimensional feature sets [19]. We tried these classification techniques in the case of *B*. *oleae* against *L*. *aristella* to see if model based techniques can squeeze out more information from this data. The dataset is randomly shuffled prior to any classification. The verification scores are based on 10-fold cross-validation and are reported in Table 5.

For each fold we retain 80% from the shuffled data and accuracy is measured on the unseen 20% of the data. Table 1 depicts the mean results over the random 10-folds. The results are now more encouraging as depicted in Fig 21 and Table 6. For this difficult case we also tried deriving 2 codebooks of 4 prototypes, one codebook for each species (see Fig 22 and Table 7).

As detailed in the Methods section, we developed a web backend to allow trap monitoring in a user friendly and timely manner. A proof-of-concept of this can be seen in Fig 23, where for each of the monitored traps we have introduced a set of freeboard.io panels presenting the number of events, the number of matches, the environmental parameters (including a graph showing the past values), a map with the trap's exact location and, finally, the current battery

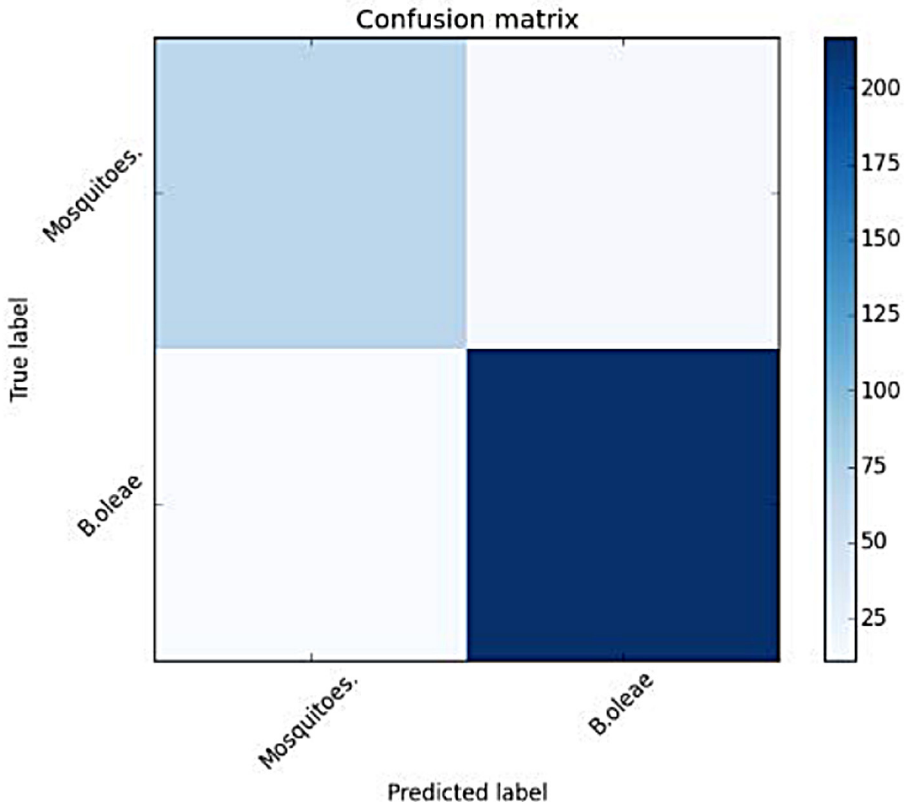

Fig 13. Confusion matrix of Verification results of *B. oleae* vs *Mosquitoes* (*A. gambiae* and *C. pipiens molestus*).

doi:10.1371/journal.pone.0140474.g013

status. The above are all updated in real-time, as soon as the data uploads new information to dweet.io.

In the example of Fig 23, the 1st trap has recorded 23 events through its sensors, of which 3 were positively identified as *B. oleae* insects. Moreover, the environmental sensors have recorded a temperature of 29°C and a humidity of 69%, the GPS sensor has also acquired the exact location and the battery is at 81% of its full capacity. To upload the above data online, the software embedded in the trap issued HTTP requests to the dweet.io servers, at predefined intervals. The data were saved on the dweet.io servers, under the "entomatic_trap_1", "entomatic_trap_2" and "entomatic_trap_3" object names. The same data were consequently aggregated and visualized in the freeboard.io panels created for this purpose.

Discussion

The results show that the current state of the verification module can deliver reliable counts of insects, provided their spectra do not overlap significantly. One should note, that, in the

Table 2. Accuracy measures for classifying *B.oleae* against two mosquito species.

	precision	recall	F1 score	#recs
B.oleae	0.95	0.94	0.95	230
Mosquitoes	0.84	0.86	0.85	80
Avg/total	0.92	0.92	0.92	310

doi:10.1371/journal.pone.0140474.t002

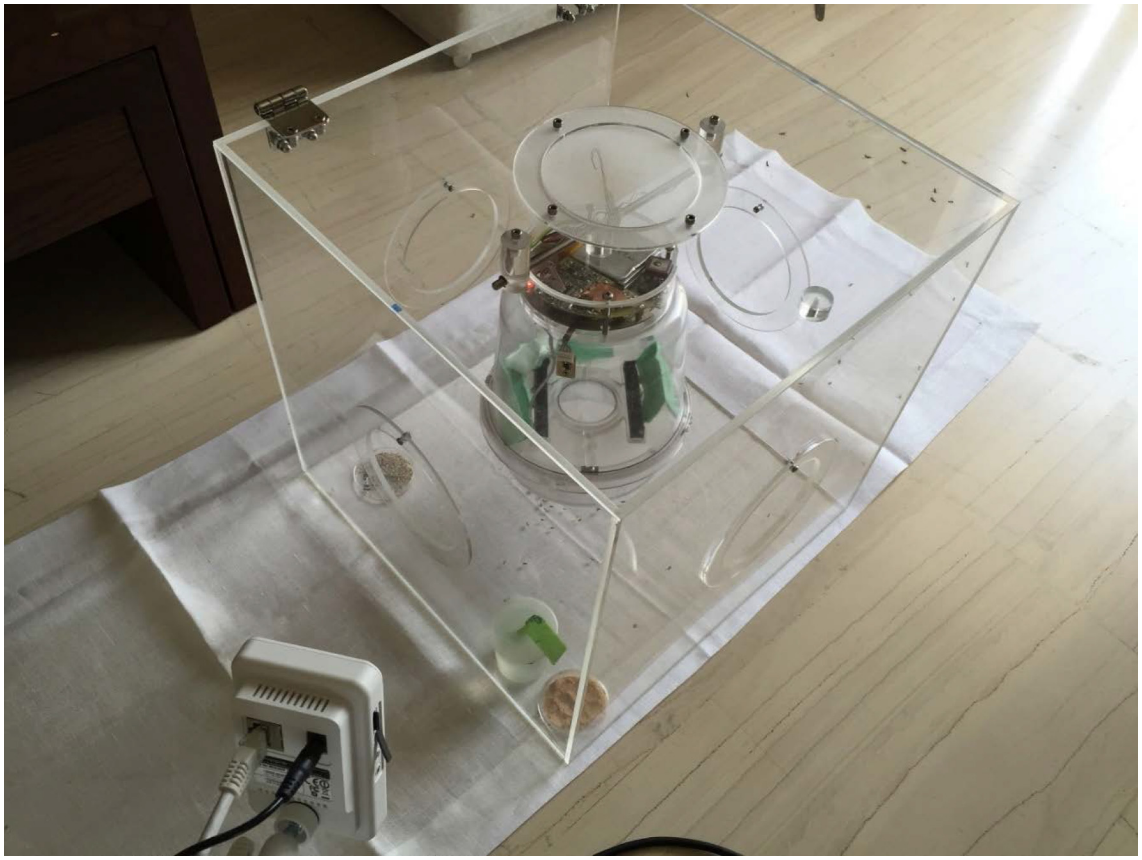

Fig 14. The electronic McPhail trap inside an insectary cage with *B. oleae*. To help interpret system operating characteristics, the analog output from the sensor is recorded before it is sent to the microcontroller, and recordings are also made after the signals are processed by amplifier and filter circuits.

doi:10.1371/journal.pone.0140474.g014

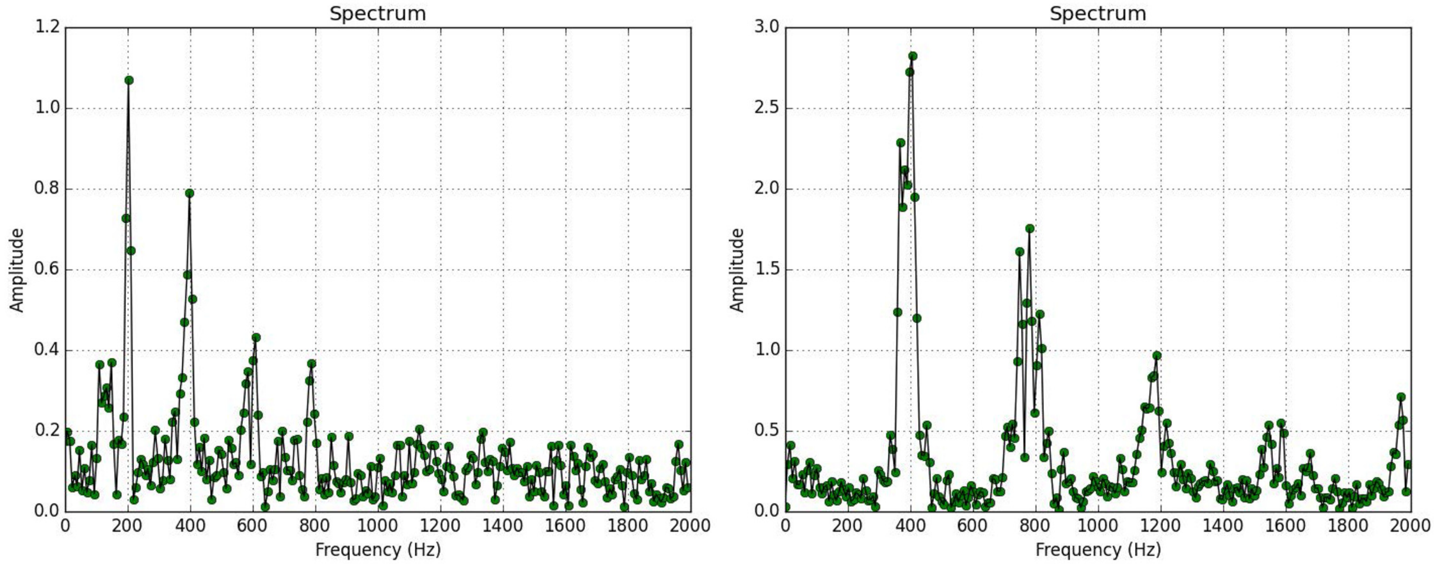

Fig 15. Spectrum of wingbeat recording of insect flying in the trap. (Left) *B.oleae*, **(right)** *A. gambiae*.

doi:10.1371/journal.pone.0140474.g015

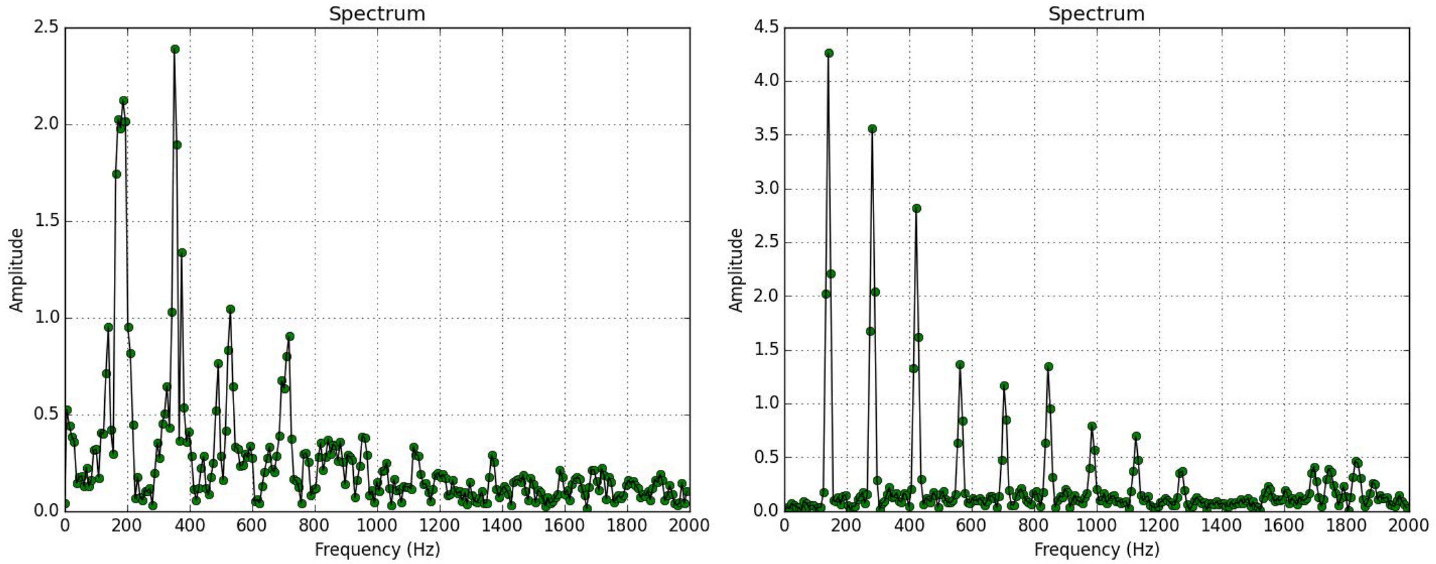

Fig 16. Spectrum of wingbeat recording of insect flying in the trap. (Left) *B.oleae*, **(right)** *A. mellifera*.

doi:10.1371/journal.pone.0140474.g016

aforementioned experimental results we have chosen a very simple rule based on the absolute distance of spectra. The model-based classification experiments demonstrate that there is room for significant improvement in the classification scores. We have scrutinized the analog

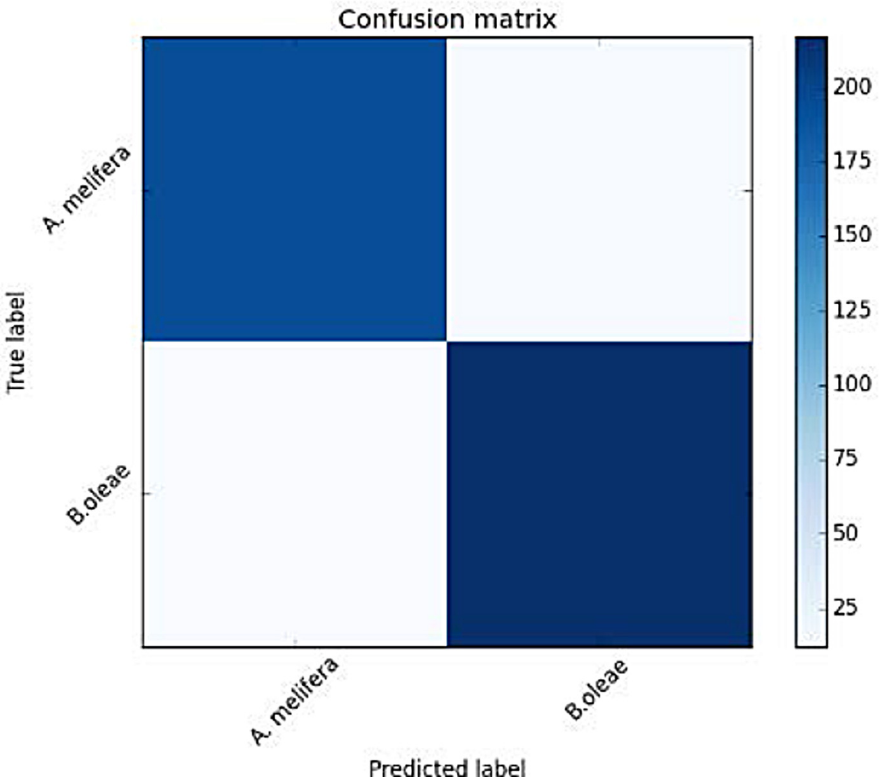

Fig 17. Confusion matrix of Verification results of *B. oleae* vs *A. mellifera*.

doi:10.1371/journal.pone.0140474.g017

Table 3. Accuracy measures for classifying *B.oleae* against *A. mellifera*.

	precision	recall	F1 score	#recs
B.oleae	0.95	0.94	0.95	230
A. mellifera	0.94	0.94	0.94	205
Avg/total	0.94	0.94	0.94	435

doi:10.1371/journal.pone.0140474.t003

recordings prior to entering the microprocessor and after being digitized by it and we see a positive perspective in the following directions: a) increasing the coding depth of the A/D converter to 16 bits (currently restrained to 10 because of the processor used) as coding affects recognition greatly, b) the buffer of monitored samples used to trigger an event is currently discarded which reduces the available data from which the spectrum is derived. Since our data are short-time, this, sometimes leads to very noisy recordings as parts of the events were discarded. A number of misclassification was due to this fact and we will account for this loss of data in future versions, c) studying 2D receptors (i.e. two layers of photoreceptors instead a single array to allow for a longer time-span of flight (at least-double) that is expected to increase spectrum accuracy, d) transmit the recordings to a central agency instead of the decisions in text. Since typical events last only 30–50 ms this means that 1 second can hold about 20–30 events at 4 kHz sampling rate. The transmission using the General Packet Radio Services (GPRS) functionality will allow the more accurate, off-line, model-based techniques to be applied on the recordings while significantly reducing the cost of the trap. The latter choice is expected to shift recognition results by a large margin.

Conclusions

The effort of this work was to make a functional, stand-alone, prototype electronic trap that completes successfully all processing stages starting from attracting *B.oleae*, as all traps do, measuring the wingbeat of the entering insect, extracting its spectrum, comparing it to pre-embedded reference patterns in real-time and transmitting collective results via SMS.

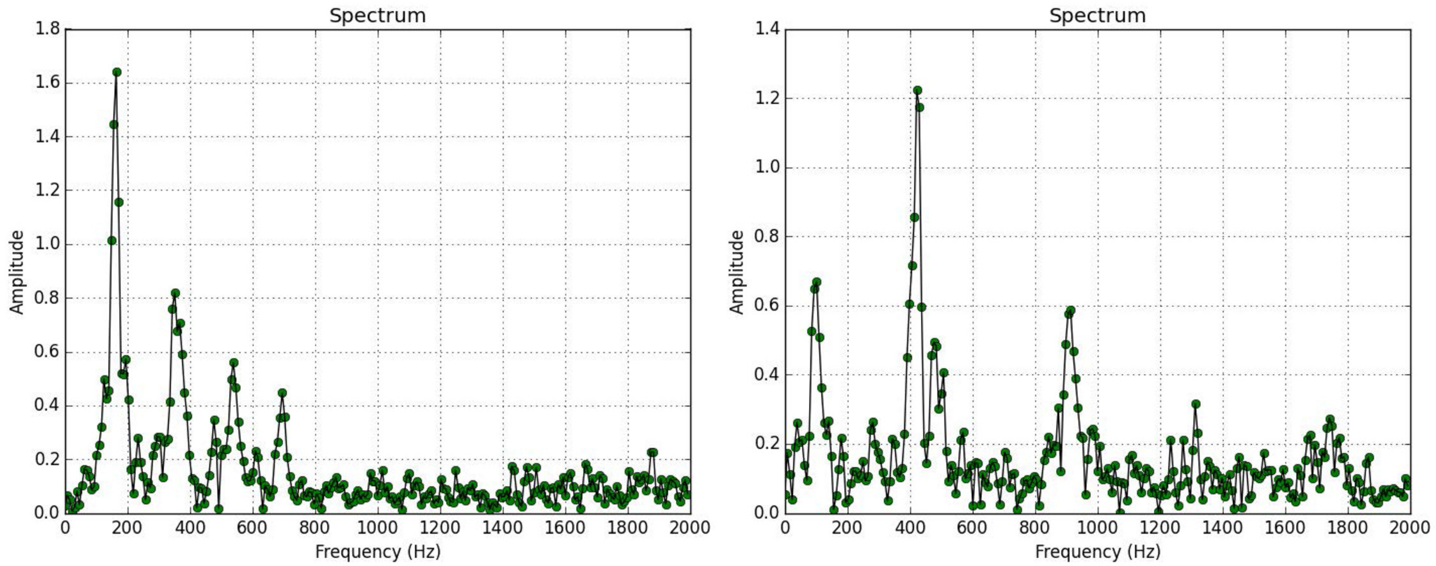

Fig 18. Spectrum of wingbeat recording of insect flying in the trap. (Left) *B.oleae*, **(right)** member of the *Chironomidae* family.

doi:10.1371/journal.pone.0140474.g018

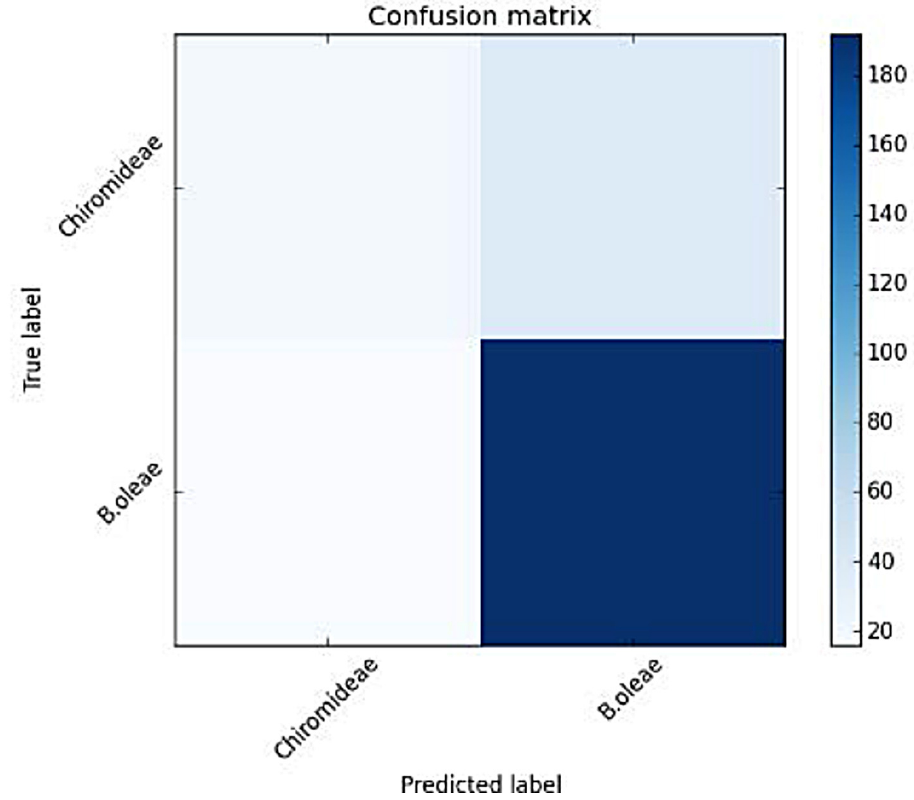

Fig 19. Confusion matrix of Verification results of *B*. *oleae* vs *Chironomidae*.

doi:10.1371/journal.pone.0140474.g019

The electronic trap, at this stage, can adequately discern *B*. *oleae* and deliver reliable counts against species that are very different in morphological terms, which, in-turn, is reflected on their wingbeat spectrum. As we move to species that resemble *B*. *oleae* fly (e.g. other kinds of flies: *M*. *domestica*, *L*. *aristella*, *C*. *capitata*) the recognition accuracy drops and we must resort to more advanced classifiers as analyzed in the *Verification experiment*s Section.

There are some issues that are not dealt in this work but will be in the near future:

a. Power sufficiency is something missing from the current analysis of the electronic trap. We are working on low-power electronics, in order to make the trap power sufficient for months and also reduce its actual cost (see Appendix).

b. A pending task is the experimentation with gender recognition. In the case of mosquitoes, that are dimorphic, classifying sex from the wingbeat is a relatively easy task as females are larger than males and this leaves a trace in the wingflap as demonstrated in [17]. For the case of *B*. *oleae* we have not yet studied this, as it requires the manual separation of hundreds of *B*. *oleae* adults in different cages for males and females.

Table 4. Accuracy measures for classifying *B*.*oleae* against members of the *Chironomidae* family.

	precision	recall	F1 score	#recs
B.oleae	0.90	0.94	0.92	230
Chironomidae	0.48	0.32	0.39	37
Avg/total	0.84	0.86	0.85	267

doi:10.1371/journal.pone.0140474.t004

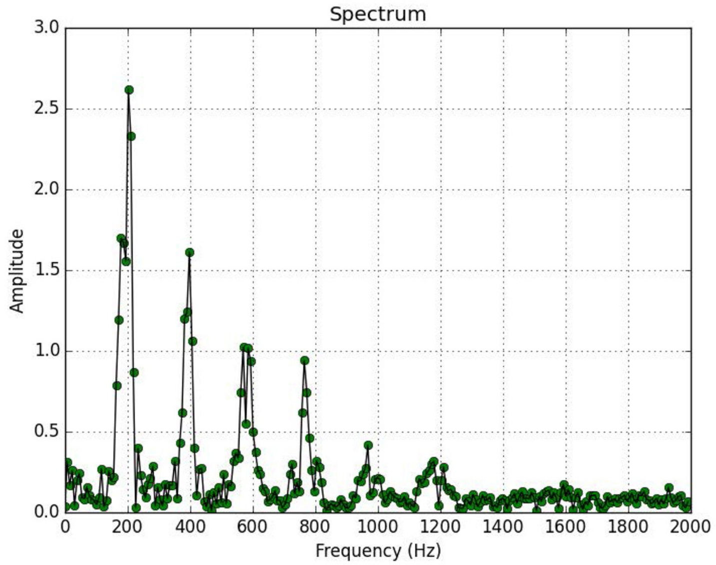

Fig 20. Spectrum of wingbeat recording of insect flying in the trap. (Left) *B.oleae*, **(right)** *Lonchaea aristella* (Diptera: Lonchaeidae).

doi:10.1371/journal.pone.0140474.g020

c. *B. oleae* is ectothermic and increases its wingbeat frequency along with the rise in temperature. The trap must operate in temperatures typically between 20–35°C. We experiment on compensating the effect of varying temperatures by shifting the spectrum before comparing it to embedded reference signals according to the measurements of the temperature sensor embedded in the trap. We also experiment with embedding prototypes recorded at different temperatures.

d. We are currently experimenting with the idea of wirelessly sending recordings of the pest to be installed automatically in the trap and serve as prototypes i.e. take reference signals from an internet host of insects' wingbeats measured with optoacoustic systems and download the prototypes remotely straight to the traps. This would augment the utility of the trap as it would be able to change focus on different insects without human intervention.

e. The real field holds the truth regarding the functionality of the electronic traps and a detailed open-field test is pending. However, in-lab analysis with almost real-field conditions was the necessary step prior to the unconstrained field as it revealed possible problems and permitted the fine-tuning of software and equipment.

We believe that biometrics can be applied in various ways on animals, including insects, in order to realize what happens and where it happens and what is the density of the species

Table 5. Model-based techniques classifying B. oleae vs L. aristella. Average scores over 10-fold cross-validation, 20% hold-out.

	10-fold average
Support Vector Machines	0.738
Random Forests	0.736
Extra Trees	0.725
Gradient Boosting Classifier	0.736

doi:10.1371/journal.pone.0140474.t005

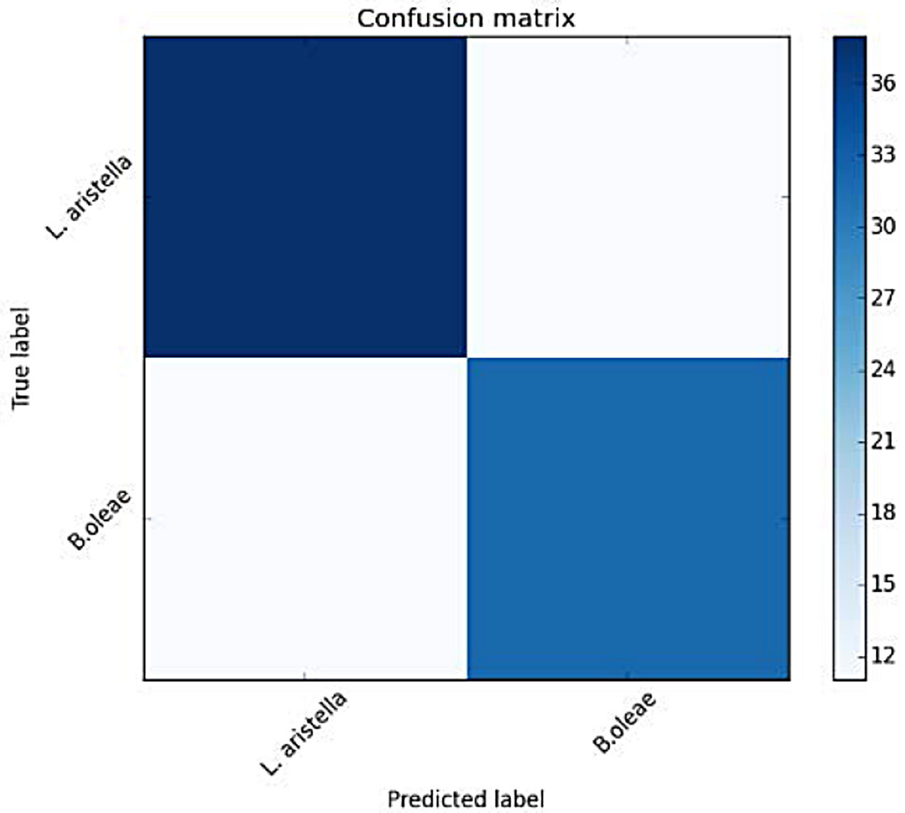

Fig 21. Confusion matrix of verification results using a random forest classifier (*B*. *oleae* vs *L*. *aristella*) accuracy measures.

doi:10.1371/journal.pone.0140474.g021

Table 6. Accuracy measures for classifying *B*.*oleae* against *L*. *aristella*.

	precision	recall	F1 score	#recs
B.*oleae*	0.74	0.74	0.74	43
L. *aristella*	0.78	0.78	0.78	49
Avg/total	0.76	0.76	0.76	92

doi:10.1371/journal.pone.0140474.t006

counted, allowing the design of reliable policies based on the outcome of these measurements. We suggest that electronic traps that record and analyze insects' wingflap can open new ways for several other applications such as smart beehives, devices that emit species labels and counts to central agencies to feed with data infestation models updated real-time and make predictions of future outbreaks (see e.g. https://www.kaggle.com/c/predict-west-nile-virus), alert devices for dangerous species and countless others applications.

Appendix

We hereinafter include a cost analysis of the electronic trap.

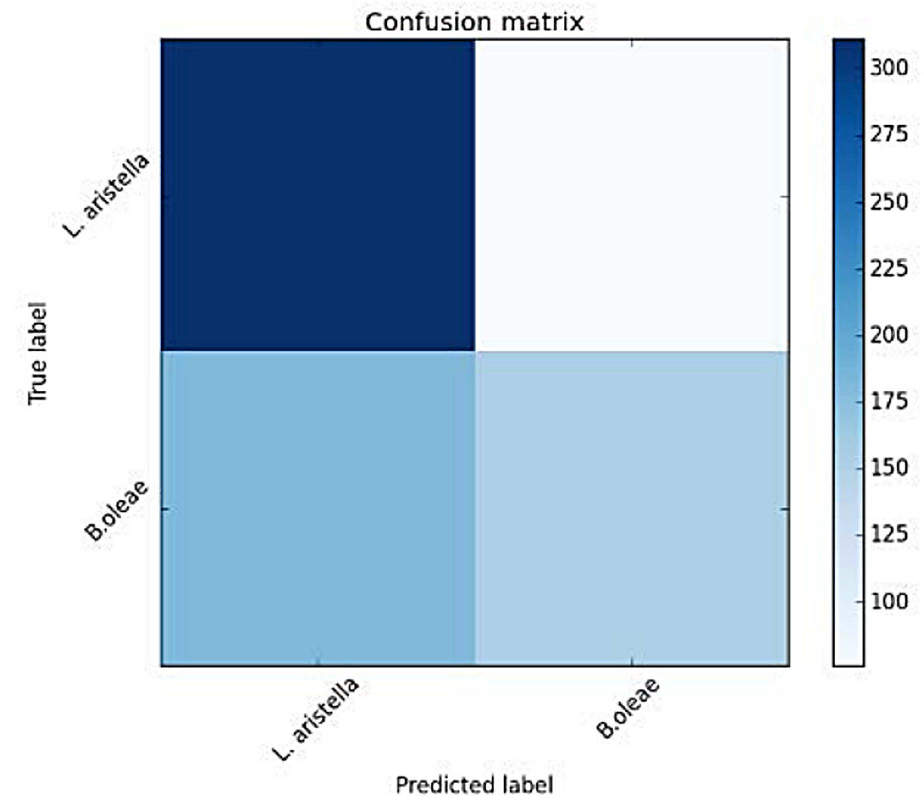

Fig 22. Confusion matrix of verification results using the absolute distance between spectra and a codebook of 4 spectrum prototypes for each species *B*. *oleae* vs *L*. *aristella*.

doi:10.1371/journal.pone.0140474.g022

Table 7. Accuracy measures for classifying *B*.*oleae* against *L*. *aristella* using a codebook of 4 spectrum prototypes.

	precision	recall	F1 score	#recs
B.*oleae*	0.61	0.38	0.47	230
L. *aristella*	0.59	0.79	0.68	262
Avg/total	0.60	0.60	0.58	492

doi:10.1371/journal.pone.0140474.t007

Cost-analysis

There is no reason nowadays to base monitoring of insects on expensive and fragile *glass* McPhail traps as often reported in entomologically oriented research work. Even though there is evidence that plastic McPhail type trap have similar performance in catching insects as the original glass McPhail [20] one could even insist to the original design of the McPhail by printing an indistinguishable plastic one using conveniently a 3-D printer.

Regarding the electronic version of the McPhail trap presented in the paper, we believe that the quality and value of olive oil and the high-risk due to the pest *B*. *oleae*, can justify the added costs of the electronic monitoring traps. The cost is of course, subject to change but in order to help to the assessment of the cost/benefit tradeoff we have a detailed cost breakdown in Table 8. It should be noted that the total cost is expected to drop if the prototype uses the

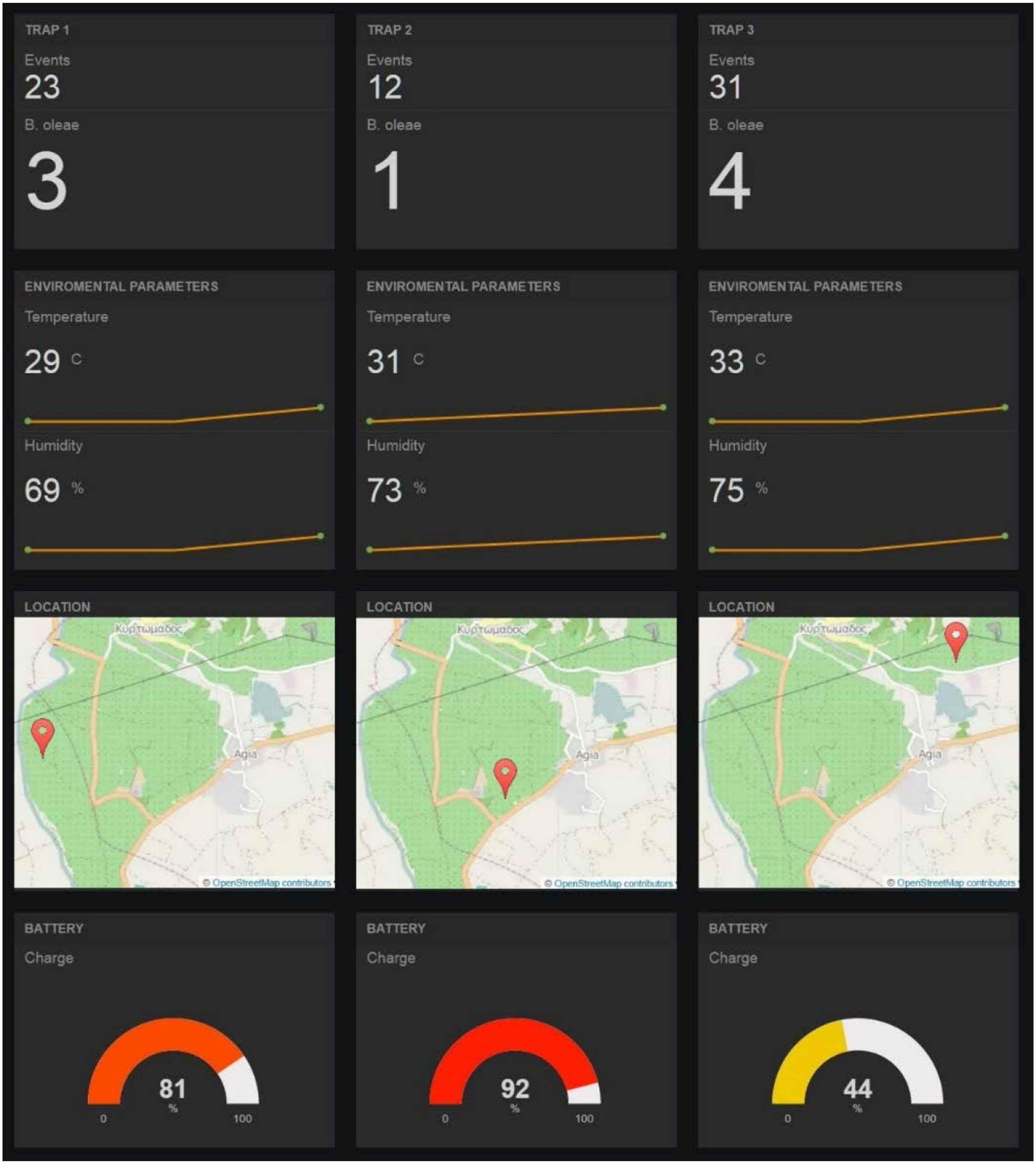

Fig 23. Detection counts received from the GPRS module. The figure shows the online web interface that presents detection results of trapped insects in general and target species in particular, based on Freeboard.io and OpenStreetMap. (Figure is similar but not identical to the original image, due to copyright restrictions, and is therefore for representative purposes only).

doi:10.1371/journal.pone.0140474.g023

Table 8. Cost breakdown of the hardware of the electronic McPhail trap (date last viewed 30/6/2015).

Item	Model	Unit Price €	Quantity
Emitter (infrared led)	TCRT5000	0.45	4
Receiver (diodes)	TEMD5080X01	0.68	10
Microcontroller	ATMega2560	12.15	1
Temperature-humidity	Si7021	4.03	1
GSM/GPS	SIM908	15	1
Electronic Components	Passive, RTC, EEPROM, PCB, Connectors	15	1
Plastics	Plastic McPhail trap, add-on	5	1
Battery	Lithium 6000mAh	19	1
	**Total**	**78.78**	

doi:10.1371/journal.pone.0140474.t008

GPRS functionality instead of GPS and the microcontroller changes to one with lower power consumption.

Supporting Information

S1 Fig. Candlestick sensor design.
(TIF)

S2 Fig. Candlestick sensor.
(TIF)

S3 Fig. Candlestick sensors (two photodiodes and two phototransistors arrays).
(TIF)

S4 Fig. Candlestick sensor in an insectary cage.
(TIF)

S5 Fig. Candlestick sensor in an insectary cage.
(TIF)

S6 Fig. Candlestick sensor in an insectary cage.
(TIF)

S7 Fig. Candlestick sensor in an insectary cage.
(TIF)

S8 Fig. Candlestick sensor in an insectary cage.
(TIF)

S9 Fig. Candlestick sensor in an insectary cage.
(TIF)

S10 Fig. Candlestick sensor in an insectary cage.
(TIF)

S11 Fig. Candlestick sensor in an insectary cage.
(TIF)

S12 Fig. Candlestick sensor in an insectary cage.
(TIF)

S13 Fig. Candlestick sensor in an insectary cage.
(TIF)

S14 Fig. Candlestick sensor in an insectary cage.
(TIF)

S15 Fig. Candlestick sensor in an insectary cage.
(TIF)

S16 Fig. Candlestick sensor in an insectary cage.
(TIF)

S17 Fig. Candlestick sensor in an insectary cage.
(TIF)

S18 Fig. Candlestick sensor in an insectary cage.
(TIF)

S19 Fig. Candlestick sensor in an insectary cage.
(TIF)

S20 Fig. Photodiodes and phototransistors arrays (receivers) followed by infrared led arrays (emitters).
(TIF)

S21 Fig. Infra-red LED emitter circuit. Light is provided by 4 infrared LEDs (940nm) connected in row, led by a 2.7 mA current.
(TIF)

S22 Fig. Photodiodes Array Receiver circuit. The receiver is a linear array of 10 photodiodes connected in parallel. The received light is amplified by the IC1 and is driven to the band-pass filter.
(TIF)

S23 Fig. Circuit of filters. The filtering process is carried out by the high-pass filter IC7 A, B & C and the low-pass filter IC6 A, B & C. Subsequently, the signal is amplified by IC6D and driven to the processor.
(TIF)

S24 Fig. Gain circuit. The signal coming out of the filters is amplified by the current circuit by the factors 1, 10 and 100. The signals x1, x10 and x100 are driven to the 3 analog inputs of the microcontroller.
(TIF)

S25 Fig. Power supply circuits. The power supply circuit has as input the Lithium battery 3.7 Vdc and supplies voltages 3.3 Vdc & 5 Vdc for the digital and analogue circuits.
(TIF)

S26 Fig. Communications circuits. The GSM module sends text data through GPRS. It also embeds a GSM. When not in use MOSFET Q2 cuts its power supply so it does not consume energy. It is controlled via the Microcontroller (ATMEGA2560).
(TIF)

S27 Fig. SD circuits. In the SD card the microcontroller stores the recordings (we store the FFT but the actual recording of the ADC can be also stored) for further analysis. When not in use MOSFET M7 cuts its power supply so it does not consume energy.
(TIF)

S28 Fig. Processor circuit. Processor IC1, receives the analog signal from the inputs A0, A1 and A2 and selects the proper gain. IC1 is processing the resulting digital signal. It also controls the GSM module, the humidity and temperature sensors.
(TIF)

S29 Fig. RF circuit. Wireless transmission of the recording of the optoelectronic sensor prior to its entrance to the microcontroller.
(TIF)

S30 Fig. CAD design of trap.
(TIF)

S31 Fig. CAD design of trap.
(TIF)

S32 Fig. CAD design of trap.
(TIF)

S33 Fig. CAD design of trap.
(TIF)

S34 Fig. CAD design of trap.
(TIF)

S35 Fig. CAD design of trap.
(TIF)

S1 Multimedia. Wav recordings of several insects using optoacoustic sensors.
(ZIP)

S2 Multimedia. Spectrogram of flying insects' wingbeat as stored in the SD of the electronic McPhail trap and presented in the validation section of this study.
(ZIP)

S1 Video. Wingbeat of tethered *M. domestica*. Sound is the sonification of the optoacoustic sensor played through loudspeakers. Photodiodes array, Laser emitter as in [14].
(M4V)

S2 Video. Real-time spectrum analysis of tethered *C. capitata* wingbeat. Photodiodes array, infrared emitter.
(WMV)

S3 Video. Animation of composing an automatic McPhail from its constituent parts.
(WMV)

S4 Video. The electronic McPhail in operational mode.
(M4V)

Acknowledgments

We acknowledge Anastasia Kambouraki, from the Department of Biology, University of Crete, Heraklion, Greece for patiently collecting insect specimens and taking care of insect cultures for the recording experiments and Prof. John Vontas head of the Laboratory of Molecular Entomology in the same department for granting us access to all insectaries and associated equipment. Plastic McPhail type traps and attractants were provided by PHYTOPHYL-N.G. Stavrakis—Insect Attractants & Insect Traps (http://www.phytophyl.gr/). Theodoros

Papadoulis from the Technological Educational Institute of Crete, Department of Environmental and Natural Resources and Engineering made the CAD representation of the trap as well as all modification to the plastic McPhail type trap. We acknowledge Panagiotis Eliopoulos from the Technological Educational Institute of Larissa and Dimitris Kontodimas from Benaki Entomopathological Institute for manual classification of insects found in the field.

Author Contributions

Conceived and designed the experiments: IP. Performed the experiments: IP KF IR. Analyzed the data: IP KF IR. Contributed reagents/materials/analysis tools: IR. Wrote the paper: IP KF.

References

1. Plant Health Australia. The Australian Handbook for the Identification of Fruit Flies. 2011, Version, 1.0. Plant Health Australia. Canberra, ACT.
2. Kent D, Marshall J. Olive Fruit Fly: Managing an Ancient Pest in Modern Times, Annual Review of Entomology, 2010; 55: 151–169. doi: 10.1146/annurev.ento.54.110807.090553 PMID: 19961328
3. Neuenschwander P, Michelakis S. Olive fruit drop caused by Dacus oleae (Gmel.) (Dipt. Tephritidae). Journal of Applied Entomology. 1981; 91, 1–5, 193–205.
4. Nardi F, Carapelli A, Dallai R, Roderick GK, Frati F. Population structure and colonization history of the olive fly, Bactrocera oleae (Diptera, Tephritidae), Mol Ecol. 2005; 14 (9):2729–38. PMID: 16029474
5. Michelakis SE, Neuenschwander P. Cavalloro R. Estimates of the crop losses caused by Dacus oleae (Gmel.)(Diptera, Tephritidae) in Crete, Greece. Fruit Flies of Economic Importance. Rotterdam: AA: Balkema., 1983, 603–611.
6. Kapatos E., and Fletcher B. An assessment of components of crop loss due to infestation by Dacus oleae in Corfu. Entomologica Hellenica, 1983, 1:7:16.
7. White I, Elson-Harris M. Fruit flies of economic importance: their identification and bionomics. CAB International, 1992, Wallingford, United Kingdom.
8. Jiang Joe-Air, Tseng Chwan-Lu, Lu Fu-Ming, Yang En-Cheng, Wu Zong-Siou, Chen Chia-Pang, et al,. A GSM-based remote wireless automated monitoring system for field information: A case study for ecological monitoring of the oriental fruit fly, *Bactrocera dorsalis* (Hendel), Computers and Electronics in Agriculture, 2008; 62, 2, 243–259, ISSN 0168-1699, http://dx.doi.org/10.1016/j.compag. 2008.01.005.
9. Moore A, Miller J, Tabashnik B, Gage S. Automated identification of flying insects by analysis of wingbeat frequencies. Journal of Economic Entomology, 1986, 79(6):1703–1706. doi: 10.1093/jee/79.6.1703
10. Kirsch P, Czokajlo D, McLaughlin J, Moore A, Haynes K, Fadamiro HY. Automated remote identification and monitoring of moths in flight. 80th Annual Western Orchard Pest and Disease Management Conference, 2006.
11. Potamitis I.; Rigakis I.; Fysarakis K. The Electronic McPhail Trap. Sensors, 2014, 14, 22285–22299. doi: 10.3390/s141222285 PMID: 25429412
12. Moore A. Artificial neural network trained to identify mosquitoes in flight. J. Insect Behavior. 1991, 4(3), 391–396.
13. Ellington C. The novel aerodynamics of insect flight: applications to micro-air vehicles. Journal of Experimental Biology, 1999; 202(Pt 23):3439–48. PMID: 10562527
14. Potamitis I.; Rigakis I. (2015). Novel Noise-Robust Optoacoustic Sensors to Identify Insects through Wingbeats. IEEE Sensors Journal, 2015; 15, no.8, 4621,4631, Aug. 2015, doi: 10.1109/JSEN.2015.2424924
15. Engel JE, Wyttenbach RA. An optoelectronic sensor for monitoring small movements in insects. Florida Entomologist. 2001; 843: 336–343.
16. Potamitis I., Fysarakis K., Longueville D., Ntalampiras S. Hardware Implementation of a system classifying the optoacoustic signature of insects' wingbeat. The 22nd International Congress on Sound and Vibration, Florence (Italy), 2015, 12–16 July.
17. Potamitis I. Classifying insects on the fly. Ecological Informatics, 2014, 21, 40–49, ISSN 1574-9541, http://dx.doi.org/10.1016/j.ecoinf.2013.11.005.
18. Potamitis I, Schäfer P. On classifying insects from their wingbeat: New results. Ecology and acoustics: emergent properties from community to landscape, 2014, Paris, France.

19. Pedregosa F., Varoquaux G., Gramfort A., Michel V., Thirion B. and Grisel O et al. Scikit-learn: Machine Learning in Python. Journal of Machine Learning Research, 2011; 12: 2825–2830.
20. Barros M., Amaral P., Malavasi A. (1991). Comparison of glass and plastic McPhail traps in the capture of the south American fruit fly, *Anastrepha fraterculus* (Diptera: Tephritidae) in Brazil. Florida Entomologist, 1991; 74:3, 467–468.
